# Towards smart sustainable cities using Li-Fi technology: geo-location infrastructure utilizing LED street lights

**DOI:** 10.7717/peerj-cs.1009

**Published:** 2022-07-21

**Authors:** Lamya Albraheem, Lamia Alhudaithy, Afnan Aljaser, Muneerah AlDhafian, Ghada Bahliwah

**Affiliations:** Department of Information Technology, College of Computer and Information Science, King Saud University, Riyadh, Saudi Arabia

**Keywords:** Li-Fi, Visible light communication, VLC, Light fidelity, Light emitting diodes, Photo-detector, Smart city

## Abstract

**Background:**

Cities are shifting toward providing more efficient services and the Internet of Things (IoT) becoming the future of things. The shift toward using eco-friendlier LED lights in lighting cities is another genuine game-changer in the future of Light Fidelity technology (Li-Fi). Li-Fi is a visible light communication (VLC) technology that uses Light Emitting Diodes (LED) bulbs for communication. The utilisation of thousands of light sources around a city acting as wireless access points and delivering location-based content will shift cities towards being smart sustainable cities. Recently, this technology got huge attention from the research community and different research has been conducted to improve this filed. However, there is a noticeable need to develop real-world systems that utilise Li-Fi technology.

**Methods:**

This article aims to contribute to developing a Geo-Li-Fi system that uses LED lights to provide the services for collecting contextual data and delivering location-based services (LBS) in different areas of the city. The system is described along with details of its design, implementation and development. Moreover, the overall set-up of the testbed that used to evaluate the proposed system is presented. In addition, an experiment is conducted using a real-world scenario to test the functionality of the system and report the outputs.

**Results:**

The effect of the system is discussed according to different aspects of sustainability which include economic, social and environmental aspects. The system was tested in indoor and outdoor environments, and it can be seen that the sunlight does not affect the ability of LEDs to deliver the content during the daytime. Regarding the transmission range of the LED lamp, it can be seen that it is affected by different factors. It depends mainly on the power of lamp, so it will be increased significantly when the power of LED is increased. Also, an increase in the beam angle will result in wider coverage area which affected by the intensity.

## Introduction

The Internet of Things (IoT), or in other words, “Network of Things”, is the extension of internet connectivity beyond traditional devices (Aswin et al., 2016). The concept of IoT refers to the integration of objects into the virtual world, forming up a network of connected intelligent things. In IoT, an object becomes aware of its surroundings by giving it the power to communicate, identify itself and transfer data to other things without human’s intervention. Merging objects to the virtual world of internet could be achieved through empowering physical items with communication and computing technologies, allowing them to act as internet access points (Rose, Eldridge & Chapin, 2015). Since IoT is all about connectivity, information gathering and sharing, it is reasonable to say that a smart city is a connected city. Smart cities use the latest innovations in the Internet of Things to become more livable, economically sound, resilient and sustainable (Philips, 2017). IoT is considered as a key enabler of smart cities. Therefore, a smart city could also be defined as an interconnection of IoT applications integrated in different urban services to enhance their quality and performance. Infusing municipal services with intelligence and automation of IoT’s smart technologies is genuine transformation in urban areas.

Specifically concerning the applications of IoT in smart energy management, the reduction of energy consumption is an issue of matter to metropolis, resolved by smartening cities. Different solutions are available in regards of achieving this purpose. A considerable solution is enabling Light Emitting Diodes (LED) for lighting in order to limit energy consumption. LED lights have proven their efficiency in energy savings, they acquire long lasting lifespan and provide better illumination. Those appealing features led the transition toward illuminating buildings, parks, public transportation and streets with LED bulbs, fulfilling the resource utilization objective of IoT. Fortunately, energy saving is not the only tempting benefit of LED lights. It has been proven that the LED technology has the ability to act as light sensor, giving it the capability to transmit and receive data. In fact, this particular usage of LED lights is the core of our contribution. The utilization of LED lights as wireless data transceivers is known as Light-Fidelity (Li-Fi) technology.

**Figure 1:**
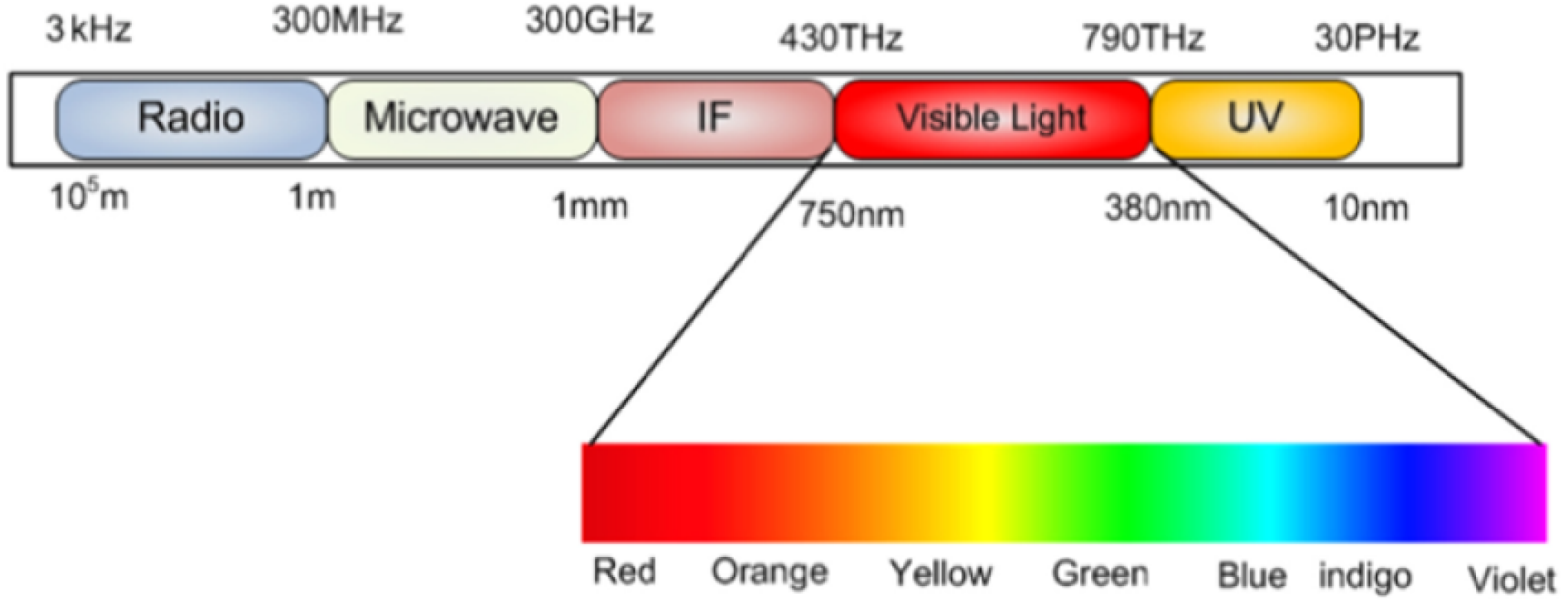
VLC frequency spectrum (Khan, 2017; CC BY-NC-ND 4.0).

Li-Fi, an acronym of “Light-Fidelity” is a visible light communication (VLC) technology in which it employs LED (Light-Emitting Diodes) bulbs for illumination and data transmission. The VLC is a version of data communication systems which leverages visible light for communication. It occupies a band in the Electromagnetic (EM) spectrum from 380 nm to 750 nm corresponding to a frequency spectrum of 430 THz to 790 THz as shown in Fig. 1 (Khan, 2017). Since the visible light spectrum is 10,000 times larger than the radio frequency spectrum, VLC is regarded as a solution to the RF bandwidth limitations (Khan, 2017). The Li-Fi technology can transmit the wireless data through illumination (Shuriji, 2014). There is a need for two main components to get a Li-Fi system which are: data transmitter (*i.e.,* LED light), and data receiver (*i.e.,* photo-detector). Li-Fi overcomes several inefficiencies of the current wireless communication technologies using Radio Frequency (RF) band. One of the biggest struggles of the RF band is known as “Spectrum Crunch”, which is the incapability to keep up with the huge demand of wireless communication and high data rates due to limited bandwidth (Manral & Singh, 2016). Interference sufferings, low data rates and human safety concerns are other significant downgrades for the RF band. Li-Fi, on the other hand, is a preceding technology with high data transmission speeds, safety for human use and security. Moreover, the integration of illumination and data communication services from the same source reduces the infrastructure complexity and energy consumption significantly (Yadav et al., 2016).

Serious efforts have been taken by multiple firms and research labs around the world in order to improve the Li-Fi technology and makes it available to the public outside laboratory doors. Regardless of these efforts, there’s still a notable need to support the technology’s growth on both research and application levels. Therefore, this paper aims to contribute in fulfilling those needs. It aims to utilize lights for more than illumination by developing a Li-Fi-compatible system designed for smart cities as an application of IoT to provide Location based Service (LBS) service.

This paper contributes with the following:

• Developing a Li-Fi-based IoT framework that utilizes light sources to collect and deliver huge amounts of geo-contextualized data for further analysis and processing. The content can be news, advertisements, awareness message, etc. that can be published or collected from different areas of the city utilizing Li-Fi technology.
• Presenting the design process of the proposed Geo-Li-Fi system.
• Showing the implementation and development details of Geo-Li-Fi System.
• Describing the overall setup of the testbed that has been used to evaluate system’s functions.
• Conducting an experiment that use a real world scenario to test the functionality of the proposed system.
• Discussing the effects of the proposed system towards designing a smart sustainable city.

The reminder of the paper is organized as follows: Section 2 discuss impact of Li-Fi technology on the sustainability. Section 3 briefly the related works, Section 4 describes the architecture of the proposed system which includes system design and its main components. The implementation and development details are presented in Section 5. Section 6 presents the technical setup of the testbed. Also, an experiment that used a real-world scenario was conducted and the results are reported in Section 7. Section 8 present the results of the usability testing that performed for the proposed system. Section 9 discuss the how the proposed system can develop a smart sustainable city. Section 10 shows the discussion and observations of this paper. Finally, we conclude the article and present future work in Section 11.

## Li-Fi Technology and Sustainability

### Impact of Li-Fi on sustainability

The main source of global climate change is CO2 emissions. It is generally accepted that the world needs to rapidly curb CO2 emissions to prevent the worst impacts of climate change (Hansen et al., 2013). To overcome this burring issue the whole world is focused on sustainable development, which focused on developing renewable electricity sources such as wind and solar power; systems for join in renewable power into the grid; hybrid and electric vehicles; and energy-efficient lights, motors, appliances, and heating and cooling system to control the impact of CO2 emissions and archived the goal of sustainable development (Peng, Liu & Jiang, 2012). Outdoor energy consumption by lighting is often high due to the long operating hours and high wattage necessary for traffic visibility and public safety. Research reveals that street lighting consumption is 60% of total electricity consumption by a municipality (Shahzad et al., 2018). To achieve a long-standing sustainable and cost-effective outdoor lighting system, such lighting systems can be replaced by Li-Fi lighting technology.

Li-Fi has a low consumption of electricity and it can be integrated into the technology of energy-saving LED lighting, it plays a vital role in sustainability (Montoya et al., 2017). While LED lightbulbs are more expensive than standard lightbulbs, using LED lighting has a positive impact on the energy saving. The Li-Fi uses the visible light spectrum in place of the radio spectrum to transfer data wirelessly *via* the illumination of LED lamps. The key benefit of this technology is to give wireless connections with high data rates.

The Li-Fi positively impacts sustainability, environment, and cost-effectiveness. While the speed of Li-Fi is possibly going to be the true demand for most users, it is amazing that this developing technology of Li-Fi does not harm the environment because it utilizes LED lights. Some of the key impacts of Li-Fi (Manral & Singh, 2016):

1. The longer period of life: Li-Fi LED lights to last up to six times longer than most lights installed in the outdoor area. This eliminates the need for periodic substitutions.
2. Fewer lights needed: With LED lighting, fewer lights are needed for light delivery to be of high quality (Maksimovic, 2018).
3. Sustainability: Lower expense and better value:. The process of Li-Fi technology would save costs, as it will do without electrical equipment such as routers, modems, signal repeaters, wave amplifiers, and antennas in homes and, above all, workplaces (MacKay, 2008). These appliances, which are already connected to the energy grid 24/7, will avoid using electricity and replacing the purpose of the electricity grid with an LED bulb, which is still on during operating hours in most situations, so it would not be an added expense.

In addition, Li-Fi can be considered as a suggested solution to Electromagnetic interference (EMI) sensitive environments such as: Aircraft and hospitals. EMI may cause deterioration or even failure of avionic equipment proficiency. EMI may influence cockpit radios and radar signals, obstructing with pilot and control tower transmission. Laptop computers, mobile phones, and electronic appliances are airborne gadgets that can cause interfering, and these have been accused of causing incidents such as autopilot disconnections, errant flight deck indications, and aircraft turning off (Lusk & Monday, 2017). Moreover, Li-Fi can be safely used in the hospital environments. This technology does not interfere with radio frequency devices which remove the EMI issues that can be caused by using Wi-Fi in hospitals.

Li-Fi provides security against attacks, and device protection is improved since it is important to be in close communication with the LED light beam emitter. It is possible to interconnect only systems by the same light bulb with each other, preventing threats or unwanted attempts to access devices outside our light spectrum. Therefore, Li-Fi is recommended to be used in environmentally-sensitive areas (Kizza, 2005).

### Achieving smart sustainable cities

Recently, it can be seen that the huge attention is giving to the development of smart sustainable cities from research communities, governments, and companies. According to the International Telecommunications Union (ITU) the smart sustainable city can be defined as a city that used information and communication technologies (ICTs) to improve urban services in a way that meets the economic, social and environmental aspects (Aswin et al., 2016). The using of Internet of Things (IoT) technology is considered as an emerging ICTs approach to develop smart sustainable cities (Jägerbrand, 2015). The proposed solution in this paper, which called Lumicomm, utilizes the existing infrastructure of LEDs lighting to develop an IoT system that deliver and collect the content in the city which can be advertisement, news, emergence cases, *etc.* The effect of this system can be discussed according to different aspects of sustainability which includes economic, social and environmental aspects. The impact of the proposed system toward designing a smart sustainable city will be discussed in this section according to these three pitfalls economic, environmental, and social (Bibri, 2017; Saiu, 2017).

### 1) Economic

Lumicomm utilize the existing LED bulbs in communication which can be considered as economical solutions instead of establishing wireless access points. Moreover, the wireless communication that use radio frequency (RF) has limited spectrum, and with advanced technologies that used for smart cities there is a need to solve this issue. The visible light spectrum that used for Li-Fi technology has 10,000 times larger spectrum than the spectrum of radio frequency (Khan, 2017). This can participate mainly to provide economical solution that does not required expensive infrastructure and provide a scalable solution. The using of LEDs are more economical than other products such as fluorescent lighting, since it have long lifespan. The cost of frequent purchasing and changing of the lights will be decreased. In addition, the LEDs consume less energy which reduce the electricity consumption (Campbell, 2016). Therefore, the cost of the smart city projects to deliver content will cheaper than using the Wi-Fi for the same purposes. Also, the proposed system can be groundwork for upcoming smart city applications which reduce the cost of these future applications.

### 2) Environmental

The proposed system also can be used in sensitive environment such as oil platforms or petrol stations that can be affected by electromagnetic radiation. Also, using Li-Fi technology in our system can mitigate the energy consumption which participate mainly to reduce Co2 emission and air pollution. Moreover, the long lifespan of LEDs can reduce the number of used lights which can reduce waste and save environment (Campbell, 2016). Furthermore, the data that collected from users can focus on the environmental problems. The lamps can attached with different sensors that measure air pollution, water level, Co2 emissions, *etc* and utilize the Li-Fi technology for transmission the data. These big data can be collected for each area according to the locations of street lamps. Then the required analysis and processing can be done to produce valuable insights to the decision makers. Therefore, such project can improve the quality of environmental monitoring systems. Furthermore, Lumicomm can be used to send warning or controlling the streetlights to change their colors in emergency situations such natural disasters.

The using of Li-Fi technology in this project can reduce energy consumption, reserve resources and protects the environment. However, there is an issue related to LED technology and the light pollution. The experts have discussed the negative ecological effects of bright blue-rich LED lighting. Therefore, the design principles of LED lighting should be controllable and color-changeable to design lighting in more sustainable ways. This can be a challenge in the field of lighting design (Bonomolo et al., 2017; Tajudeen et al., 2020; Schulte-Römer et al., 2019).

### 3) Social

Lumicomm can be used to provide different services to citizens according to their locations using the LEDs lights. It provides two-way communications and add different services to citizens. The street lights are transformed to become distributed hotspots that work as light sources and provide high speed data transmission. There are different use cases can use the proposed system to deliver or collect content utilizing LEDs lamps. The government can use these lights to broadcast news, awareness campaigns, warnings, traffic monitoring, surveys to evaluate services in specific areas. Also, the companies can utilize the street lights to send advertisements that target specific area and time. Moreover, the users can utilize these lights to upload their complaints, emergency cases, opinions, feedbacks that related to their locations. The collection and analysis of the data in different areas of the city can participate in improving the decision making. All of these uses of the system can improve the quality of life and increase citizen satisfaction and access different social services.

## Related Works

The related works can be categorized into research studies, companies that work on the development of Li-Fi technology, and the systems that have been developed for the Li-Fi technology. It should be mentioned that the related works were presented and discussed in details in our previous research (Albraheem et al., 2018). This research (Albraheem et al., 2018) provided a comprehensive survey about Li-Fi technology and the differences between this technology and similar one. It covered the background information required to understand Li-Fi, the significant research in the Li-Fi field , and it discussed the Li-Fi industrial trends over the recent years including Li-Fi compatible commercial products. In order to reveal the limitations and issues that require improvements in the previous studies, a comparison between multiple Li-Fi based systems has been conducted. According this comparison that presented in the previous study (Albraheem et al., 2018), most of the systems are used for navigation and customer guidance inside buildings. Furthermore, it is noticed that all of the systems lack an important feature as they do not give the user the ability to contribute in generating content and view other users’ content. The users’ content that related to their locations can be opinions, complaints, emergency messages, *etc.* The content of the systems which are news, advertisements, awareness messages is generated and managed by system administrators. This shows a limitation in which end users cannot participate in the process of content generation, and therefore creates a gap that needs to be filled to utilize huge contextual data collected from end users. Providing the feature of content contribution by users allows system owners to collect huge contextualized data for analysis and improvement purposes. This became a motivation to propose a Li-Fi-based IoT framework that utilizes light sources integrated with the Li-Fi technology to collect huge amounts of geo-contextualized data for further analysis and processing. This framework can be presented as a hierarchical architectures composing multiple tiers: collection and aggregation of the users’ data, processing, visualizing and analyzing huge data which can improve decision making.

Furthermore, this section will cover the latest technologies and architectures that can be adopted to implement smart public lighting infrastructures. There are different technologies and architecture that are adopted to implement smart public lighting infrastructures. According to Leccese, Cagnetti & Trinca (2014), the paradigm of the smart city helps in renovating the traditional city concept. The architecture of the smart city application has hierarchical layers structure that are compose of local control cards, sensors, as well as actuators. The architecture consists of three layers that include remote control, apparata and local control. The apparata layer deals with the hardware and software that are designed for controlling single apparatus. It facilitates communication with the higher layer through a ZigBee mesh wireless network. ZigBee entails a standard for wireless communication using the IEEE 802.15.4 protocol layer. It has a higher consumption of power and fewer devices, which make it efficient to build sensor networks in instances where applications require low power consumption. In the local control unit, data obtained from single units are collected to check how they function. In this unit, a RaspBerry-Pi control is used for collecting and locally elaborating data from single apparatus to verify that problems do not exist in the system. In case there is a problem, the card will send an error message through the internet using the upper layer. Moreover, this paradigm used Worldwide Interoperability for Microwave Access (WiMAX), which encompasses the radio communication technology that is used in connecting the local control unit and the World Wide Web. The technology uses the IEEE 802.16 protocol with the aim of delivering internet connectivity to regions in which normal DSL service might be challenging and costly to install (Leccese, Cagnetti & Trinca, 2014). WiMAX is chosen because in some areas of the city, telecommunication lines have not adequately penetrated.

Ideally, the smart city infrastructure has the smart pole that entails a street light lamp majorly comprising of local control device, computational unit used to process video, communication devices, video camera, as well as weather sensors (Gagliardi et al., 2020). The smart poles are capable of exchanging data among themselves. There are two types of smart poles in this study which are smart master pole and smart slave pole. The master one is equipped with motion detection, weather station and dimming devices, while the slave pole is equipped with dimming devices only. The communication technology that used to connect smart ploes together is IEEE ZigBee wireless, while the 3G/4G/WiFi communication network are used to connect poles with remote server. On one side, in case vehicles or pedestrians are not present, light intensity of the LED will be kept at a minimum value that the street lighting regulations prescribe. On the other side, if there are vehicles or pedestrians, the light intensity will be increased to achieve the most appropriate value in the monitored area. Additionally, there is the working mode of failsafe default emergency condition. In situations where connectivity is lost or a component has been damaged, a default condition will be activated to prevent general safety from being compromised (Gagliardi et al., 2020). In a similar study (Leccese, 2013), ZigBee-based wireless devices consist of presence sensor, light sensor, operating control, emergency device and control unit. The presence sensor identifies the passage of any pedestrian or vehicle, which enables it to give an input to turn on a lamp. Based on this feature, lamps are only switched on when necessary, thereby preventing wastage of energy. The light sensor measures the brightness of sunlight and gives information. Operating control improves management of fault and maintenance of the system. Through this sensor, the system can recognize false positives since it will compare identified parameters with the stored data. The emergency device is used when there is an emergency and it excludes the overall sensor system as it seeks to immediately turn on the lamp. The control unit runs the software for analysing the system after the sensors transfer the information acquired.

Importantly, Shahzad et al. (2016) suggests that outdoor lighting can be addressed through smart control of public lighting that would save energy and improve efficiency. Smart lighting that uses electronically controlled LED lights for adaptable illumination and monitoring ensures that the system is energy efficient. Smart LED lights also have scalable wireless network that offers reduced cost, increased user satisfaction and improved reliability (Shahzad et al., 2016). Conversely, Carli & Dotoli (2020) confirms that it is crucial to have an efficient decision-making procedure, which would support the city energy manager to determine the optimal plan for energy retrofit of a public street lighting system. The decision model should maximize the reduction of energy consumption, while realizing optimal allocation of the retrofit actions within the subsystems of street lighting. As a result, the available budget will be efficiently used (Carli & Dotoli, 2020).

According to Elejoste et al. (2013), sensing capability should be provided to the end users to acquire environmental information to help reduce the consumption of energy. Some environmental factors can be measured in specific areas such as the level of pollution and concentration of gases. As the sensing capabilities are being deployed, it is crucial to balance between how reliable the environmental measurements are and costs required for the end nodes. There would also be the need to detect the movement of pedestrians and vehicles. Based on the nature of what needs to be detected, the technology that would be implemented tends to vary. For instance, in the detection of pedestrians, it will be adequate to use simple passive infrared sensors (PIRs) (Elejoste et al., 2013). According to Lee & Huang (2015), WSN-enabled streetlights have various applications. Due to its ability to transfer data from the streetlights to the central server, the wireless sensor networks can help determine road traffic, as well as adjust the intensity of lighting to save energy in case traffic reduces. Furthermore, a panic button can be installed on the streetlights and this will enhance ease of reporting of traffic accidents and emergencies. Despite having designed and tested WSN-based streetlights for a long period, the cost incurred to modify conventional streetlights is very high. Replacing such streetlights with new and intelligent streetlights is even more costly (Lee & Huang, 2015). In similar study (Lau et al., 2015), a traffic-aware lighting scheme management network (TALiSMaN) is presented. The network progressively adjusts the illuminance of streetlights depending on the needs of different road users, while improving their energy efficiency by ensuring that energy use is minimized. Moreover, TALiSMaN helps detect road users and share the data with streetlights in the nearby area. After the information is received, optimum conditions of lighting that meet the needs of road users would be ensured. It also ensures that the road is not illuminated at higher levels since this would waste energy.

On the other hand, Bonomolo et al. (2017) states that lighting systems account for about 19% of the total electric energy consumption across the globe. As a result, when the retrofit action is used as a strategy for lighting system, substantial energy savings is achieved. In most nations, approximately 75% of the lighting installations are older than 25 years hence considered outdated. Experts need to undertake a range of activities to help improve the process of lighting refurbishment. These activities include developing a sound and clear overview of the market of lighting retrofit, planning of trigger discussions, initiating revision and enhancing local and national regulations, increasing daylight robustness, and understanding the processes of lighting retrofit through the provision of enough tools for different stakeholders. Other activities are certifications and loan programs and demonstrating the state-of-the-art lighting retrofits (Bonomolo et al., 2017). In addition, (Beccali et al., 2019) affirms that the retrofit of urban lighting systems can help improve the quality of light. Factors such as the lifestyle, habits and expectations of users may contribute to the lighting systems. For instance, these factors can help decide the most relevant control techniques and quality of light. The variables can also affect the overall performance of the system (Beccali et al., 2019).

## Materials & Methods

### System architecture

**Figure 2:**
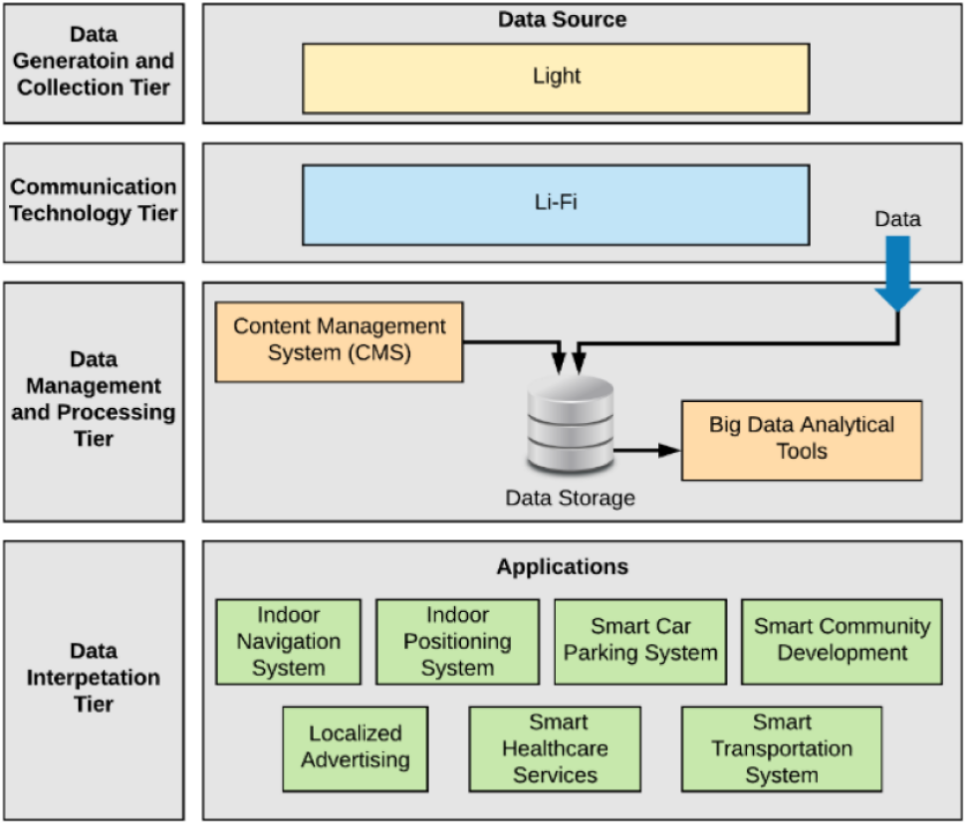
Proposed Li-Fi-based IoT hierarchal architecture (Albraheem et al., 2018).

In previous study (Albraheem et al., 2018) as shown in Fig. 2 a Li-Fi-Based Hierarchical IoT Architecture was proposed based on the idea of generating data for analysis by deploying Li-Fi components in multiple indoor and outdoor environments. The source of data dissemination and data collection is the Li-Fi light. The collected data then is analyzed and processed to build decisions based on the result. The proposed architecture comprises four tiers: (1) data generation and collection, (2) communication technology, (3) data management and processing, and (4) data interpretation. Figure 2 illustrates the proposed layered architecture (Aswin et al., 2016). This paper considers the first three tiers of the architecture, as it focuses on the development of a generic geo-Li-Fi system that comprises the following components to build a complete Li-Fi-compatible system.

**Figure 3:**
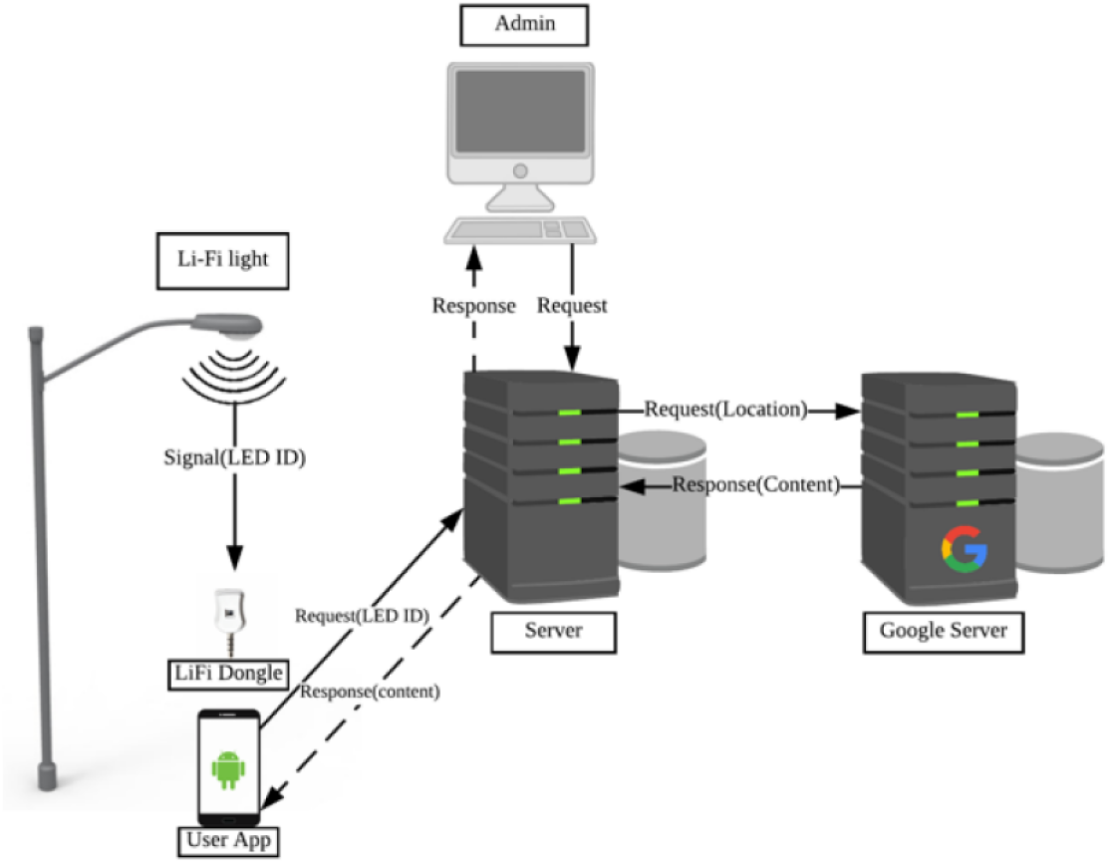
Architecture of the proposed system.

The general idea of the system is utilizing light sources by empowering them with the Li-Fi technology to act as a mean of data generation and collection in different urban areas within a smart city. As shown in Fig. 3, The system uses geo-Li-Fi technology consisting of LEDs and Li-Fi compatible data receivers. The system is based on the idea of allowing both end-users and system administrators contribute in generating content. The system administrator log into a web-based Content Management System CMS to create and manage Li-Fi regions within a smart city. A city can be divided into multiple regions, whether indoor (inside buildings) or outdoor with various sizes. By creating a region, the administrator can control Li-Fi LEDs and their associated content. The concept of regions is proposed to allow maximum flexibility in managing a group of related LEDs. The administrator can group multiple LEDs under one region such as a parking lot or a university campus. The grouping provides the admin with the possibility of disseminating the same content for all LEDs within the region. The content associated with each LED can be anything such as news, advertisements, emergency notification, etc. When the user is within the defined Li-Fi range of the LED lamp, the content is received by the light sensor components and forwarded to the mobile device. Once the content is displayed to the user, the user is allowed to view, upload and save LED.

**Figure 4:**
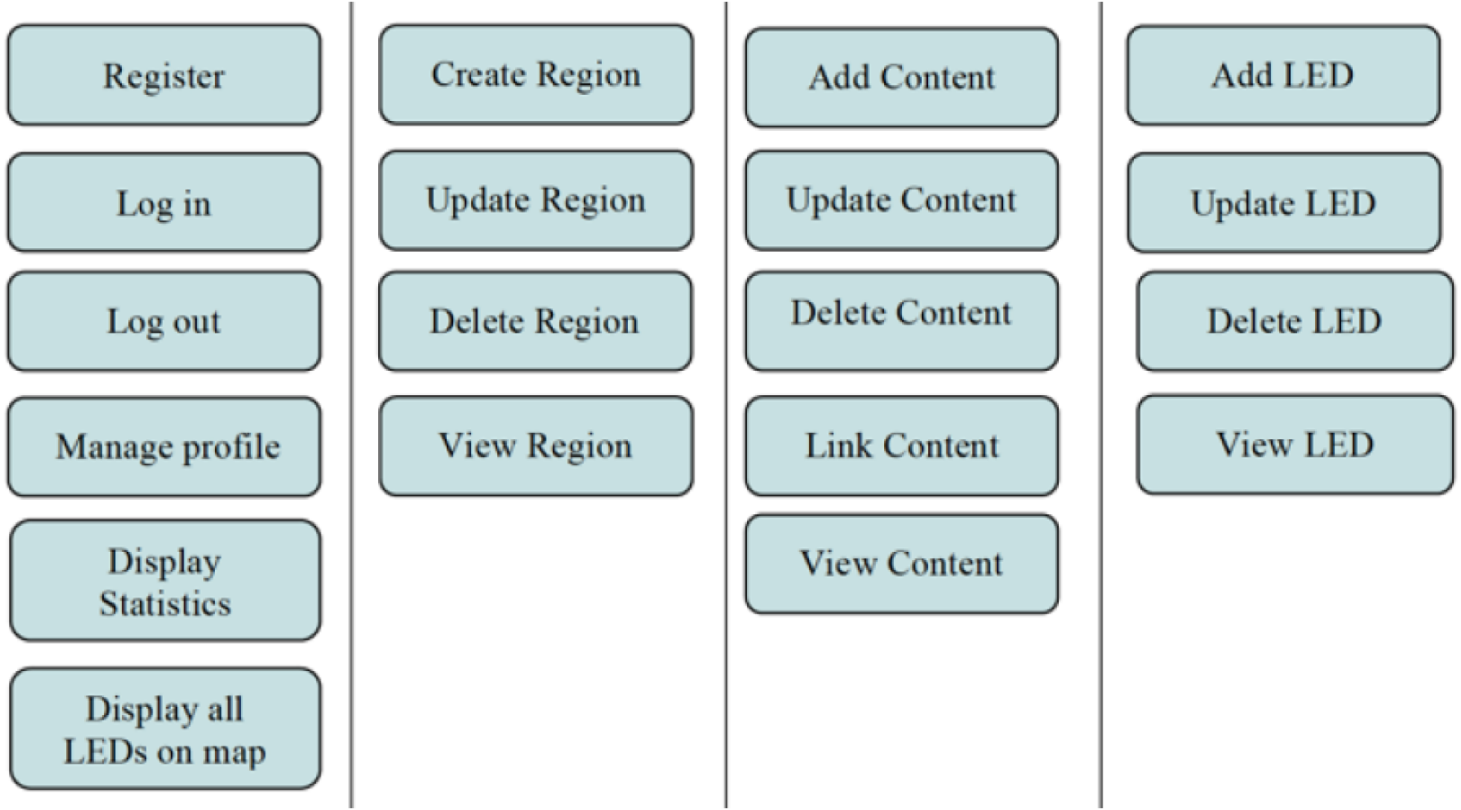
CMS functions.

The proposed system as shown in Fig. 4 has four components:

(1) Content Management System (CMS): is a web-based solution designed to allow administrators fully control and manage LEDs and content of the system. In addition, administrators have the ability to manage LEDs by regions. Grouping LEDs by region give the admin the possibility of spreading the same content to all LEDs under his administration, allowing more scalability. The main functions of the CMS is shown in Fig. 4.

Managing system’s content and creating Li-Fi regions is performed through the CMS subsystem. It provides three main functions to the admin: (i) Region Management, (ii) LEDs Management, (iii) Content Management. This subsystem plays an important role in the virtualization of physical Li-Fi regions. Through the CMS, the admin is able to map actual Li-Fi light-lamps locations into logical locations grouped in the same zone (*i.e.,* region). This logical grouping provides more scalability when managing the grouped LEDs. To use the system, the admin needs to login using a username and password. The admin is required to create a region before inserting LEDs records in the system. The region’s information includes the city where it is located along with the delimiters. Afterward, the admin will be able to insert multiple LEDs records to the created region. Inserting a LED requires the admin to specify a unique identifier hardcoded in the geoLi-Fi chip integrated with the LED. In addition, the web client will communicate with Google Map API to allow the admin pinpointing the logical location of the LED and store it in the database. Unlike managing LEDs, managing the content is an independent process in the system. This allows the admin to manage huge amount of data collected from the Li-Fi sensors or content created through the CMS. To add content, the admin is asked to specify its category. The category is used for further classification and ease of analysis. Afterward, the admin has the option of either linking the content with one or more LEDs or leave it unlinked.

**Figure 5:**
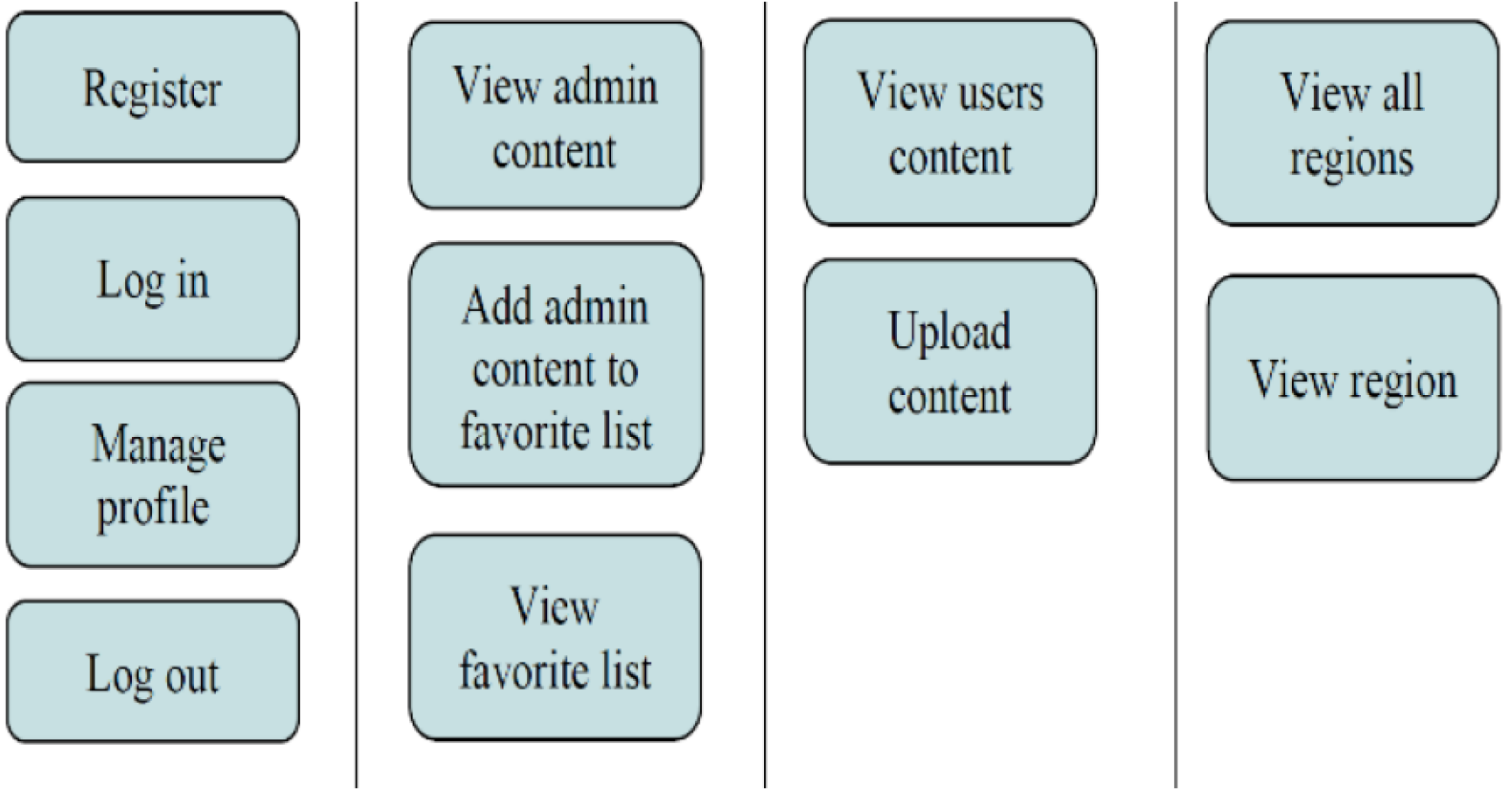
Mobile-based application functions.

(2) Location Aware Mobile-based application: is a compatible Li-Fi mobile application, designed and implemented for regular users to interact with the technology by uploading, viewing and saving location-related content. The application provide the services to user to interact with the CMS to upload contextual data and also to receive LBS services. The main functions is shown in Fig. 5.

(3) Data transmitters–The LEDs: are responsible of transferring data to compatible devices. Encoding data into light for transmission is achieved by varying the light intensity of the LED. The variation of light intensity causes the LED to flicker ON and OFF at a very high speed (Manju, Jerusha & Banumathi, 2015). Those flickers are the representation of the data being transmitted. When the LED is switched ON it logically represents a transmission of a “1”, when it is switched OFF it is the representation of a “0”. The combination of 1’s and 0’s generates different data strings. The LEDs bulbs used in the proposed system are equipped with a GeoLi-Fi transceiver chip. The GeoLi-Fi transceiver chip transforms lighting into a geolocation system having high energy efficiency.

(4) Data receivers: The Li-Fi signals produced by the LED can be detected using a separate receiver called Li-Fi dongle or using basic mobile phone’s camera. In case of Li-Fi dongles, receiving data is the duty of the photo-detector within. Once the mobile device enters the range of the LED light, the light sensor detects the signal and converts the changes in its intensity to electrical signal. The mobile application afterwards is triggered to send a request to the server, retrieving the content associated with the current LED. Consequentiality, the application displays the content to the user along with a list of recommended nearby places retrieved from google maps API.

### Implementation and development environment

This section consists of the hardware and software tools used to develop the system. The hardware section presents a list of used hardware in addition to a thorough description of the Li-Fi kit that has been used in the project. As in the software section, the required software development kits SDKs are mentioned.

#### Hardware elements

**Figure 6:**
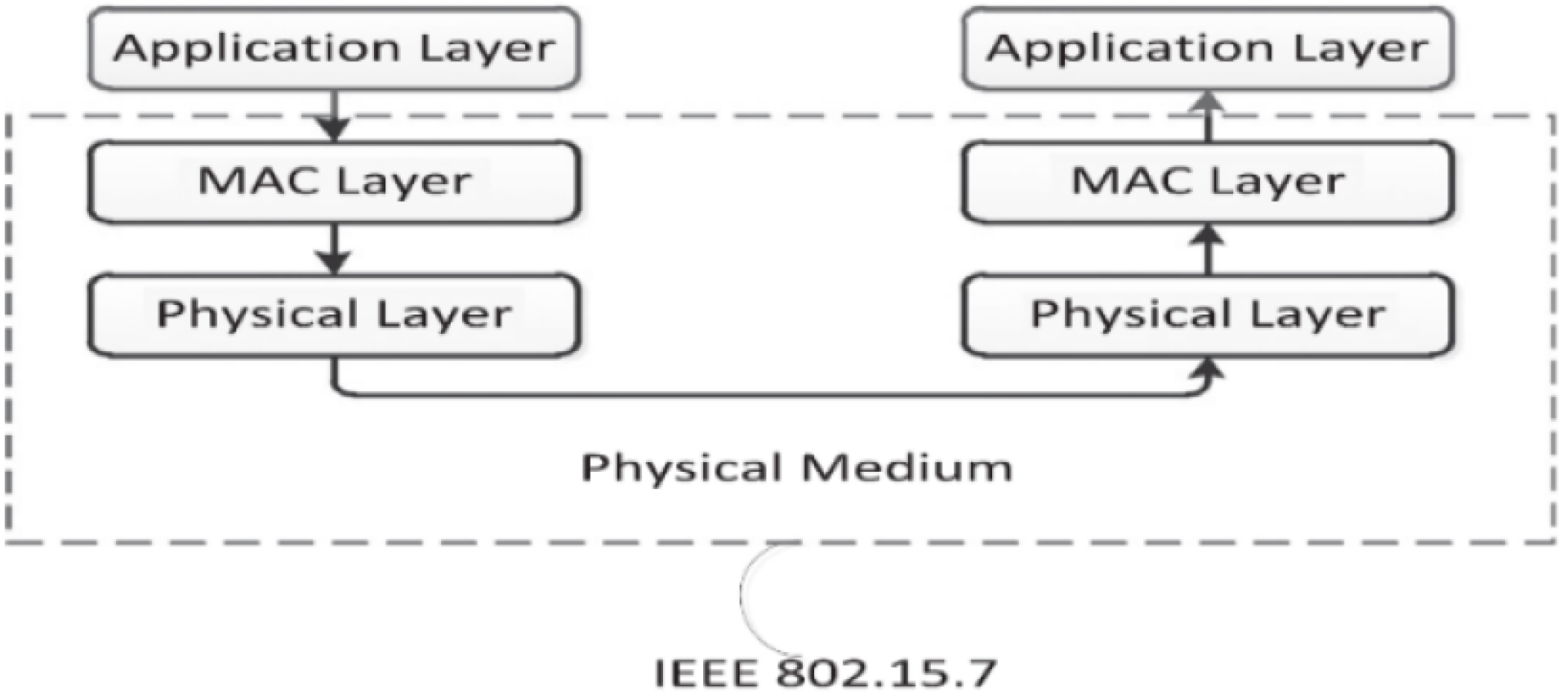
Layered architecture of VLC (Khan, 2016: CC BY-NC-ND 4.0).

Basic Li-Fi system relies on two essential optoelectronic components: the LED as transmitter and the Li-Fi receiver. The hardware used to implement the proposed system is constituted by Oldecomm company, a company specialized in manufacturing Li-Fi products (Oledcomm, 2017). The main components that used for the proposed system are the GeoLi-Fi emitters and receivers. They generally consist of three layers which are: physical layer, Mac layer and application layer as shown in Fig. 6 (Schmid, Corbellini & Mangold, 2013). The transmitters/emitters are equipped with the GeoLi-Fi transceiver chip which works in compliance with IEEE 802.15.7 communication standard for visible light communications as well as to European Union norms and directives (Oledcomm, 2017a; Khan, 2016). The emitters encode the data stream into the light by varying the current of the LED.

**Figure 7:**
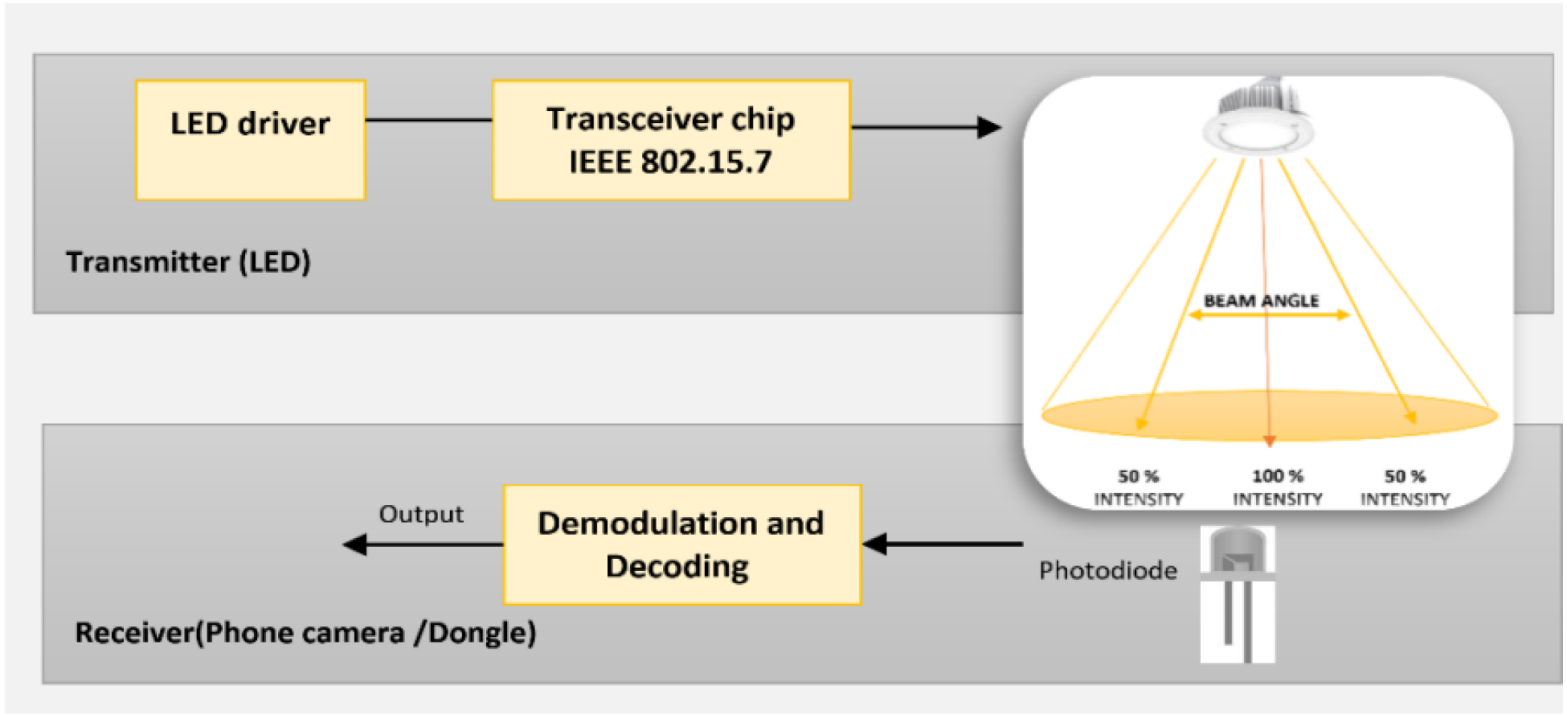
Li-Fi hardware architecture.

In developing the proposed system as shown in Fig. 7, we have experimented the two types of modulations to detect the modulated Li-Fi signals. Each modulation required its own hardware components. In the first modulation approach, the dongle acts as a Li-Fi signal receiver. The photodetector equipped intercepts and decodes the Li-Fi signal. The second modulation approach relies completely on smart phones’ camera. In this approach, different modulation algorithms must be considered to encode the signal in the optical wave. Moreover, the LED flickering rate must be fixed to a rate that is suitable for regular cameras to detect (image sensor frame rate in cameras) (Chow, Chen & Chen, 2015).

**Figure 8:**
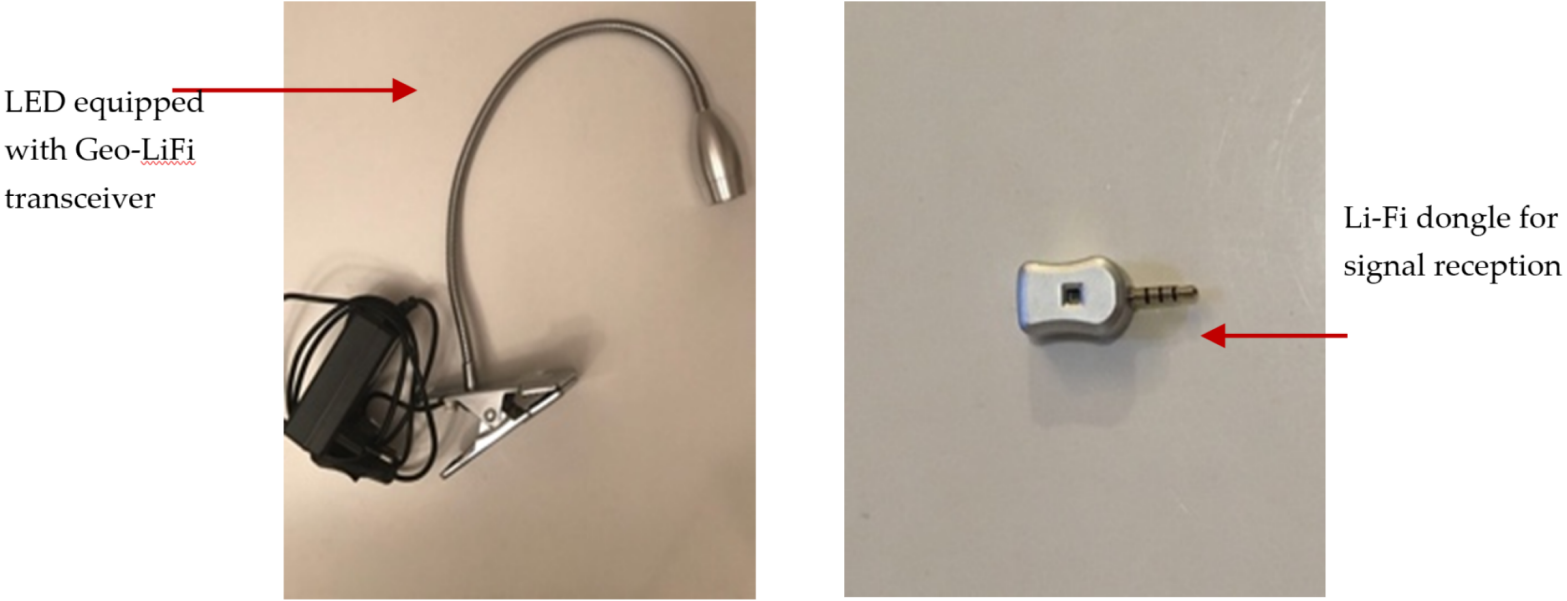
GeoLi-Fi development kit (dongle as receptor).

There are two types of signal receptor that used which are Li-Fi dongle and smart phone camera. The GeoLi-Fi development kit provided by Oledcomm company was used. The LED transmitters are equipped with the GeoLi-Fi transceiver chip and have been designed to work with indoor and outdoor LED based illumination. The kit consists of small pieces of hardware that connects to mobile phones which called dongles. These dongles are equipped with photodetectors to receive and decode the Li-Fi signal and can be connected to headphone jacks. As it appears in the figures below, the kit used consists of Li-Fi LED lamps and a signal receiver. Figure 8 presents the components used in this modulation scheme.

**Figure 9:**
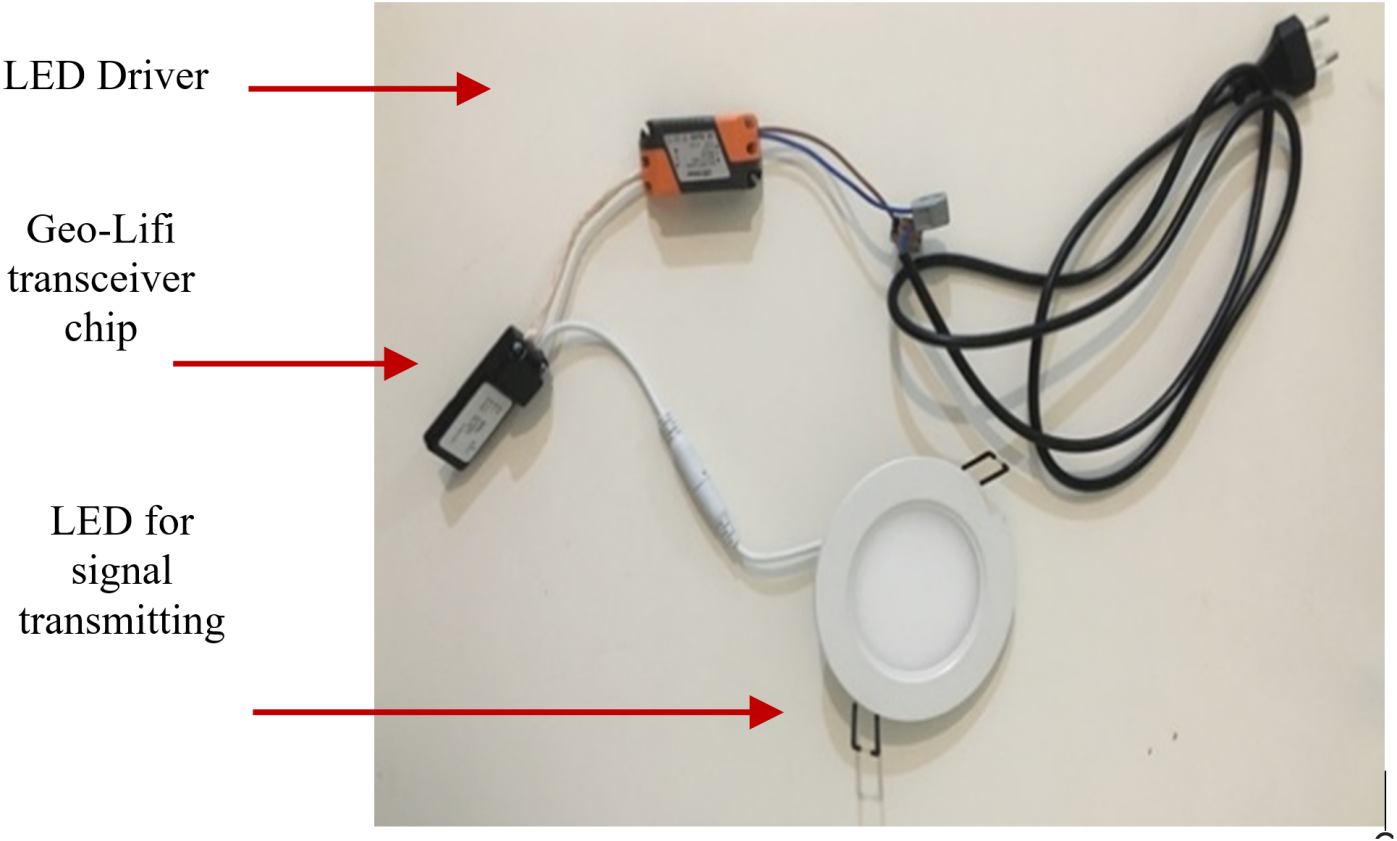
GeoLi-Fi development kit (camera_modulation).

**Figure 10:**
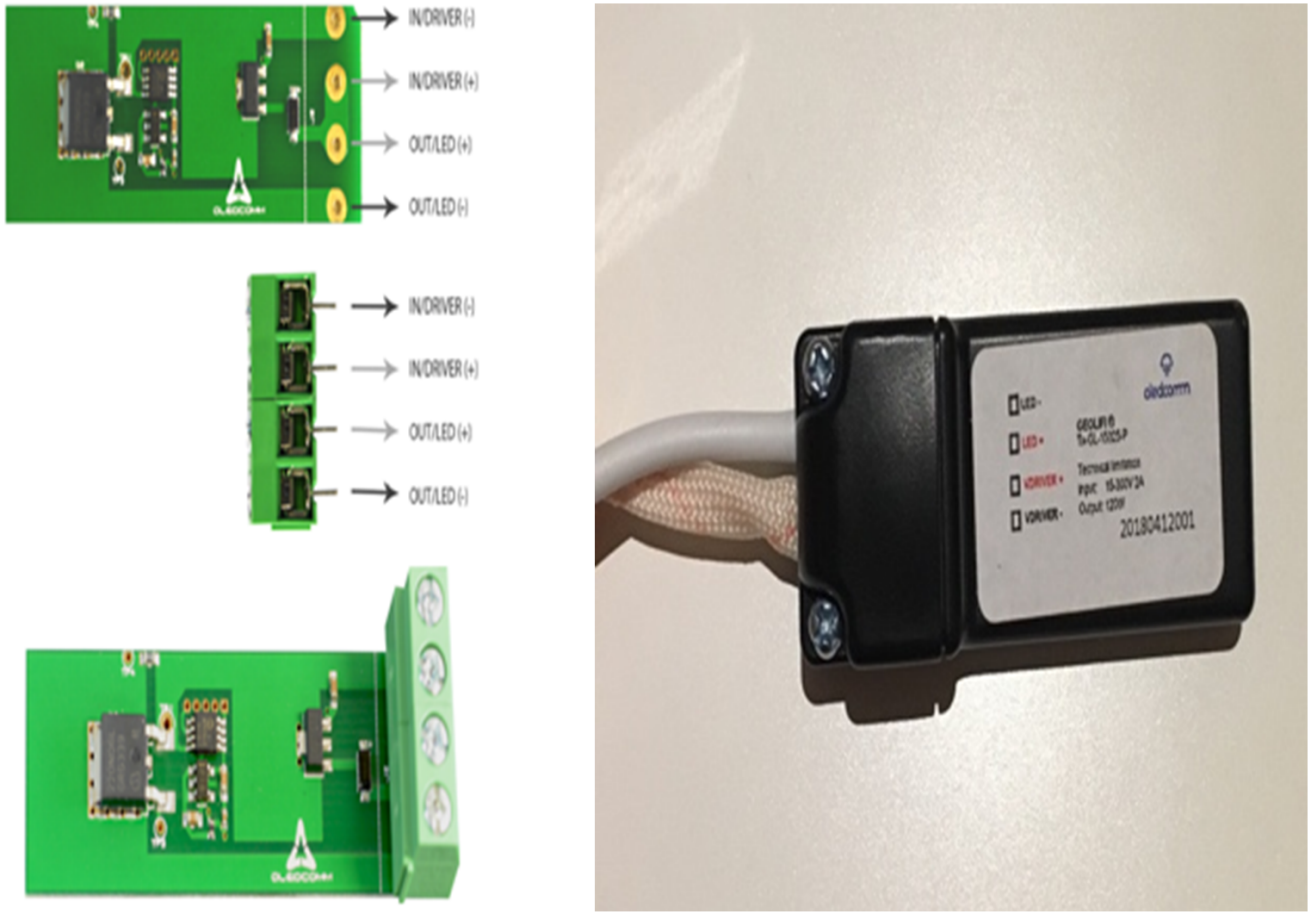
GeoLi-Fi transceiver (camera_modulation).

For the camera receptor, signal reception is fully reliant on the mobile phone camera. The GeoLi-Fi transceiver in this case is connected externally to the LED and an LED driver. However, the transceiver can be integrated with the LED driver housing. Figure 9 represents the hardware used in order to transmit the Li-Fi signal. The transceiver’s outputs are connected directly to the LED and inputs connectors are connected to the LED driver, which is itself connected to the power source to supply the whole system. Figure 10 demonstrates the GeoLi-Fi transceiver structure without the housing. The transceiver can be used with a wide range of LED lightings as long as they meet the chipset specifications.

#### Software system

**Figure 11:**
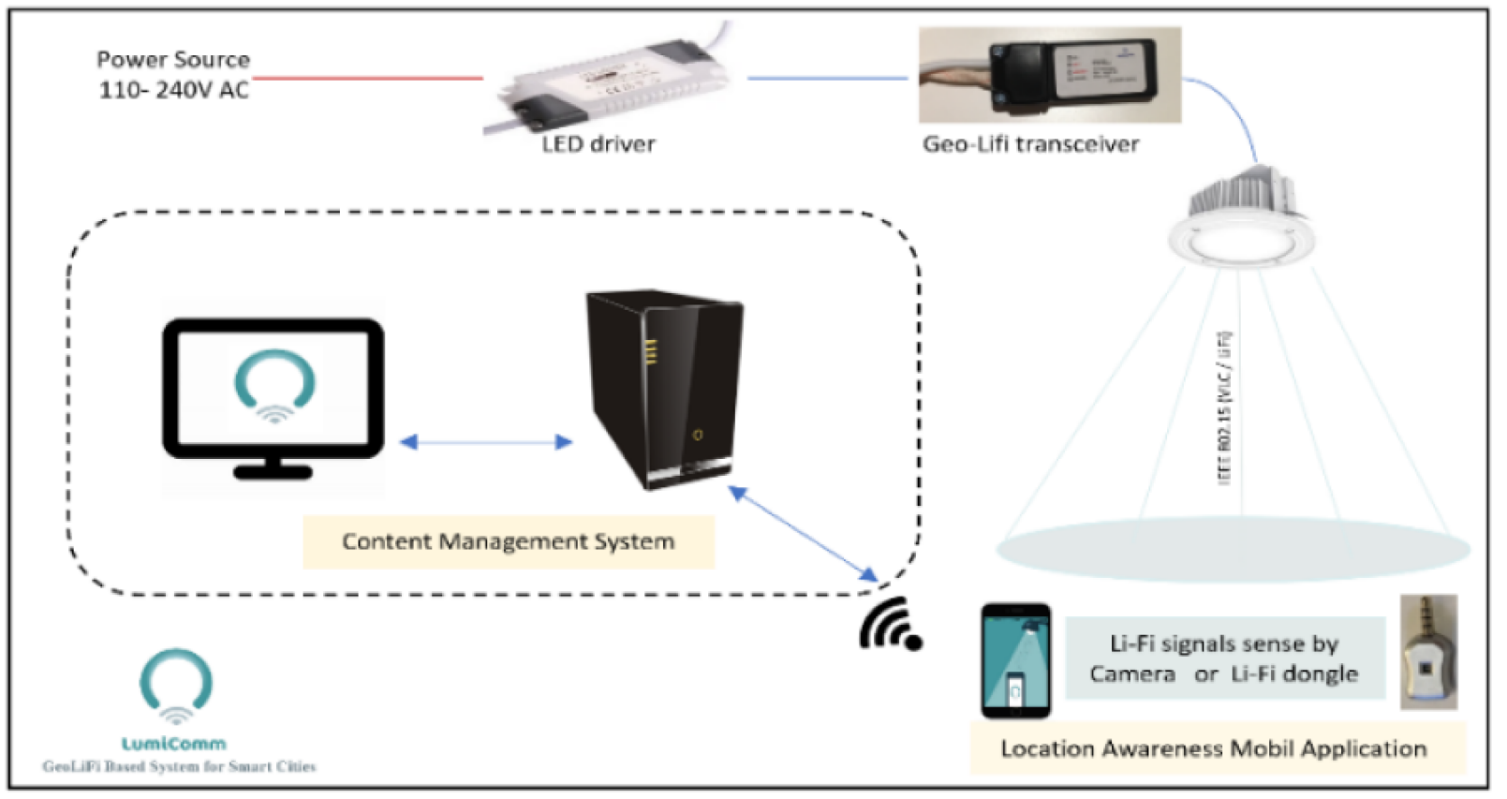
Lumicomm system.

In the matter of implementing the first three tiers of the architecture, a software system has been developed that is composed of two subsystems as shown in Fig. 11: content management system (CMS) and the mobile-based application. The CMS is implemented completely as a web panel using: illustrator, Brakers and Xmap. On the other hand, the mobile application is designed to be compatible with the Li-Fi technology. Therefore, a GeoLi-Fi SDK was used to achieve the compatibility, in addition to Android studio and Xcode. The GeoLi-Fi SDK is comprised of the necessary tools, libraries and APIs to build the applications and process the received Li-Fi signal through the light sensor. The mobile application is developed for Android-based devices, Once the user attaches the Li-Fi receiver—in this case, the Li-Fi dongle—through the audio jack, a permission for RECORD_AUDIO needs to be granted in order to pass the Li-Fi signal. Once the permission has been granted and the user is within the range of the Li-Fi lamplight, the application installed on the user mobile device will send an automatic HTTP request to a web server, and an HTTP response will be returned, containing content associated with the LED. The system was built to be completely compatible with Li-Fi using a Li-Fi dongle, however, in the final stages of the development a new way of receiving Li-Fi signals was released using basic phone’s camera. To keep in pace with the development of the technology, we developed a light mobile application fully compatible with the Li-Fi reliant on phone’s camera. This application is implemented using another SDK that support iOS applications.

### Testbed setup

**Figure 12:**
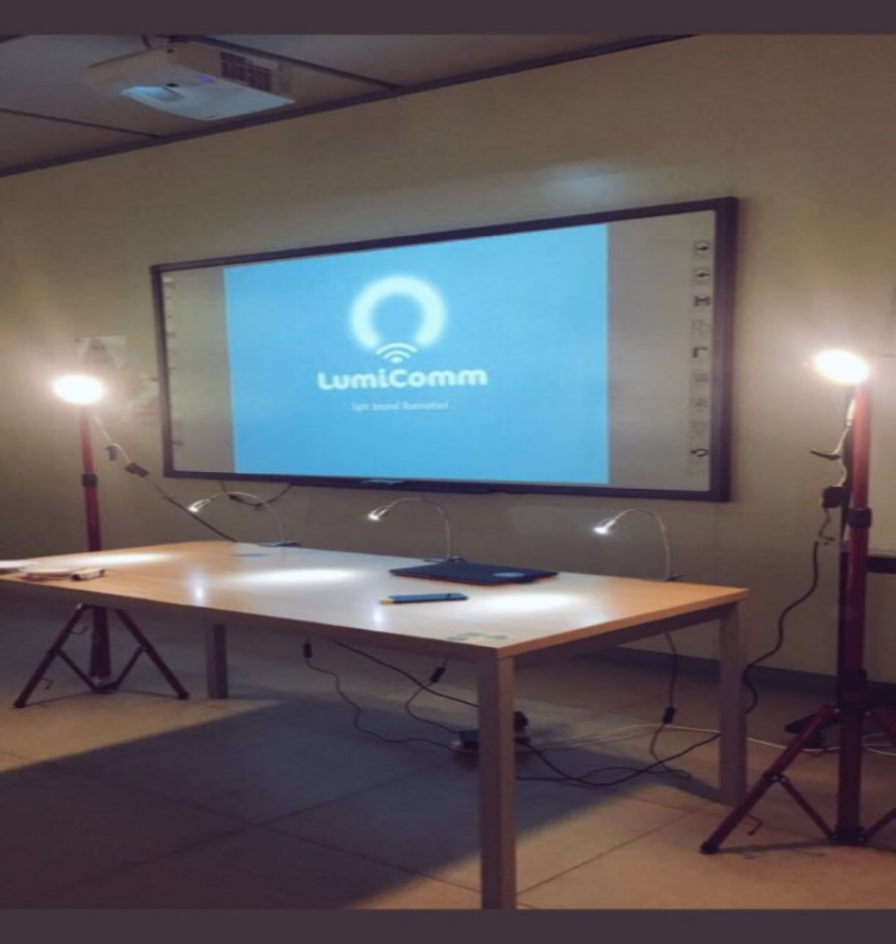
Testbed for the proposed system.

**Figure 13:**
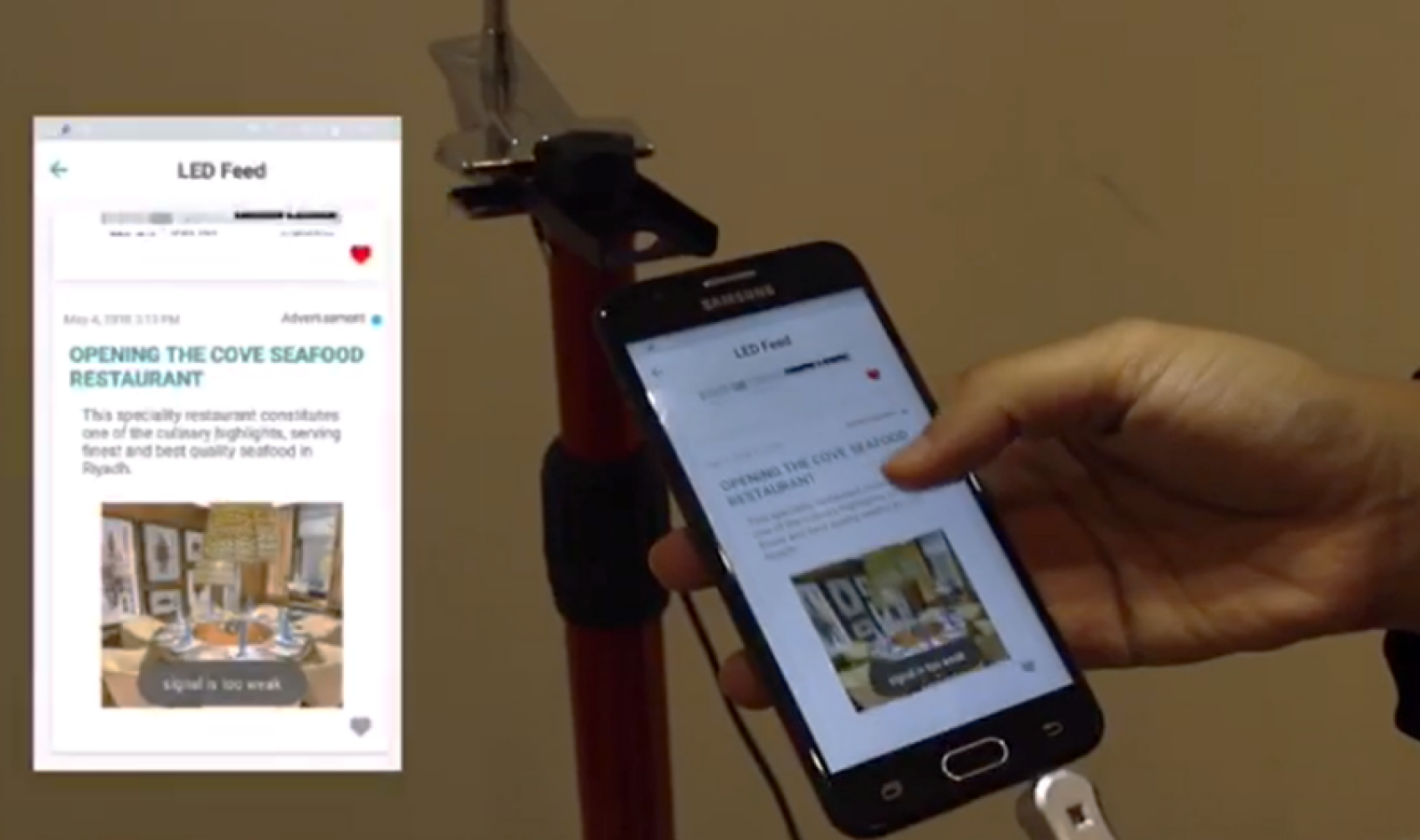
Mobile application (dongle as receptor).

After implementing the GeoLi-Fi system, and preparing the required hardware, the testbed as shown in Figs. 12 and 13 has been used to accurately evaluate the system’s functionality. This testbed is composed of many components which include: three LED lamps that compatible with dongle modulation, two Led lamps that compatible with camera modulation, laptops and different mobile devices. The specifications of the LEDs are described in Table 1. The mobile phones that used are Huawei P9 Plus, Android version 7.0 , Motoral X android level 6.0, Samsung Galaxy S7 android level 6.0 and Samsung Galaxy S6 Android level 5.0.

### Experiment

Lumicomm is a system aiming to transform basic lights into a source of data. As mentioned previously, we already implemented the system to be consist of: a CMS (content management system) that can be used to manage LEDs, content and regions, and a mobile awareness application that can be used by user to interact with the Li-Fi-technology to upload, view and save location-related content. The scenarios where Lumicomm system could be deployed are various. The experiment was performed using the testbed that mentioned above with support of a real world example of King Abdullah Financial District (KAFD) to show the system functionality. To accomplish the goal of transforming KAFD into a Li-Fi-enabled region, the process goes through four phases:

• 1. Planning phase: includes the actual distribution and positioning of the LEDs, in addition to specifying the settings for each LED.
• 2. Installation and deployment phase: this phase concerns the hardware installation in the areas specified in KAFD. In other words, this phase can be considered as the actual installation and creation of the regions.
• 3. Execution phase: the logical installation and creation of the regions. In this phase, the actual locations of LEDs are mapped to the logical location in the system and grouped under the same regions for administrative tasks.
• 4. Testing phase: the region is tested in this case from the second subsystem, which is the mobile application. The testing has been conducted according to different scenarios.

1) Planning phase

**Table 1:** LEDs specifications of the experiment.

**Specifications**	**LED Lamp (camera modulation)**	**LED lamp (dongle modulation)**
Quantity	2	3
Power (W)	14,8 W	3 W
Voltage (V)	234,7	85
Receiver	Phone’s camera	Dongle

**Figure 14:**
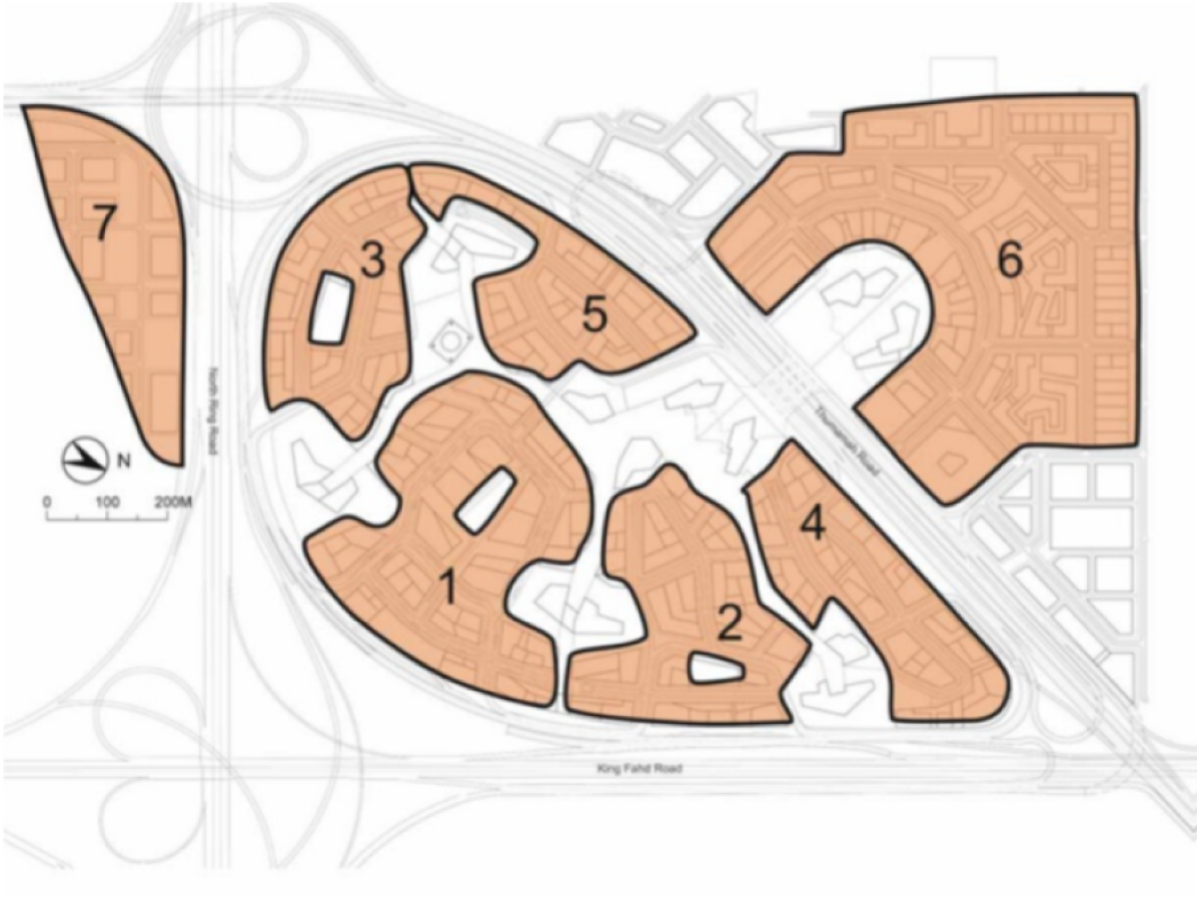
KAFD areas (Wikipedia, 2017; CC BY-SA 3.0).

KAFD is designed to be divided into multiple areas (Wikipedia, 2017) as shown in Fig. 14. In this experiment, each of these areas will be equipped with multiple Li-Fi enabled lighting solutions. Five areas were chosen which were the Financial Area, Residence Area, Attraction Area, Monorail Area and Al-Wadi District Walk. In each of these area, multiple Li-Fi LEDs have to be installed.

To show how the proposed system will represent the scenario, LEDs are distributed among the areas of KAFD region .In each of these area, multiple Li-Fi LEDs have been installed. Those LEDs have been labeled with the area identifier and positioned in optimal locations to cover up most of KAFD subareas. The reason behind this distribution is to provide maximum flexibility in controlling LEDs’ content, as each area is most likely to contain content different than the other when considering the nature of the majority of its users. For instance, the Al-Wadi area may include many advertisements and proximal marketing content, in addition to guidance content for KAFD (maps, notification, announcements *etc.*). In total, KAFD has been equipped with 20 LEDs in the experiment. It should be mentioned here that the CMS of the proposed system give the ability to create regions in the real map then link the LEDs that related to this region. This will be explained later in the execution phase.

2) Installation and deployment phase

In this experiment, as mentioned above the features of CMS (content management system) are shown by using the example of KAFD. Also, the testbed that contains LED lamps equipped with Li-Fi technology are used to get, upload, save user’s content to the system. The specifications of the Lamps that used in this experiment were described previously in Table 1. It should be mentioned here that the lamp that suggested to be used in smart cities are manufactured by Technilum (Technilum company, 2018; Oledcomm, 2017a) company and empowered by Oledcomm GeoLi-Fi transceiver chip as shown in below figure. These lighting solutions belong to the GeoLi-Fi product range and they are compatible with both modulations: Li-Fi dongle and camera modulation. The streetlight comes in two heights 8 m and 4 m (Technilum company, 2018) and it is assumed that the coverage range of the light is 10 m^2^ (Oledcomm, 2017b). To simulate these GeoLi-Fi streetlights, we used also GeoLi-Fi LEDs lamps with different modulations: Li-Fi dongle and camera modulation as described in the proposed testbed.

3) Execution phase

**Figure 15:**
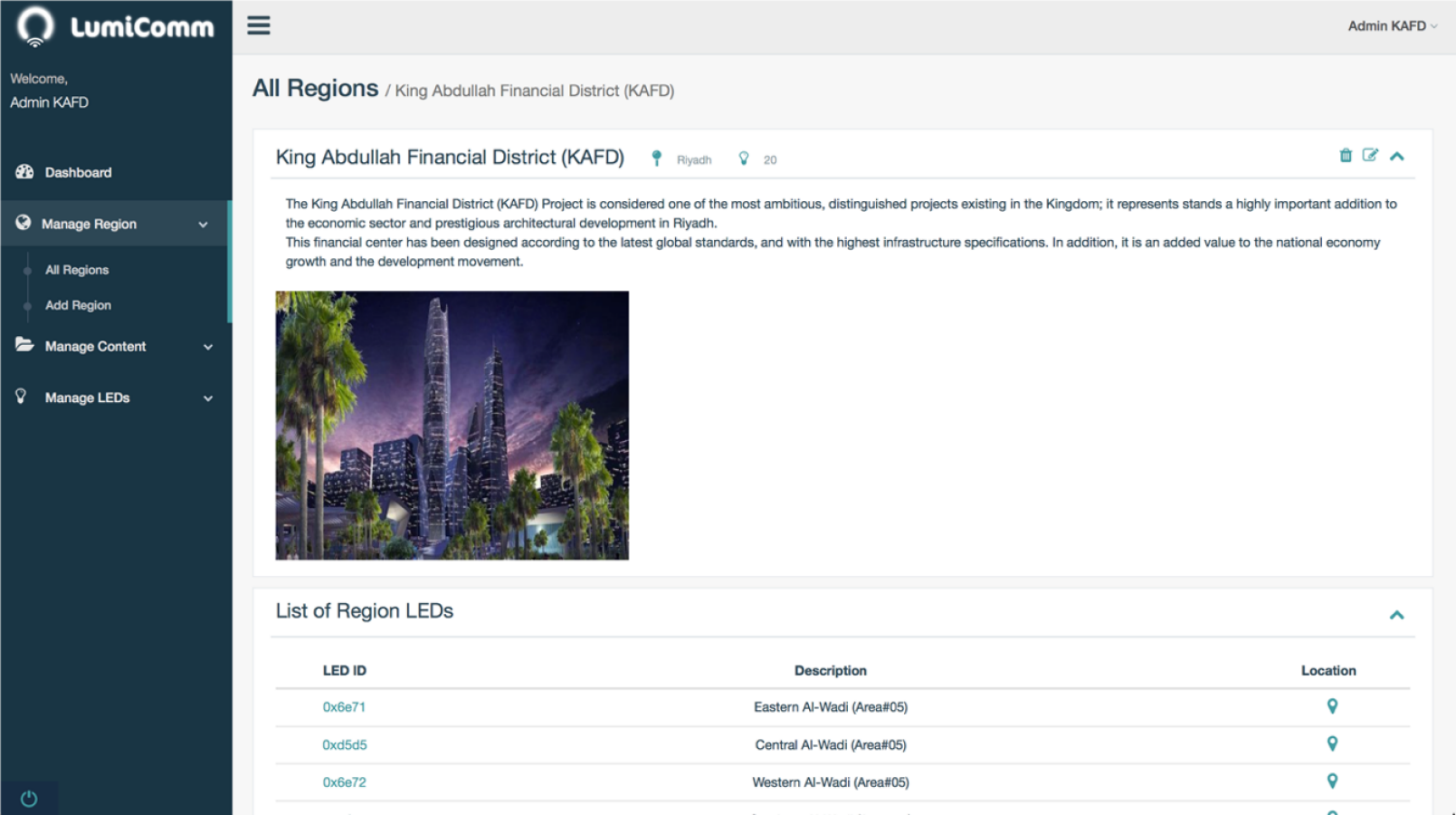
Screenshot of KAFD region in the admin web panel of Lumicomm (CMS of the proposed system).

**Figure 16:**
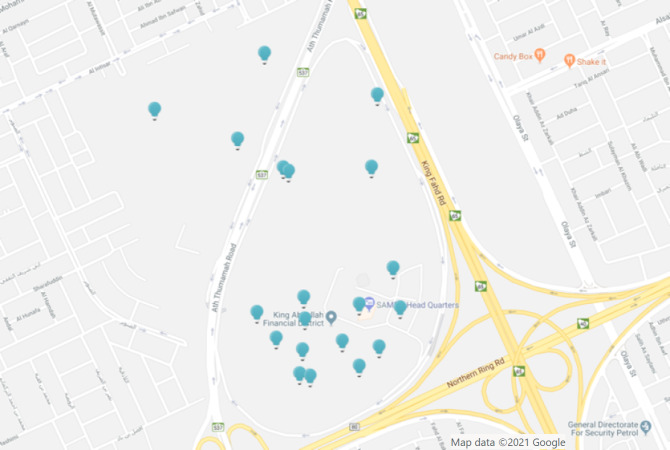
LEDs distribution (map view) admin web panel of Lumicomm (CMS of the proposed system). Map data ©2021 Google.

**Figure 17:**
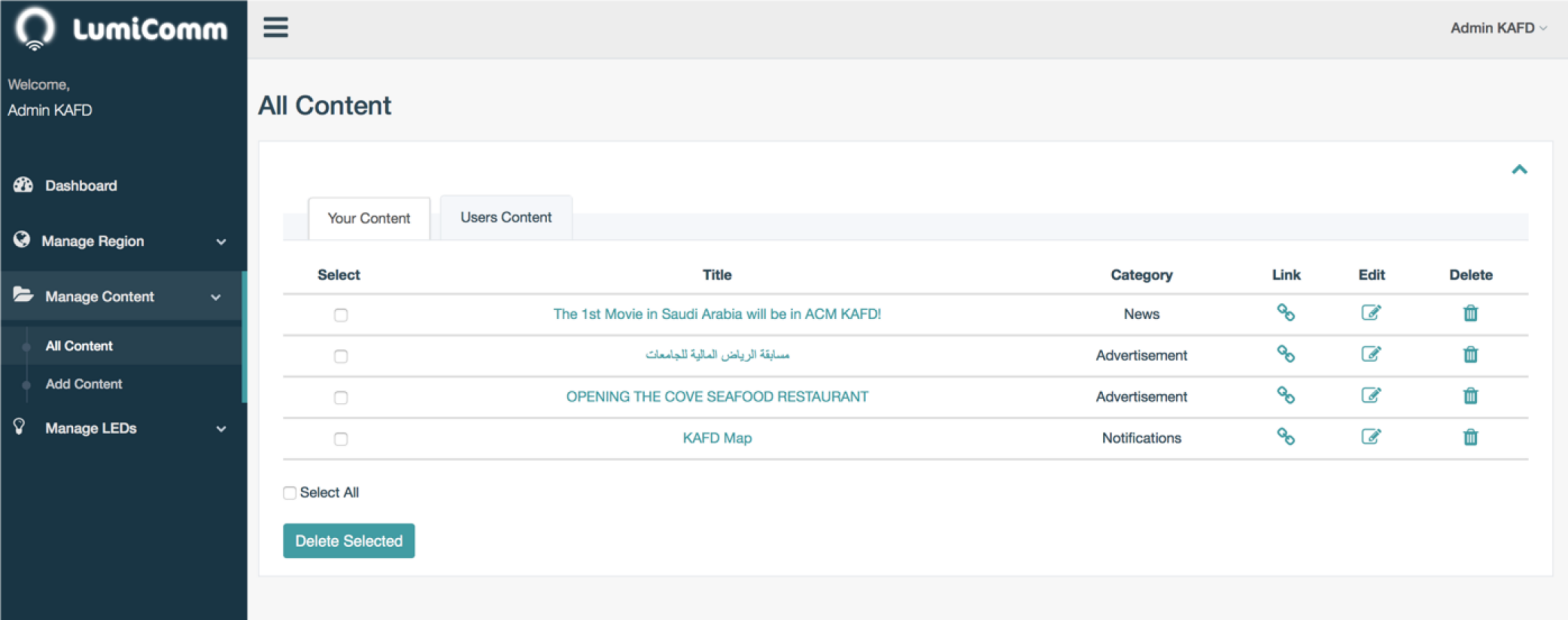
List of content created for KAFD.

**Figure 18:**
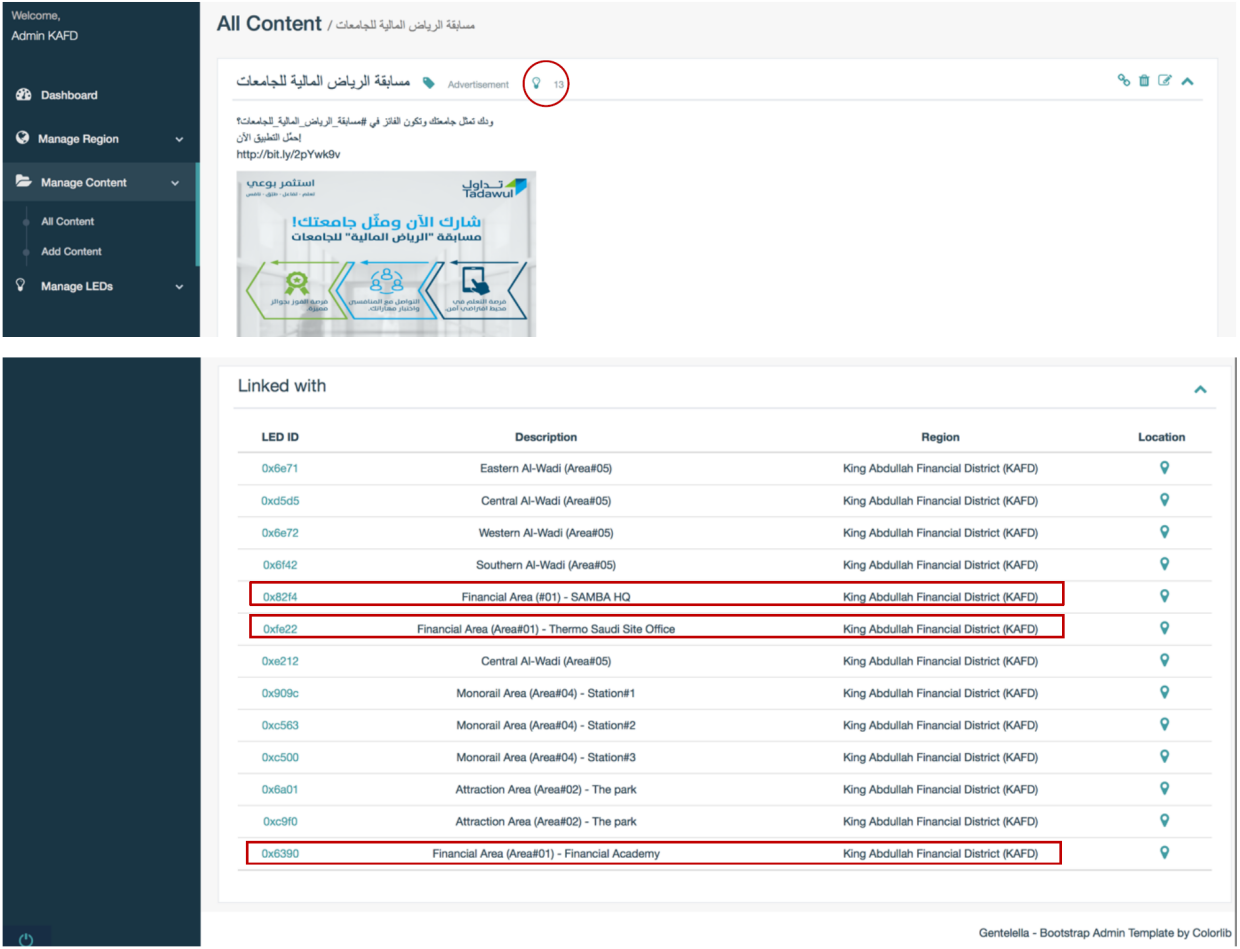
Content view.

In this phase, it can be seen that the CMS of the proposed system give the administrator the ability to manage regions, manage contents and also manage LEDs as shown in Fig. 15. He can map LEDs actual locations to real map that presented in the system. To accomplish this task, the admin has to do the following:

• 1. Register and login to Lumicomm Admin web-panel.
• 2. Create KAFD region, with all the required details as shown in Fig. 16.
• 3. Insert LEDs and their information in KAFD region and map actual location in the real map as shown in Figs. 16 and 17.
• 4. Create the list of content and associate them with specific LEDs or spread it in KAFD region as whole, as shown in Fig. 18.

After the region have been created as it appears in Fig. 16, the final distribution of LEDs’ locations in KAFD can be seen in the map below captured from the admin web-panel of Lumicomm (CMS of the proposed system).

**Figure 19:**
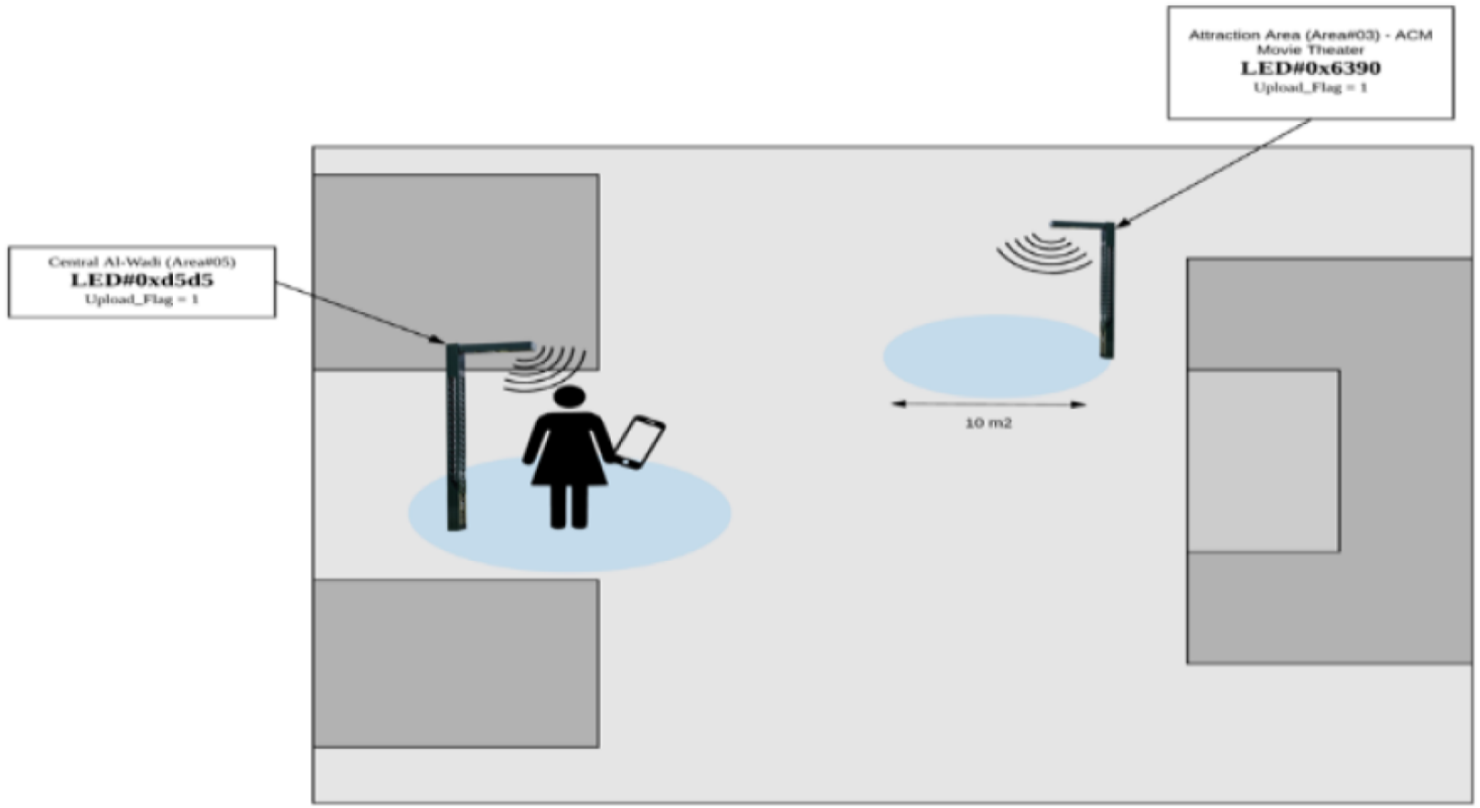
User inside the range of Al-Wadi LED.

The content created by the admin appears in the screenshot below:

As shown in Fig. 18, there is a list of content with their titles, categories and links. The links can show if the content is linked or not with different number of LEDs. This means that the content will be available to be publish by linked LEDs as shown in Fig. 19.

It can be seen that the proposed system give the functions to create the region, add the list of content and link them with LEDs.

4) Testing phase

#### Testing system functionality

In the testing phase, users are required to download Lumicomm Mobile Application to receive or upload the content from/to the LEDs. Assumed that the LEDs, that already running in the testbed, are placed in KAFD and need to be tested according to the following scenarios:

1) LED#0xd5d5 is installed in the Central Al-Wadi

**Figure 20:**
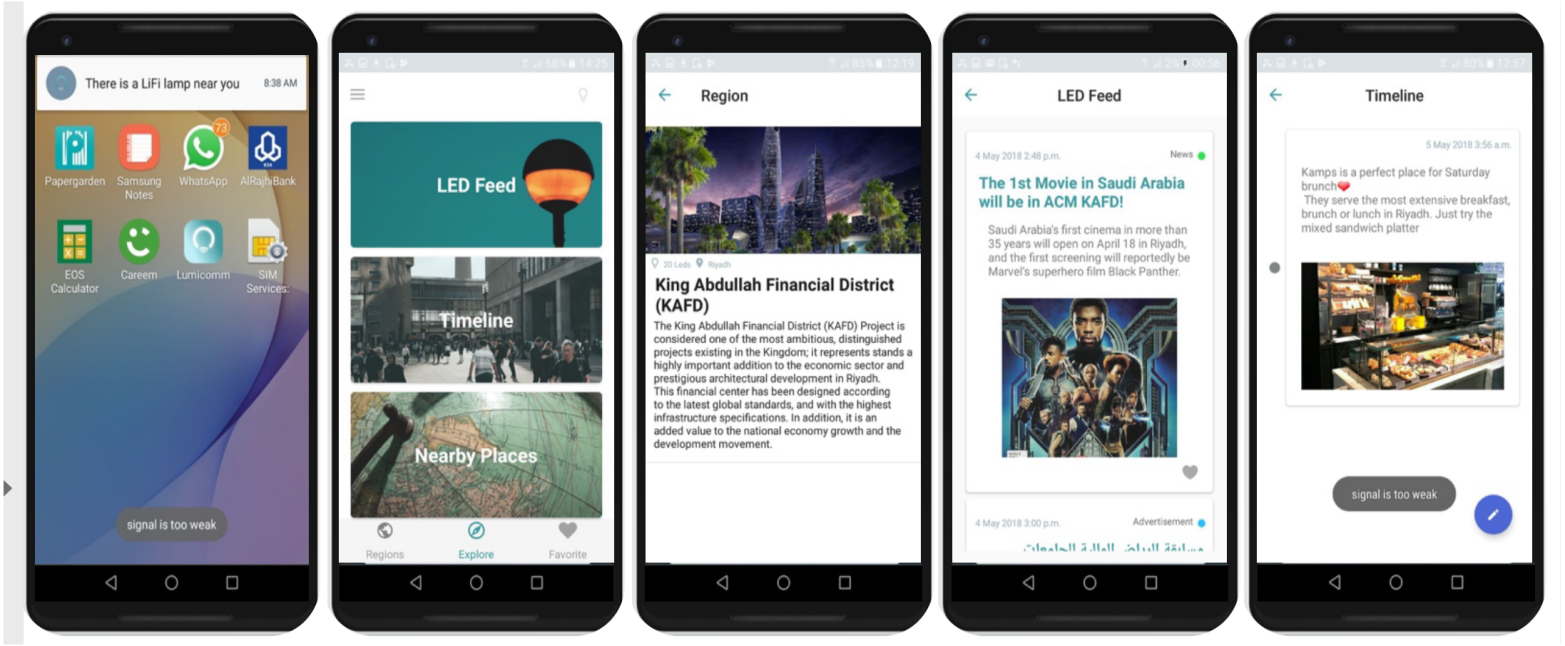
Interfaces of the mobile application of the proposed system inside the Li-Fi region.

When the user is in the coverage range of the Led lamp as shown in Fig. 19, the mobile phone will sense the Li-Fi signal and push notifications accordingly, as it appears in Fig. 20A. When the user opens the application, they can view the current region’s information as appears in part (c). Part (b) shows the options available when the user visits the explore page, which are: LED feed, Timeline and Explore. It can be seen that the content linked with this LED is the movie news and Riyadh competition for universities “ 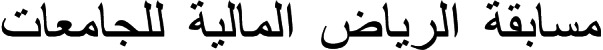
” both created and linked by the admin in the previous step. As for the user’s uploaded content, it appears in Fig. 20E. This functions give the user to upload geo-contextualized that can be collected by the system for further analysis and processing.

**Figure 21:**
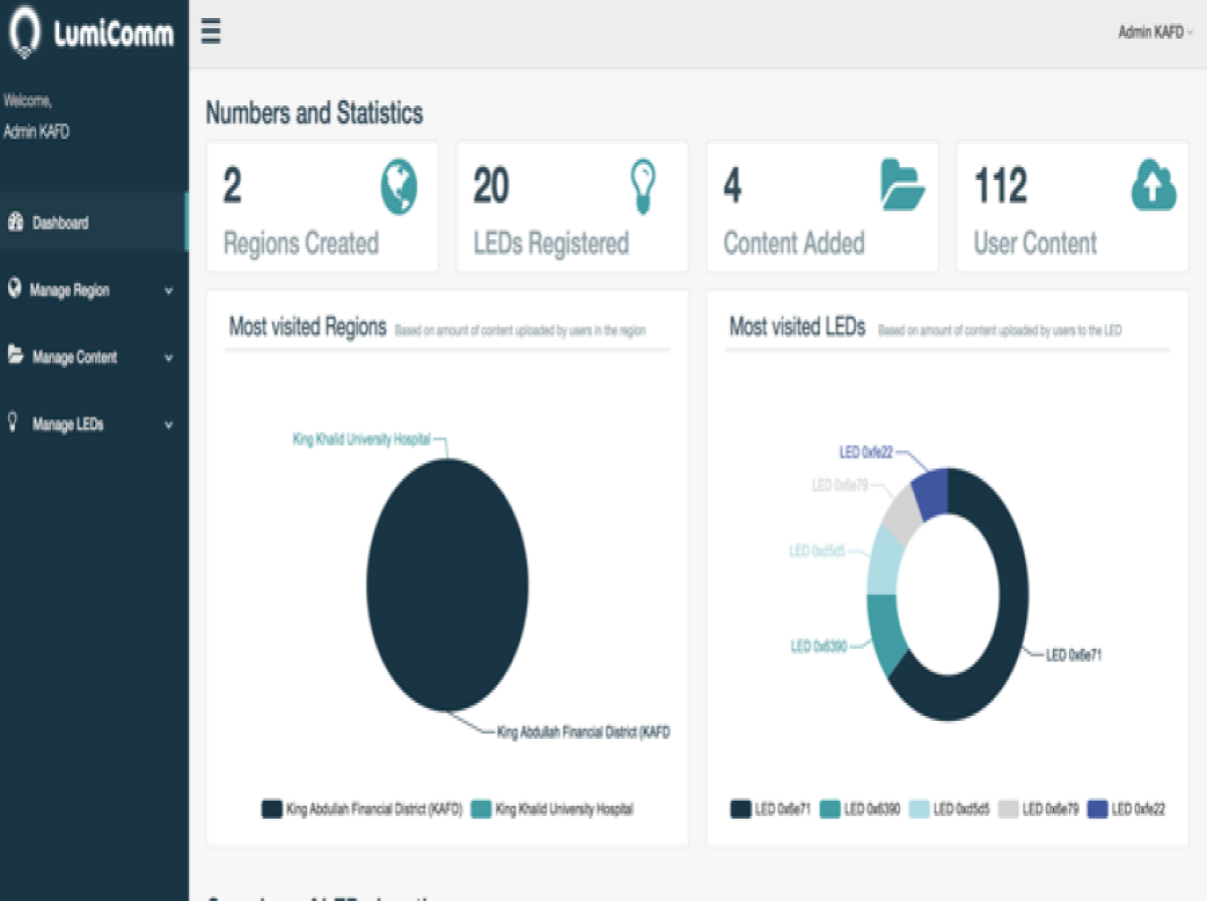
Insights of user interaction with system’s LEDs.

The screenshot in Fig. 21 provides insights of user interactions with the LEDs captured from the admin dashboard after running the system for a while in KAFD. There is 112 user content uploaded with LED 0x6e71 being the most visited LED in KAFD.

The system that consist of CMS and location aware mobile applications are worked successfully and delivered the expected results. The system give the admin the ability to deliver LBS services in different areas of the city by linking the LBS services with the LEDs that exist in specific place. Also, the Location aware mobile application can collect the contextual data of users according to their locations in the city. The system has huge amount of data that categorized by the area which can be used for further analysis and processing in order to help decision makers in different sectors.

### Usability testing

The usability of the system was measured in three criteria: (1) Effectiveness: by measuring the number of errors detected when the user performs a specific function. (2) Efficiency: by measuring the time that the user takes to perform a specific function. (3) Satisfaction: by using a survey to discover the users’ feedback about the system. To test the efficiency and effectiveness of our system, the average time the users need to complete a specific task was computed and the average numbers of errors they have made was registered.

**Figure 22:**
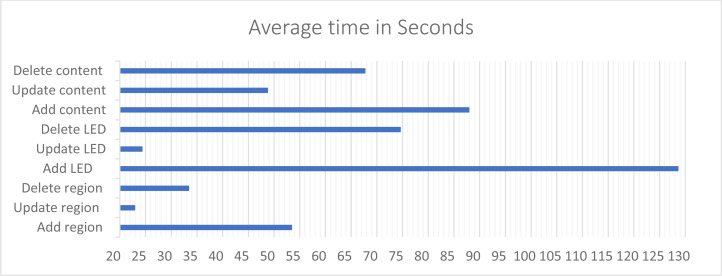
The time that the user takes to perform a specific function in CMS.

The average number of error occurred in add LED functions is 0.2, while it is 0 in the other functions. This indicates the level of ease and learnability of the system. For the response time as shown in Fig. 22, the add LED function took 128.68 s on average, and the related functions such as: update LED details and delete LED took only between 24.4 to 74.64 s to be completed. Notice that adding LEDs to the system took the highest amount of time since it requires more steps to accomplish. Managing LEDs time taken dropped from approximately 128 s in “Add” to 24.4 s in “Update”. Which means once the user got familiar with the interfaces the easiness of use increase.

**Figure 23:**
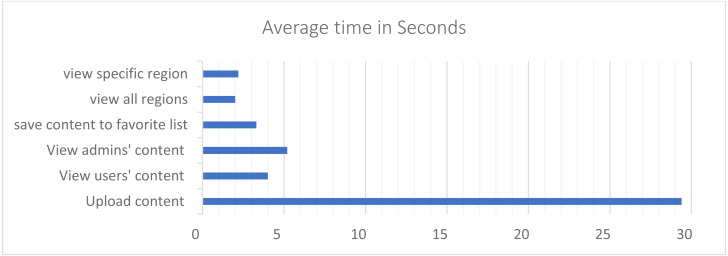
The time that the user takes to perform a specific function in the application.

The average time that users need to complete each function in the application was computed and the average numbers of errors they have made in each function was registered. The average number of error occurred in upload content is 0.2, while it is 0 in all the other functions. That could refer to the fact that uploading content requires more steps than other tasks. The upload content function took 29.4 s on average. In addition, as shown in Fig. 23, view users and admin content took between 4 to 5.2 s on average, and the related functions such as: save content to favorite list and view favorite list took between 3.3 to 3.31 s. Finally, view all regions and view specific region took between 2 to 2.2 s on average. Registering in the system required the highest number of seconds among the functions. When the user first downloads the application, the first function to interact with is registration. According to that, it requires some time until the user gets familiar with how the application works; justifying the gained result. The other functions took noticeable low amount time to accomplish.

To measure the user satisfaction of the system, a survey was made that measures different aspects of the system such as ease of use and learnability, feedback and errors, consistency and screen displays, efficiency and subjective satisfaction. The questions were constructed as seven-point rating scales. Users were asked to rate agreement with the statements, ranging from strongly disagree to strongly agree. The questions were chosen based on several HCI rules measuring usefulness, reliability, usability, consistency, learnability, robustness and satisfaction. The results shows that 80% of users overall satisfied with the system.

## Discussion

### Experiment discussion

After conducting the experiment and testing methods of the proposed system which includes: content management system and location aware mobile application, it can be seen that the system can deliver content utilizing Li-Fi technology. In addition, it provides an important feature in providing the ability to collect geo-contextualized data. Therefore, there is a huge amount of collected data in the system from different area of the city, that can represent as an example: users’ opinions of public services, news, emergency cases, etc. Accordingly, there is a need of further Big data analysis, processing and visualizing in order to improve decision making process in public or private sectors. This can be considered as future work in order in to provide a complete Li-Fi-based IoT architecture.

**Figure 24:**
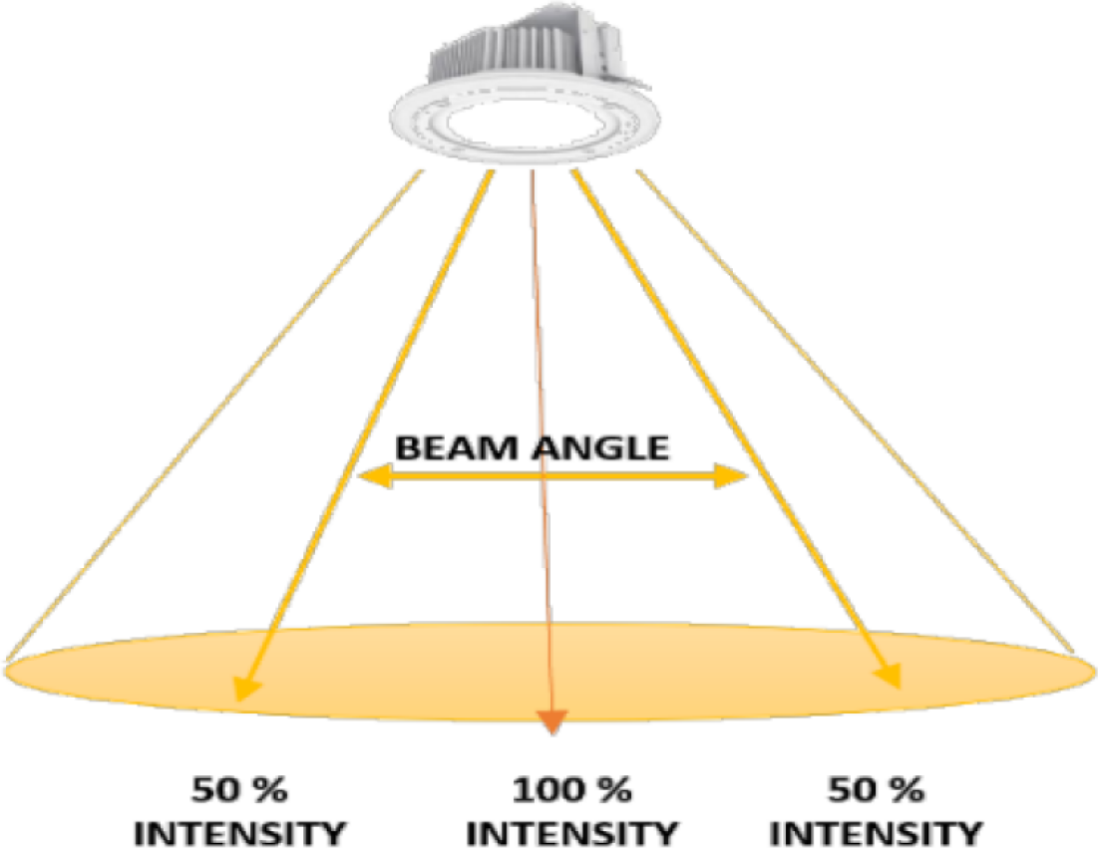
Beam angle and intensity.

Regarding the technical details that related to the testing of Li-Fi technology, and based on the hardware that used in the testbed, there are some observations that can be discussed. The system was tested in indoor and outdoor environment, and it can be seen that the sunlight does not affect the ability of LEDs to deliver the content during the daytime. Regarding the transmission range of the LED lamp, it can be seen that it is affected by different factors. It depends mainly on the power of lamp, so it will be increased significantly when the power of LED is increased. Accordingly, the LEDs that compatiable with camera modulations and having 14.8 W power achieved higher range that the LEDs that less power with 3W. Furthermore, the range also is affected by the beam angle of a LED lamp, which defined as the angle at which the light is emitted. The increasing of beam angle will be resulted in wider coverage area, however, the coverage range will be affected by the intensity as shown in Fig. 24. In fact, the focusing in this paper was in the developing and the implementation of the system utilizing the technology. However, there is a need to conduct a study that taking into consideration the testing of the Li-Fi technology itself and its ability to deliver the information according to different criteria such as: day or night time, coverage range, data rate, *etc.*

It is important to discuss different environmental areas of sustainability for outdoor lighting which are: energy efficiency, and ecological effects of light pollution. The replacing of traditional lighting systems with LED lighting that used by Li-Fi technology generally participates in saving energy due to lower power consumption and longer lifespan of LEDs. It should be mentioned also that there is a need to conduct more reserach on fields include the ecological effects and health effects of light pollution that related to LED lighting. This is important to give better recommendations for sustainable development (Jägerbrand, 2015).

**Figure 25:**
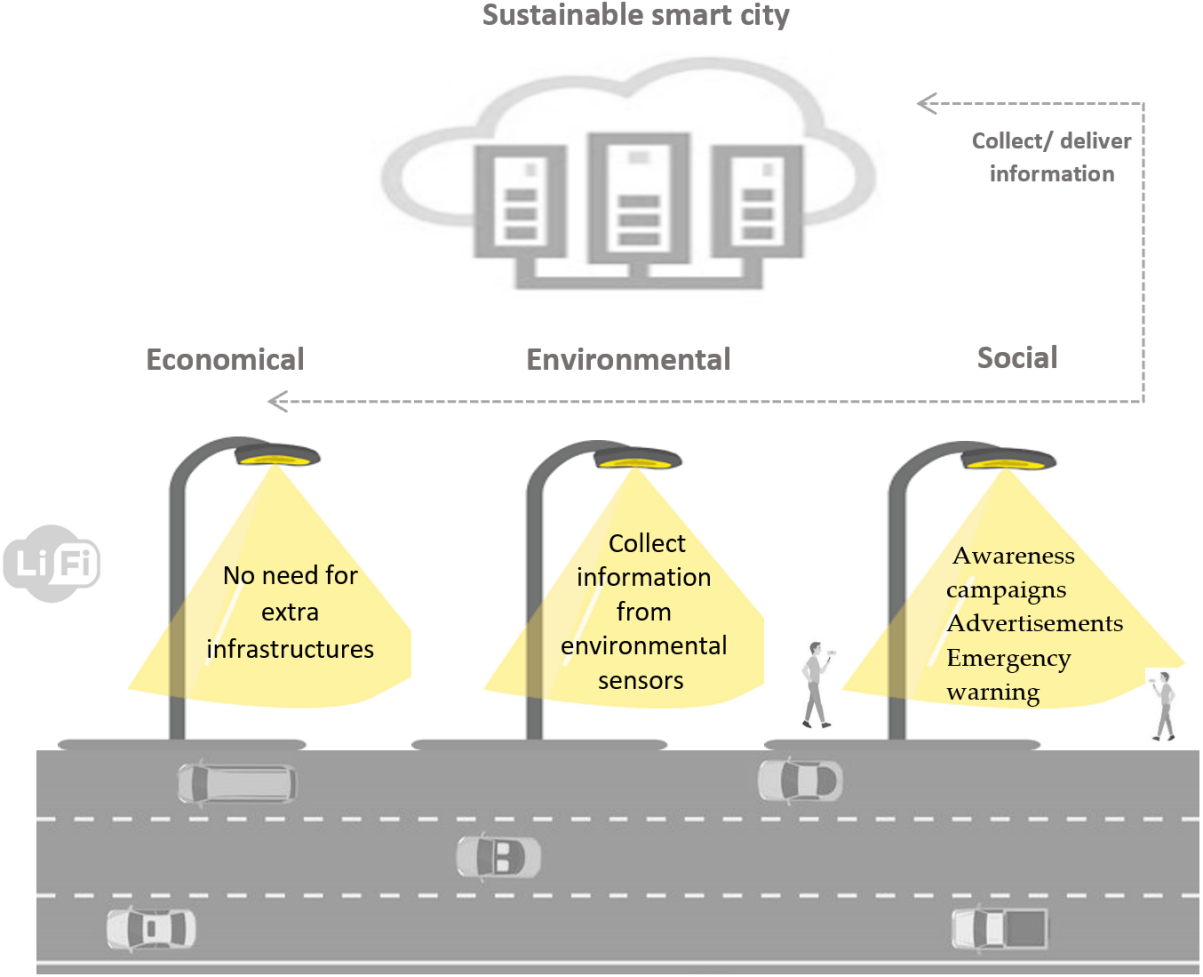
How the proposed system develop a sustainable smart city.

According to the discussion presented in Section 2, and as shown in Table 2 and Fig. 25, it is obvious how the proposed system that designed for smart cities can provide economical solution in the type of technology that used and also variety of smart use cases that can be provided using the same system. In addition, it is shown how the solution is environmental friendly due to its features in saving the energy and also to the ability to monitor different environmental issues. In addition, the system can provide different public services that can improve the quality of life.

**Table 2:** How the proposed system can develop a sustainable smart city.

Economical	Environmental	Social
• Utilize the existing LED bulbs in the smart city for both as light source and data transmission. • Larger spectrum to provide scalable solution that support the increasing number of wireless devices. • Developing the system using Li-Fi can reduce cost of infrastructure than using the Wi-Fi for the same purposes. • LEDs have long lifespan which reduce cost of purchasing lights.	• Used in sensitive environment. • Reduce energy consumption, so reduce Co2 emission. • The lamps also can be attached with different sensors to collect and deliver data using Li-Fi technology. These sensors can be used to monitor different environmental issues in the smart cities. • Participate in waste reduction and saving the environment.	• Enhance the provided services to citizens. • The proposed systems can be used in different use cases in the city. For example Awareness campaigns, advertisements , emergency warning • All of these benefits of the system can improve the public services and increase citizen stratification.

### Conclusion

Li-Fi is a trending technology that is still being under development and improvement. Working on it required considerably great efforts and extensive research, not only in Li- Fi, but also in all the areas related to this technology. Understanding the concept of VLC technologies, how basic LED bulbs are used for more than illumination, how information is encoded into light using different modulation techniques, to how it is decoded and delivered to end users was all necessary to be able to work on the technology. Consequently, the developing of a scalable and generic Li-Fi system will add a great value to the growth of the Li-Fi technology. Accordingly, this article describes the architecture of the GeoLi-Fi System which includes system design, its components and operations. In addition, the detailed implementation and overall setup for the testbed are proposed. The paper also presents a real-world scenario using the proposed system and reports results of the experiment. Moreover, the paper discuss how the proposed system can participate in developing a smart sustainable city. The discussion consider the three pitfalls of sustainability which are economic, environmental and social aspects. The proposed system also can be a foundation for different future smart city applications.

For the future work we aim to do more research on the technology and support its research field. We plan to provide a comprehensive analysis of the technology under various conditions and different environments by measuring data rates, and LED coverage range. In addition, there are different potential research directions that can be considered. The security and privacy of Li-Fi technology is still an open issue. Moreover, the attention should be given to the modulation techniques, which have been considered as the key of Li-Fi communication. Moreover, it is suggested to conduct more reserach to study the sustainability dimensions of Li-Fi technology that related to the ecological effects. Also, there is a need to compare retrofit solutions for street lighting with regards to cost, energy consumption and the provided services.

## Supplemental Information

10.7717/peerj-cs.1009/supp-1 Supplemental Information 1 Code

10.7717/peerj-cs.1009/supp-2 Supplemental Information 2 Raw data

10.7717/peerj-cs.1009/supp-3 Supplemental Information 3 System testing process

10.7717/peerj-cs.1009/supp-4 Supplemental Information 4 Experiment

10.7717/peerj-cs.1009/supp-5 Supplemental Information 5 Questionnaire

10.7717/peerj-cs.1009/supp-6 Supplemental Information 6 Arabic Questionnaire

## Additional Information and Declarations

### Data Availability

The following information was supplied regarding data availability: The code and the raw data are in the Supplemental Files.
