# Supplementary material for: Towards smart sustainable cities using Li-Fi technology: geo-location infrastructure utilizing LED street lights
Source: PeerJ Comput Sci. 2022 Jul 21;8:e1009. doi: 10.7717/peerj-cs.1009 (PMC9454865; doi:10.7717/peerj-cs.1009)
Supplement: Supplemental Information 3 [file peerj-cs-08-1009-s003.pdf]

CHAPTER 1

SYSTEM TESTING

This chapter describes the testing stage of the system. Testing ensures that the system is free of errors and bugs and delivers expected results. Different types of testing methods have been applied on the system which are: unit testing, integration and regression testing, performance and stress testing, test cases and user acceptance testing.

7.1. UNIT TESTING

7.1.1. WEBSITE

Function Class	Component to be tested	Component description	Result
**General Functions**	C1	Register in the system	**Pass**
	C2	Log in the system	**Pass**
	C3	Update profile info	**Pass**
	C4	Display LED(s) on map	**Pass**
	C5	Display LEDs' Statistics	**Pass**
	C6	Log out of the system	**Pass**
**Region Management**	C7	Add region	**Pass**
	C8	Update region details	**Pass**
	C9	Delete region	**Pass**
**LED Management**	C10	Add LED	**Pass**
	C11	Update LED details	**Pass**
	C12	Delete an LED	**Pass**
**Content Management**	C13	Upload content	**Pass**
	C14	Update content details	**Pass**
	C15	Delete content	**Pass**
	C16	Link content with LED	**Pass**

Table 1: Website Unit testing

7.1.2. APPLICATION

Function Class	Component to be tested	Component description	Result

**General Functions**	C1	Register in the system	**Pass**
	C2	Log in the system	**Pass**
	C3	Log out of the system	**Pass**
	C4	Edit profile	**Pass**
	C5	Contact Us	**Pass**
**Users/Admin content Functions**	C6	Upload content	**Pass**
	C7	View users contents	**Pass**
	C8	View admin contents	**Pass**
	C9	Save content to favorite list	**Pass**
	C10	View favorite list	**Pass**
**Region Functions**	C11	View all regions	**Pass**
	C12	View region	**Pass**

Table 2: Mobile Application Unit testing

7.2. INTEGRATION AND REGRESSION TESTING

7.2.1. INTEGRATION TESTING

a. WEBSITE

There are four groups of components that have been tested individually in the previous section (Unit Testing). These groups are: group 1, which contains general functions, group 2 includes the region functions, group 3 contains content functions and group 4 contains LEDs functions. In this section, we integrate the components one by one for each group and test the integration of the components to make sure it works as expected.

Test sequence			
**Test Number**	Test 1	Test 2	Test 3
**Component**	Integrating C1 and C2	Integrating C3 and Test1	Integrating C4, C5 and Test2
**Description**	Test Register and Login.	Test Update profile info with component of Test1	Test Display LED(s) on map and Display LEDs' Statistics with component of Test 2
**Graph**	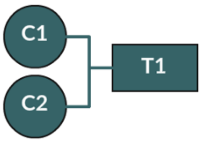	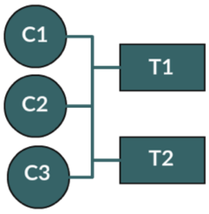	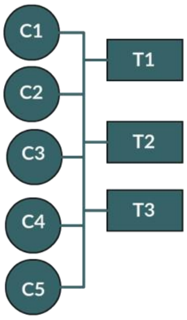
**Status**	Pass	Pass	Pass

Table 3: Website Integration testing 1

Test sequence			
**Test Number**	Test 4	Test 5	Test 6
**Component**	Integrating C6 and Test 3	Integrating C7,C8 and Test4	Integrating C9 and Test 5
**Description**	Test Logout with component of Test 3	Test Add region ,Update region with component of Test 4	Test Delete region with component of Test5

**Graph**	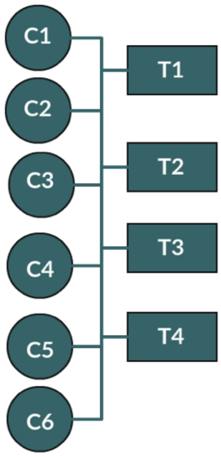	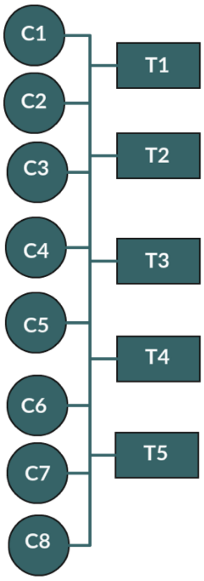	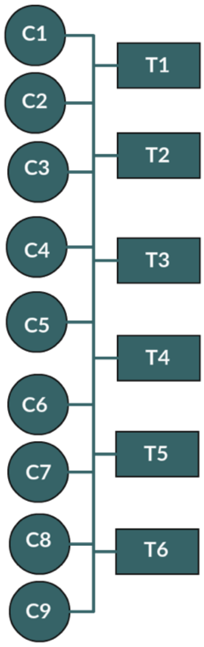
**Status**	Pass	Pass	Pass

Table 4: Website Integration testing 2

**Test sequence**			
**Test Number**	Test 7	Test 8	Test 9
**Component**	Integrating C10,C11 and Test6	Integrating C12 and Test 7	Integrating C13, C14 and Test8
**Description**	Test Add LED and Update LED details with component of Test 6	Test Delete LED with component of Test 7	Test Upload content Update content details, with component of Test 8

**Graph**	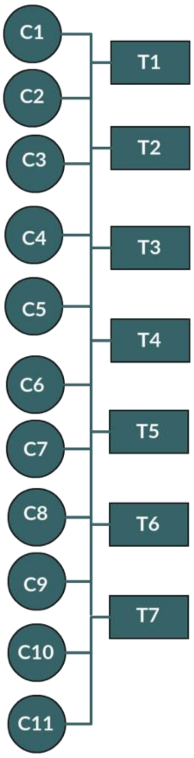	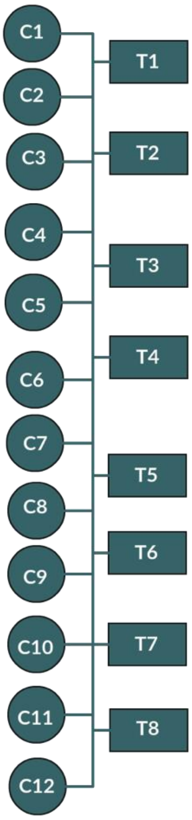	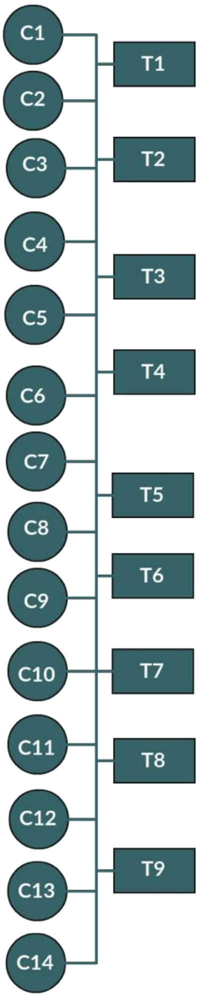
**Status**	Pass	Pass	Pass

Table 5: Website Integration testing 3

**Test sequence**		
Test Number	Test 10	Test 11
Component	Integrating C15 and Test 9	Integrating C16 and Test 10
Description	Test Delete content with component of Test 9	Test Link content with component of Test 10

<table>
<tr><td>Graph</td><td>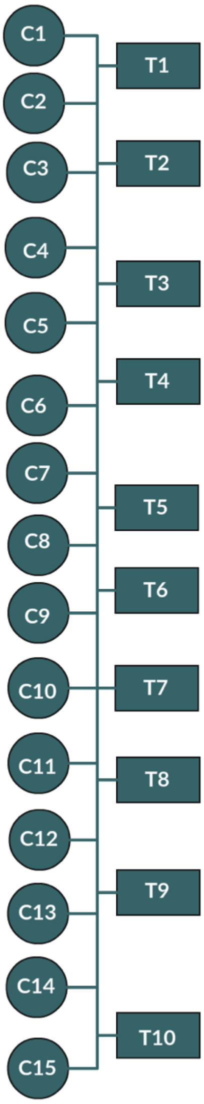
</td><td>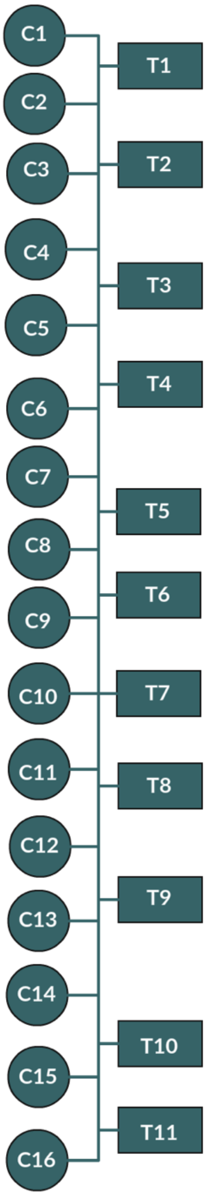
</td></tr>
<tr><td>Status</td><td>Pass</td><td>Pass</td></tr>
</table>

Table 6: Website Integration testing 4

b. APPLICATION

We applied the integration testing in the application by the following steps:

Test sequence			
**Test Number**	Test1	Test2	Test3
**Component**	Integrate C1 and C2	Integrate C3 and Test1	Integrate C4 and Test2
**Description**	Test Register and Log in.	Test Log out and component of Test1	Test Edit profile and component of Test2
**Graph**	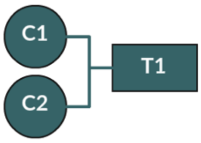	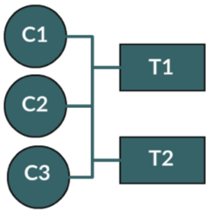	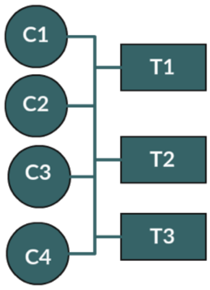
**Status**	Pass	Pass	Pass

Table 7: Application integration testing 1

Test sequence			
**Test Number**	Test4	Test5	Tes6
**Component**	Integrate C5 and Test3	Integrate C6 and Test4	Integrate C7 and Test5
**Description**	Test Contact us and component of Test3	Test Upload content and component of Test4	Test View users content and component of Test5
**Graph**	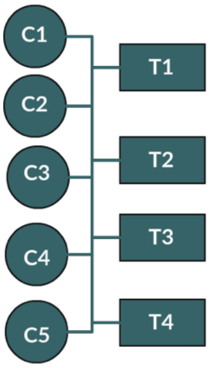	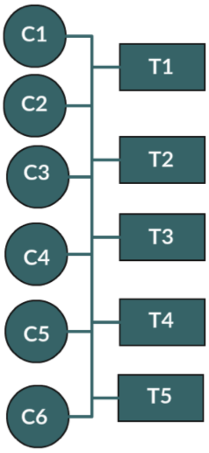	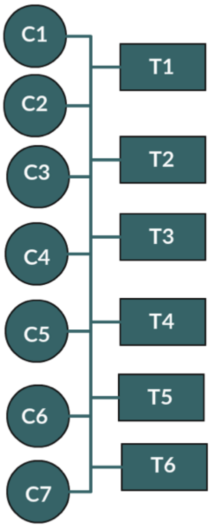

**Status**	Pass	Pass	Pass

Table 8: Application integration testing 2

**Test sequence**			
**Test Number**	Test7	Test8	Tes9
**Component**	Integrate C8 and Tes6	Integrate C9 and Test7	Integrate C10 and Test8
**Description**	Test View admin content and component of Test6	Test Save content to favorite list and component of Test7	Test View favorite list and component of Test8
**Graph**	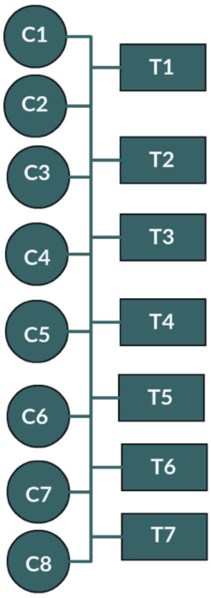	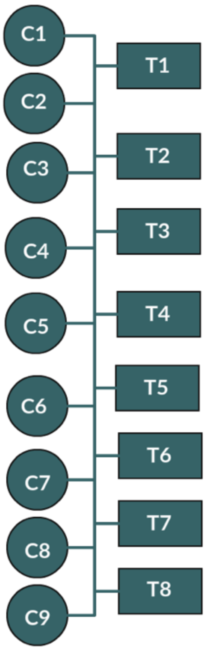	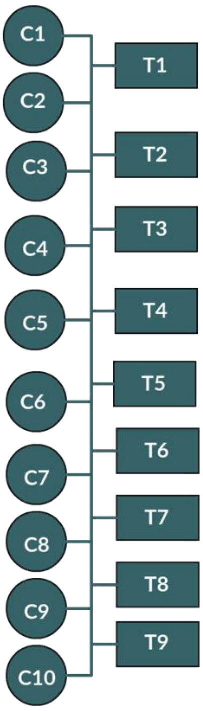
**Status**	Pass	Pass	Pass

Table 9: Application integration testing 3

**Test sequence**		
**Test Number**	Tes10	Tes11
**Component**	Integrate C11 and Test9	Integrate C12 and Test10
**Description**	Test View all regions and component of Test9	Test View region and component of Test10

**Graph**	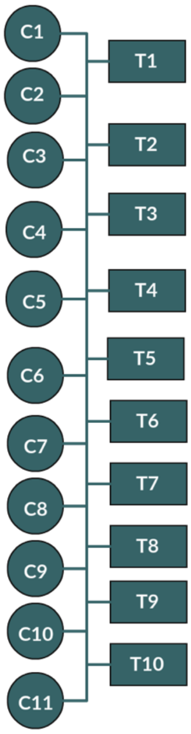	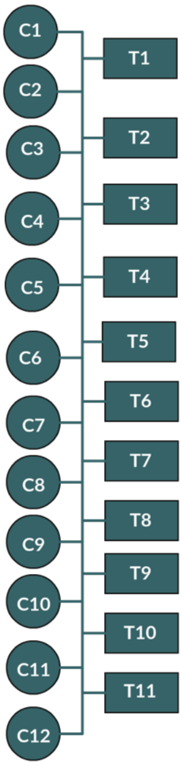
**Status**	Pass	Pass

Table 10: Application integration testing 4

7.2.2. REGRESSION TESTING

Regression testing is a type of software testing carried out to ensures that previously tested code is still performs the way it should be after new changes. Changes may include enhancements, patches, configuration changes.

We applied the regression test by testing the whole functionalities of the system after any change or update, to make sure that the system functionalities are still working as expected.

7.3. TEST CASES

7.3.1. WEBSITE

1. Register Test Cases

Test Case: #1	Description	Action	Expected Result
**Leave empty fields**	The user leaves some or all fields empty	Click on Register button	An error message will be displayed prompting the user to complete all fields
**Actual Result**			
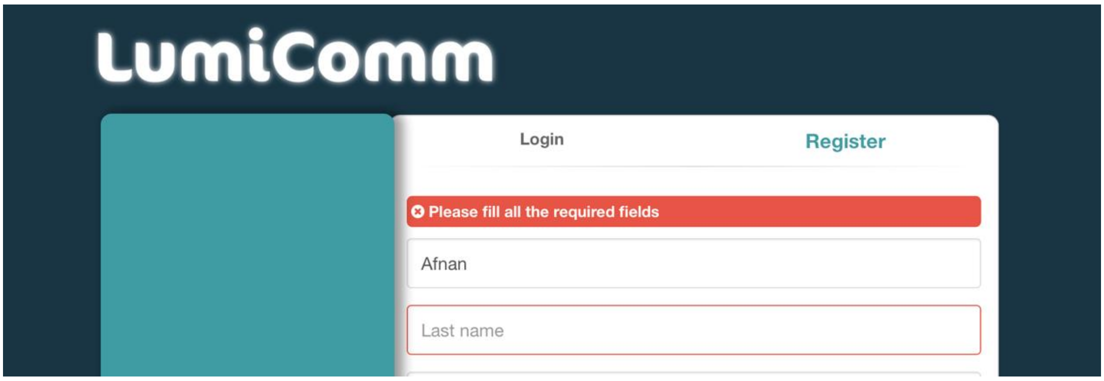			
**Pass?**			
Yes			

Table 11: Register test case with empty field

Test Case: #2	Description	Action	Expected Result
**Enter invalid email**	The user enters an email in an incorrect format.	Click on Register button	An error message will be displayed prompting the user to enter a valid email
**Actual Result**			
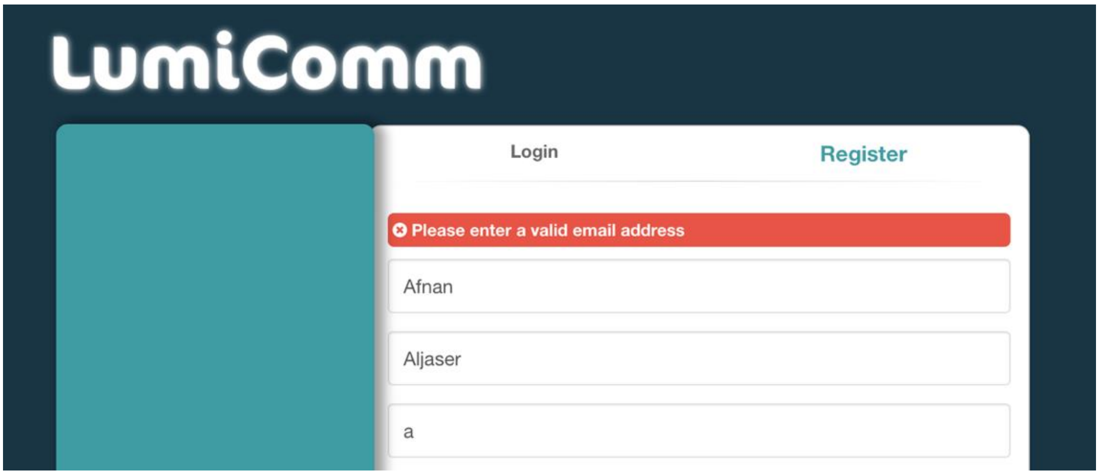			

Table 12: Register test case with empty field

Pass?
Yes

Table 13: Register test case with registered email

Test Case: #3	Description	Action	Expected Result
**Enter email that is already registered**	The user enters a registered email	Click on Register button	An error message will notify the user that the email is already registered
**Actual Result**			
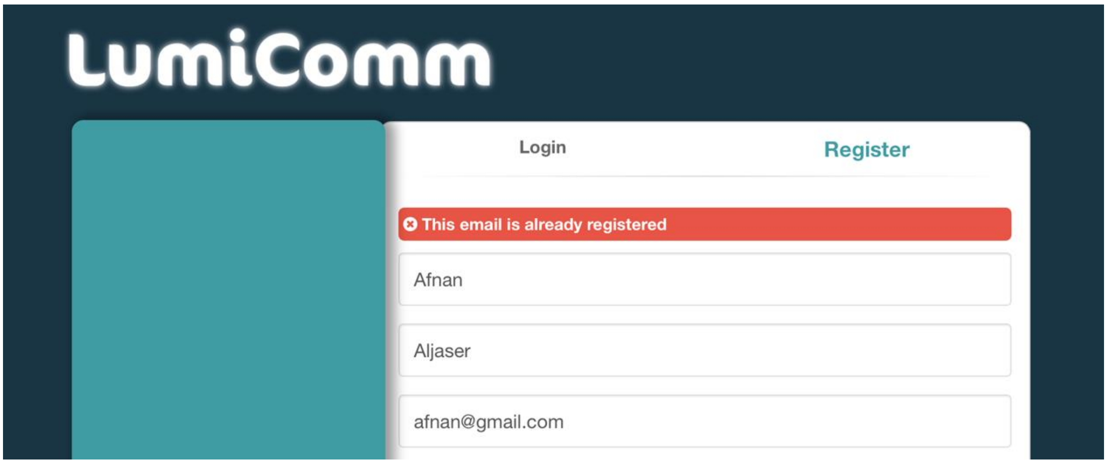			
**Pass?**			
Yes			

2. Login Test Cases

Table 14: Failed log in test case

Log in Test Cases			
**Test Case: #1**	**Description**	**Action**	**Expected Result**
**Enter valid inputs**	The user enters valid inputs	Click on Log in button	The user will be redirected to the dashboard.
**Actual Result**			
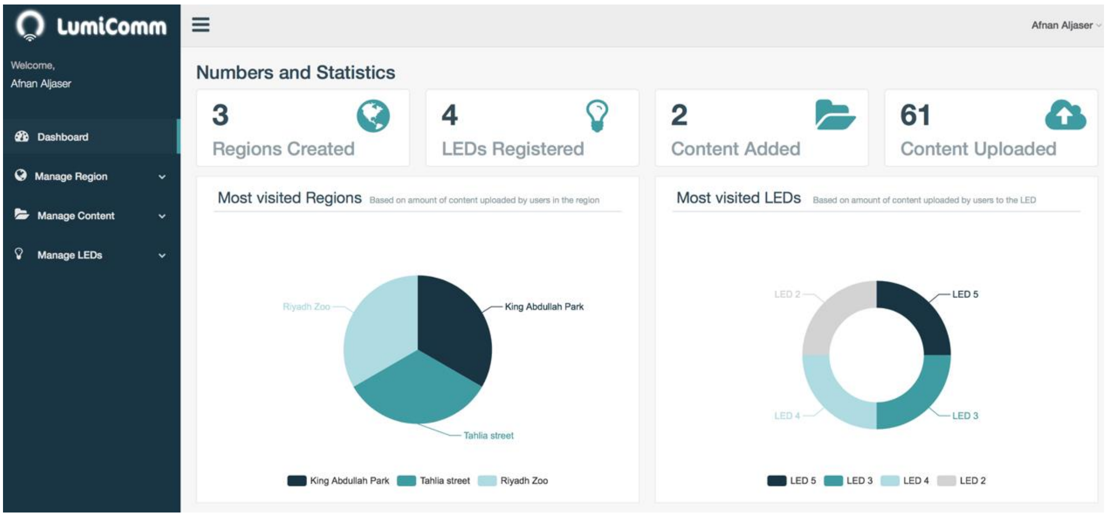			
**Pass?**			
Yes			

**Test Case: #2**	**Description**	**Action**	**Expected Result**
**Leave an empty fields**	The user leaves some or all fields empty	Click on Log in button	An error message will be displayed prompt the user to complete all fields
**Actual Result**			
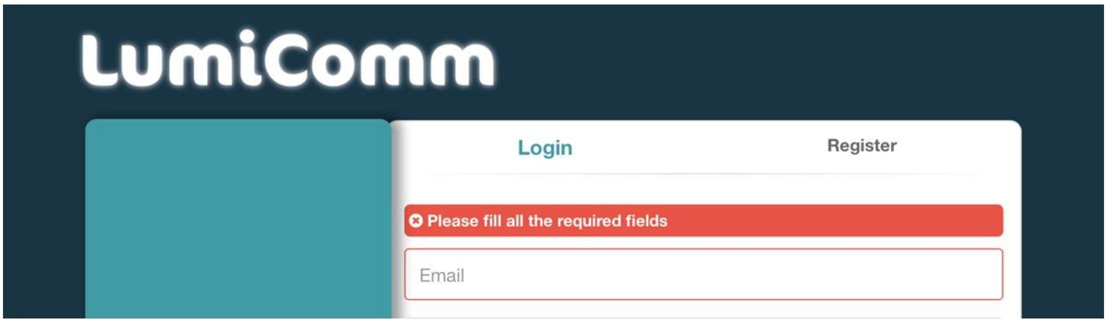			
**Pass?**			
Yes			

Table 15: Log in test case with empty field

**Test Case: #3**	**Description**	**Action**	**Expected Result**

Enter invalid email or password	The user enters invalid inputs	Click on Log in button	An error message will be displayed prompt the user to enter valid inputs
**Actual Result**			
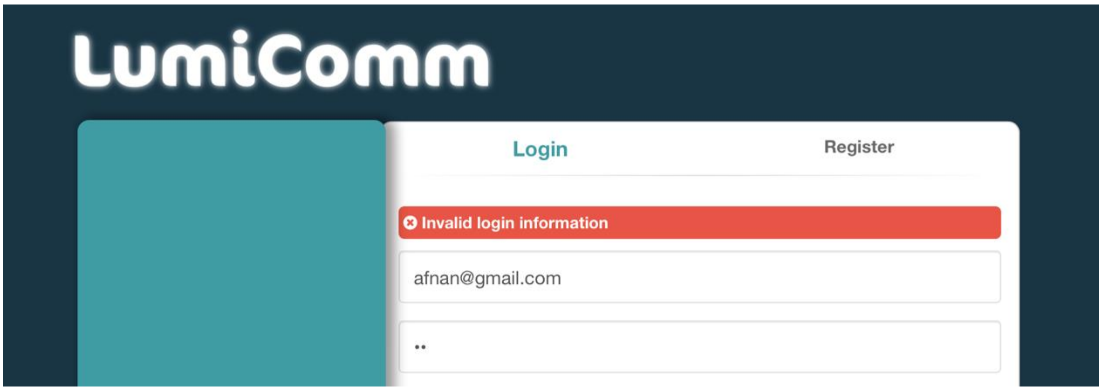			
**Pass?**			
Yes			

Table 16: Log in test case with invalid field

3. Manage Content Test Cases

These test cases show the different scenarios that a user may experience when trying to **add** or **update** a content in the system.

**Manage Content Test Cases**			
**Test Case: #1**	**Description**	**Action**	**Expected Result**
**Enter valid inputs**	The user enters valid information	Click on Add / Update content	The content will be added/updated and a success message will be displayed.<br>If ***add***: the user will be redirected to all content page.<br>If ***update***: The user will be redirected to view content page.
Actual Result (**Add**)			

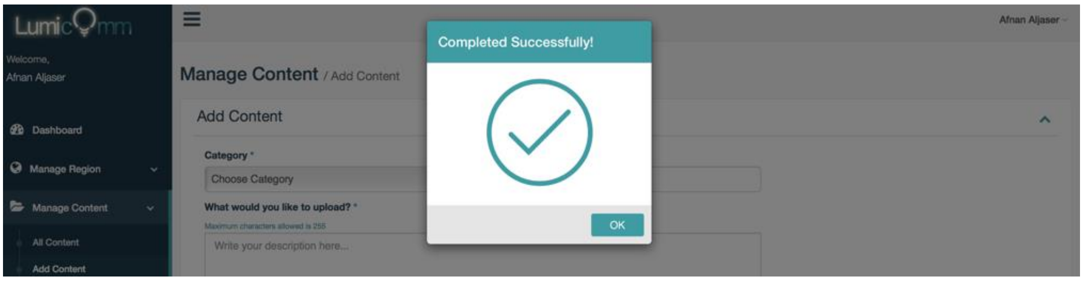

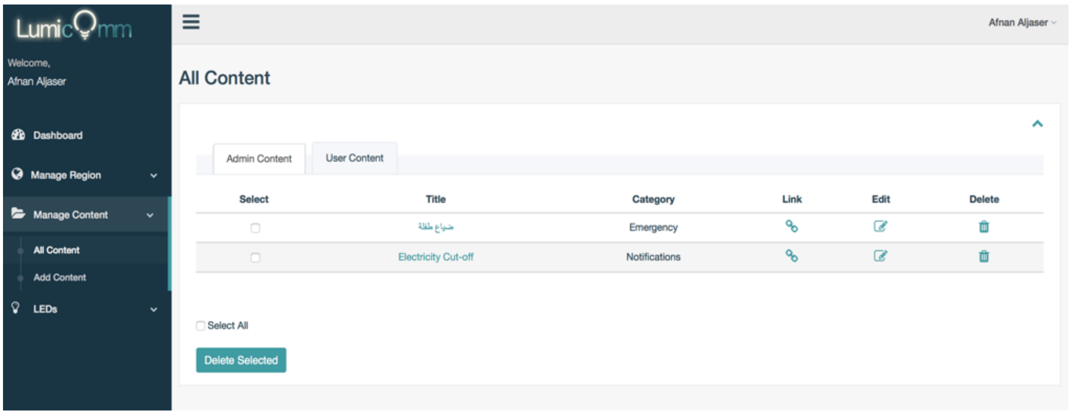

Pass?
Yes

Test Case: #2	Description	Action	Expected Result
**Leave empty fields**	The user leaves some or all fields empty	Click on Add / Update content	Error messages will be displayed prompt the user to complete empty fields.

Actual Result

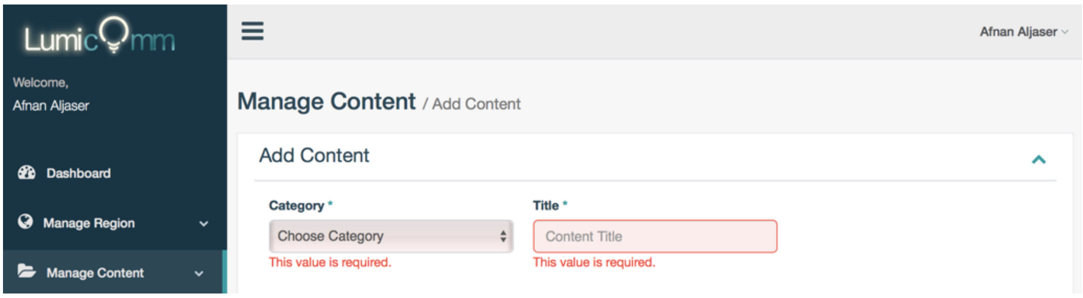

Pass?
Yes

Test Case: #3	Description	Action	Expected Result

**Exceed the accepted number of characters of content description field**	The user enters more than 255 character in content description field	Click on Add / Update content	Content description field will be blocked and its border color will be changed to red.
Actual Result			

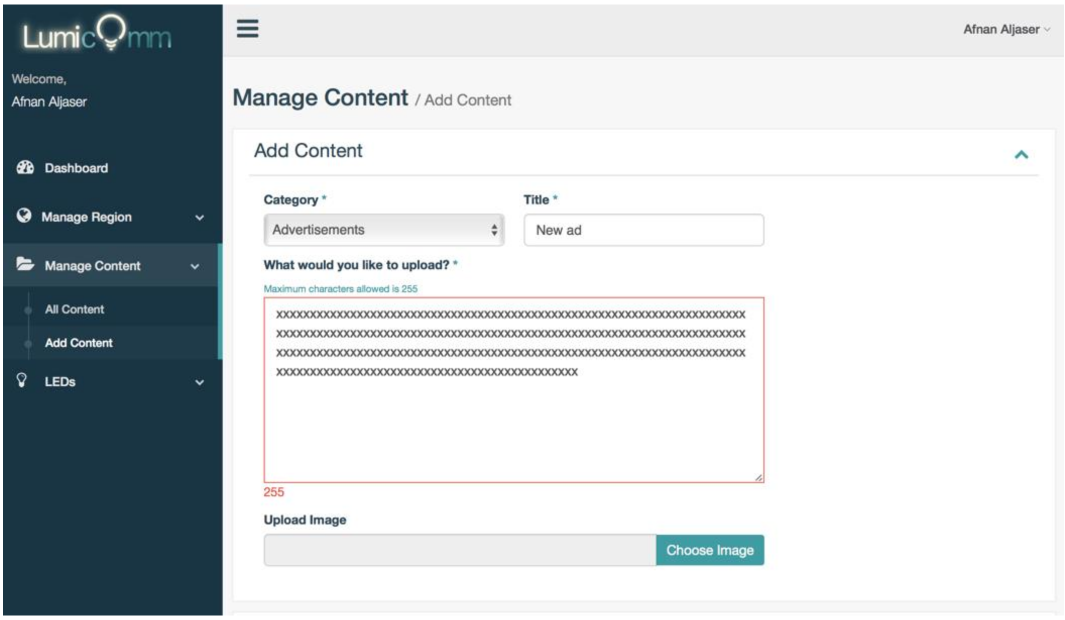

**Pass?**	
Yes	

4. Manage Content Test Cases

These test cases show the different scenarios that a user may experience when trying to **create** or **update** a region in the system.

**Manage Region Test Cases**			
**Test Case: #1**	**Description**	**Action**	**Expected Result**
**Enter valid inputs**	The user enters valid information	Click on Create / Update region	The region will be added/updated and a success message will be displayed.<br>If ***add***: the user will be redirected to all regions page.<br>If ***update***: The user will be redirected to view region page.
Actual Result(**Add**)			

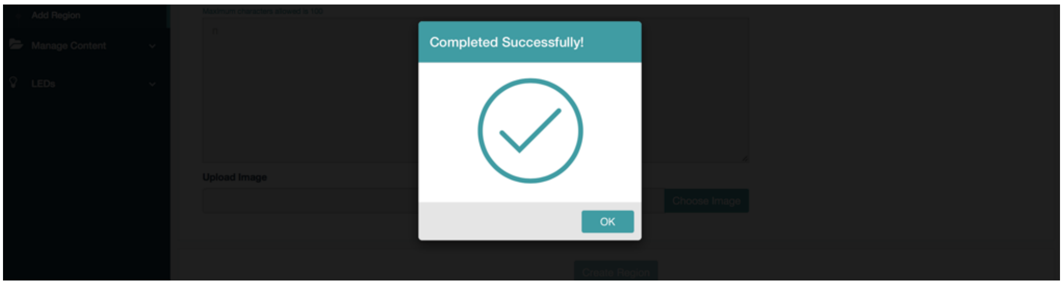 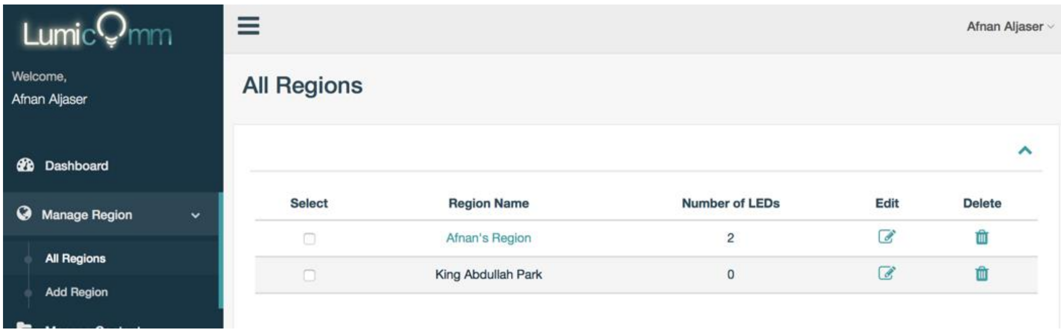
**Pass?**
Yes

Table 17: Manage Content test Cases

**Test Case: #2**	**Description**	**Action**	**Expected Result**
**Leave empty fields**	The user leaves some or all fields empty	Click on Create / Update region	Error messages will be displayed prompt the user to complete empty fields
Actual Result(**Add**)			
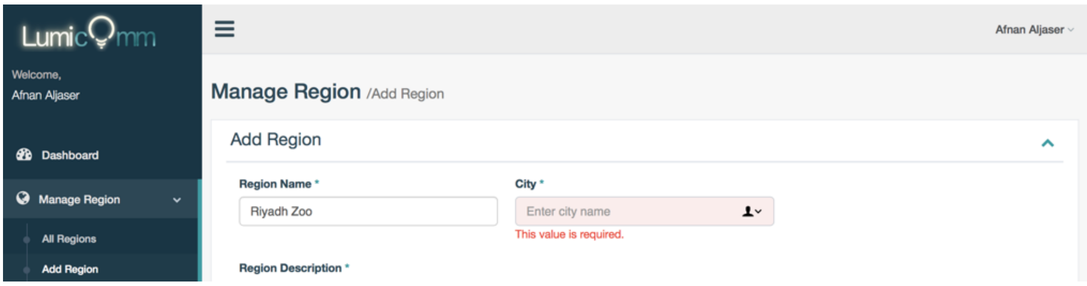			
**Pass?**			
Yes			

**Test Case: #3**	**Description**	**Action**	**Expected Result**
**Exceed the accepted number of characters in region**	The user enters more than 90 character in region description field	Click on Create / Update region	Region description field will be blocked and its border color will be changed to red

**description field**			
Actual Result (**Add**)			
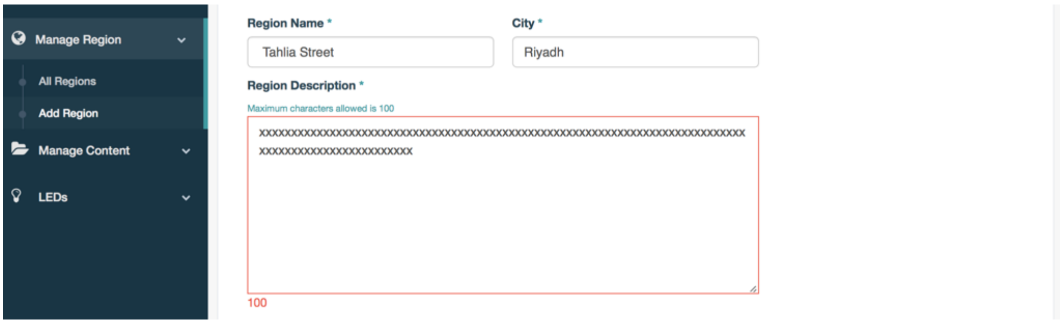			
**Pass?**			
Yes			

Table 18: Manage Content test cases 2

5. Manage LEDs Test Cases

These test cases show the different scenarios that a user may experience when trying to **add** or **update** a LED information.

Test Case: #1	Description	Action	Expected Result
**Leave empty fields**	The user leaves some or all fields empty	Click on Add / Update LED	Error messages will be displayed prompt the user to complete empty fields.
Actual Result(**Add**)			
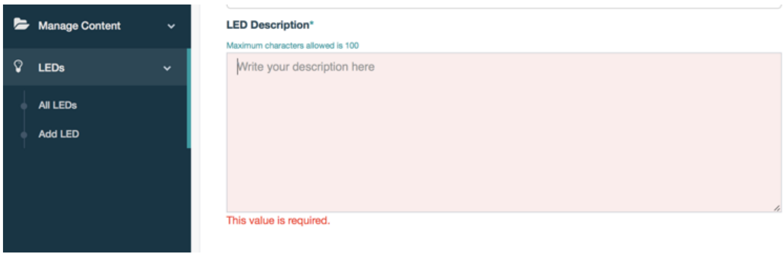			
**Pass?**			
Yes			

Table 19: Manage Led Test Cases 1

Test Case: #2	Description	Action	Expected Result
**Exceed the accepted number of characters in LED**	The user enters more than 100 character in region description field	Click on Add / Update LED	LED description field will be blocked and its border color will be changed to red

**description field**			
Actual Result (**Add**)			
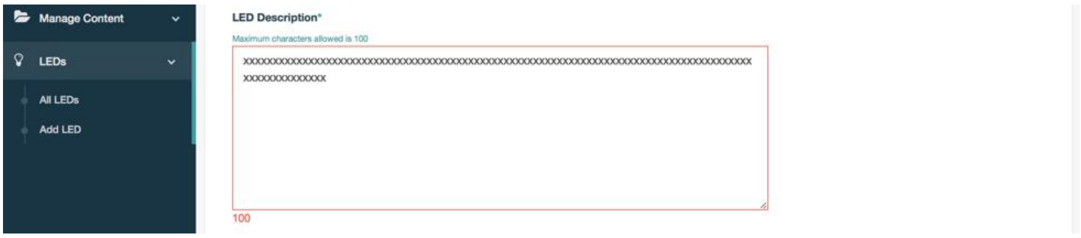			
**Pass?**			
Yes			

Table 20 : Manage Led Test Cases 2

6. View (Region, content and LEDs)

**View Test Cases**		
**Test Case: #1**	**Description**	**Action**
**Delete (Region, Content or LED)**	The user clicks on delete icon to delete a region/content/LED	Click on delete icon
**Expected Result (Delete Region)**	**Expected Result (Delete Content)**	**Expected Result (Delete LED)**
A confirmation message will be displayed, if the user presses Yes, then the region will be deleted, and a success message will be displayed.<br>If the user presses No, then the system will display the region	A confirmation message will be displayed if the user presses Yes, then the content will be deleted, and a success message will be displayed.<br>If the user presses No, then the system will display the content	A confirmation message will be displayed if the user presses Yes, then the LED will be deleted, and a success message will be displayed.<br>If the user presses No, the system will display the LED
Actual Result (Delete a **Region**)		
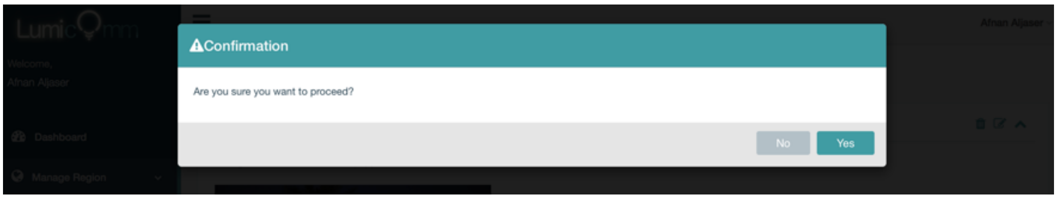		

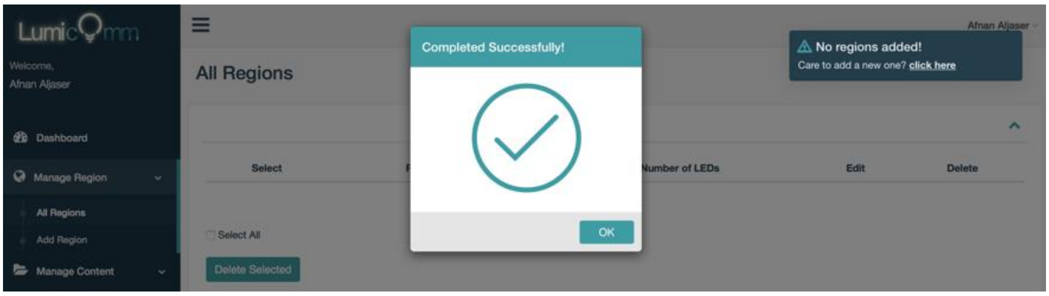		
**Pass?**		
Yes		

Table 21 : View (Region , Content , Led) test cases

**Test Case: #2**	**Description**	**Action**
**Update (Region, Content or LED)**	The user clicks on edit icon of an item	Click on edit icon of an item
**Expected Result (Region)**	**Expected Result (Content)**	**Expected Result (LED)**
The user will be redirected to update region page	The user will be redirected to update content page	The user will be redirected to update LED page
Actual Result (**Region**)		
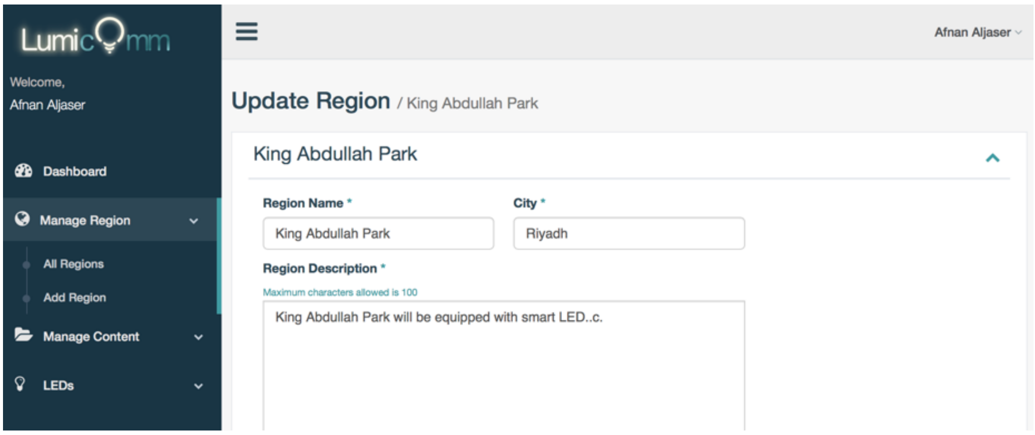		
**Pass?**		
Yes		

Table 22: Update test cases

7. View All (Region, content and LEDs) Test Cases

These test cases show the different scenarios that a user may experience when exploring all region\content\LEDs pages.

**Test Case: #1**	**Description**	**Action**	**Expected Result**

**Select all checkboxes**	The user clicks on select all checkbox	Check on select all checkbox	All checkboxes will be selected
**Actual Result**			
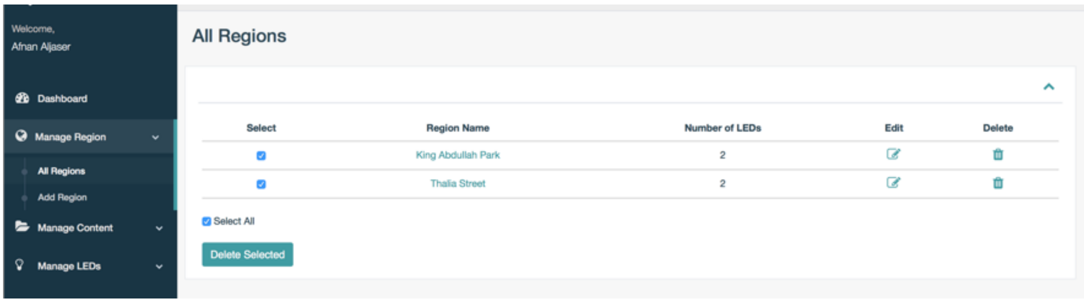			
**Pass?**			
Yes			

Table 23: Select all checkboxes test case

Table 24:

Test Case: #2	Description	Action	Expected Result
**Delete multiple regions**	The user clicks on delete selected button	Click delete selected button	A confirmation message will be displayed if the user presses Yes then the selected regions will be deleted, and a success message will be displayed. If the user presses No, then the system will display All-regions page.
**Actual Result**			
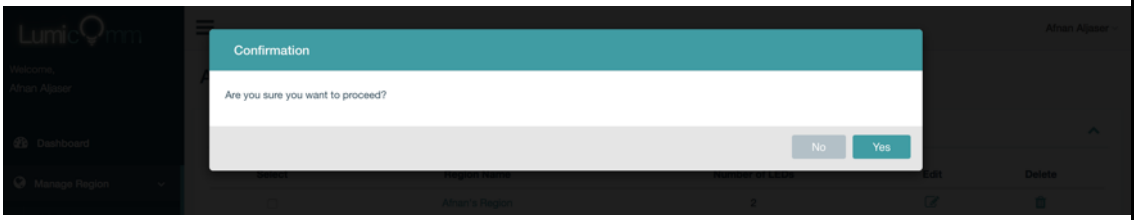 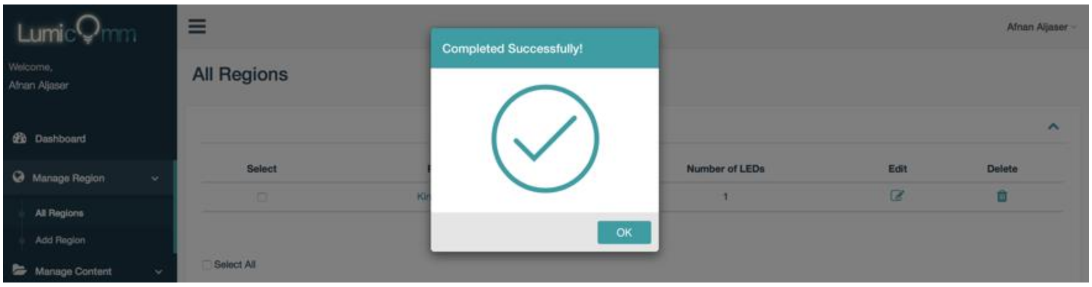			
**Pass?**			
Yes			

Delete multiple regions test case

Test Case: #3	Description	Action	Expected Result
**Delete selected Without selecting any region**	The user clicks on delete selected button without selecting any region	Click on delete selected button	An error message will be displayed prompt the user to select at least one region

Actual Result

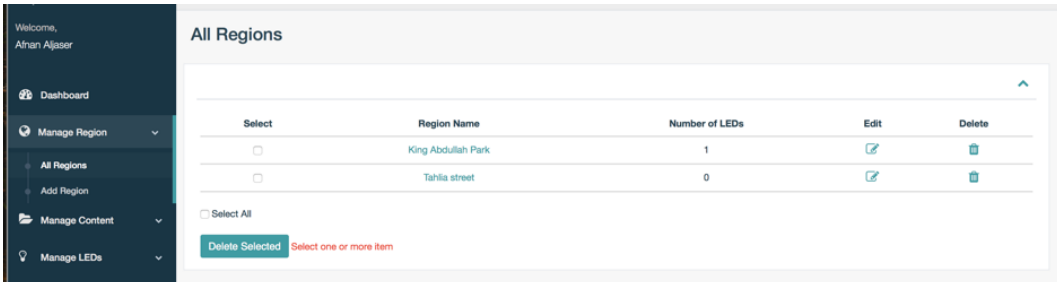

Pass?

Yes

Table 25: Delete selected without selecting any region test case

8. Link\uUnlink Content Test Cases

Link Content Test Cases

Test Case: #1	Description	Action	Expected Result
**Click on link button**	The user clicks on a link button of a content	Click on a link button	The content will be linked with the selected LED and the link button will be changed to Unlink

Actual Result

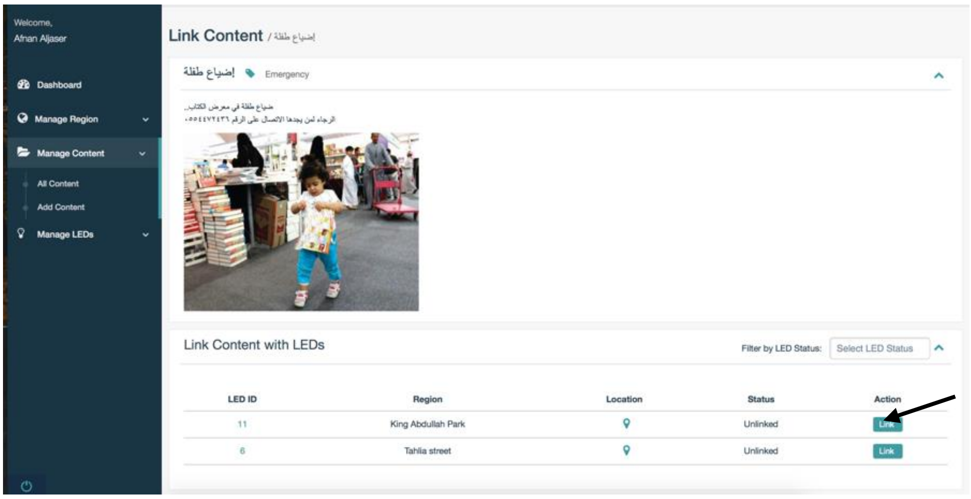

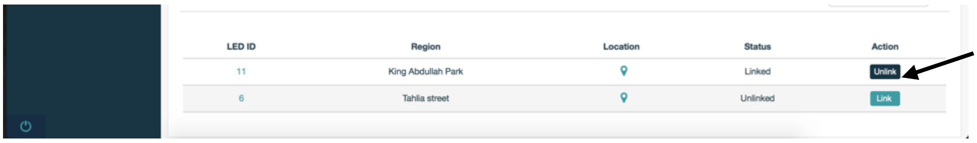
**Pass?**
Yes

Table 26: Link content test case

**Test Case: #2**	**Description**	**Action**	**Expected Result**
**Click on Unlink button**	The user clicks Unlink button of a LED	Click on Unlink button	A confirmation message will be displayed, if the user clicks on Yes, then the content will be unlinked from the selected LED and the unlink button will be changed to link.<br>If the user clicks on No, then the system will display link content page.
**Actual Result**			
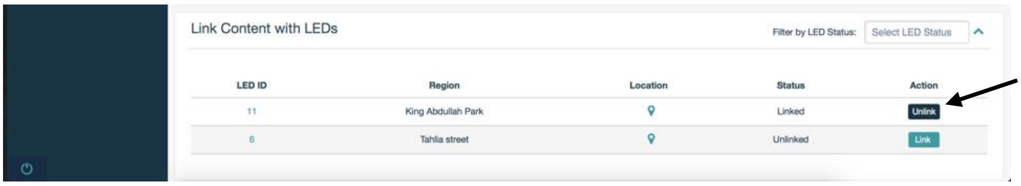 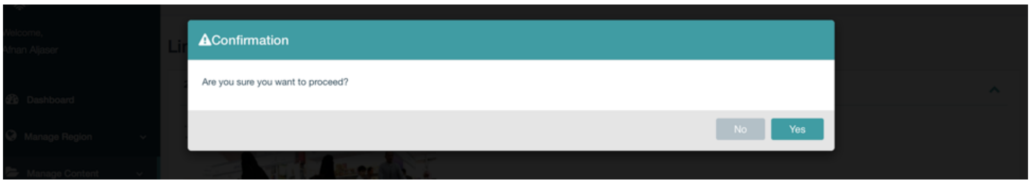 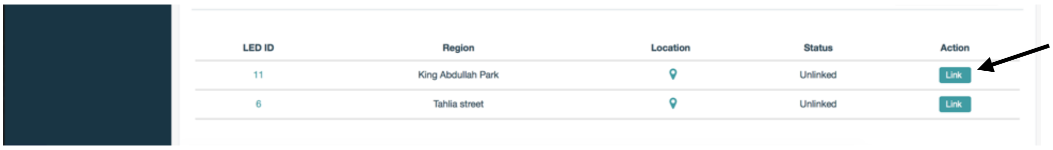			
**Pass?**			
Yes			

Table 27: Click on Unlink button test case

9. Manage Profile Test Cases

Test Case: #1	Description	Action	Expected Result
**Leave an empty field**	The user leaves empty fields	Click on Update Profile button	An error message will be displayed prompt the user to complete all fields
**Actual Result**			
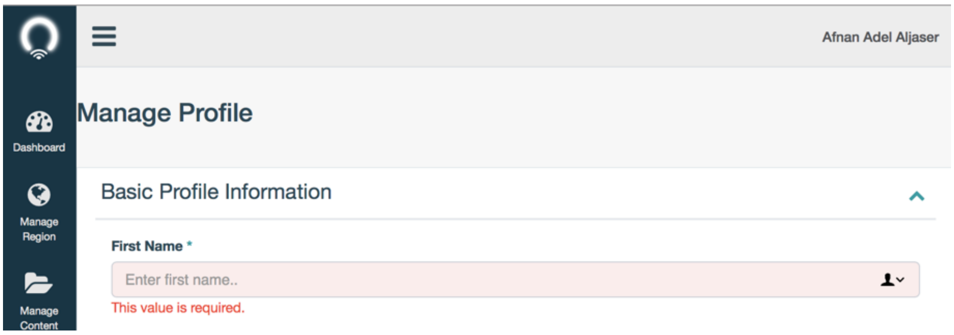			
**Pass?**			
Yes			

Table 28: Manage profile test case 2

Test Case: #2	Description	Action	Expected Result
**Enter invalid email**	The user enters invalid email	Click on Update Profile	An error message will be displayed prompt the user to enter a valid email
**Actual Result**			
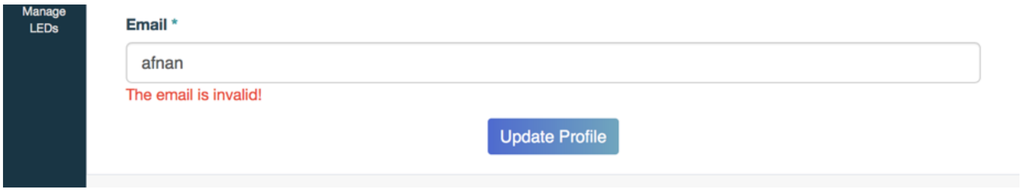			
**Pass?**			
Yes			

Table 29: Manage profile test case 3

Test Case: #3	Description	Action	Expected Result
**Enter incorrect password**	The user enters incorrect password.	Click on Reset Password	An error message will be displayed prompt the

			user to enter correct password
**Actual Result**			
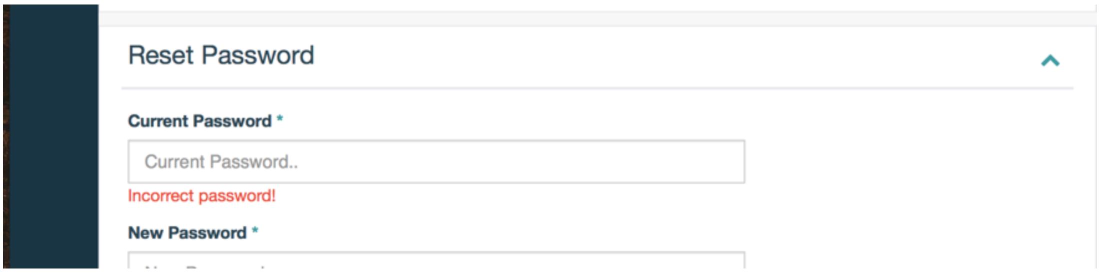			
**Pass?**			
Yes			

Table 30: Manage profile test case 4

**Test Case: #4**	**Description**	**Action**	**Expected Result**
**Enter incorrect confirm password**	The user enters confirm password that doesn’t match with the new password	Click on Press Reset Password	An error message will be displayed prompt the user to enter correct confirm password
**Actual Result**			
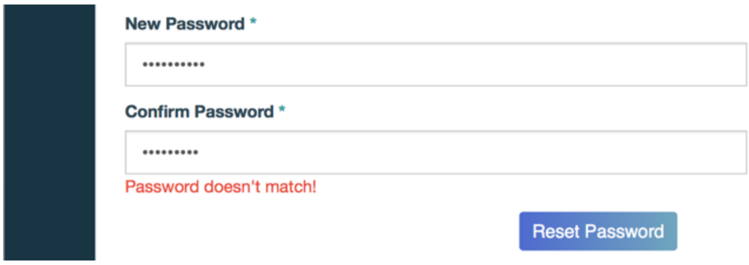			
**Pass?**			
Yes			

Table 31: Manage profile test case 5

7.3.2. APPLICATION

1. Register Test Cases

**Register Test Cases**					
**Test case#1**	**Description**	**Action**	**Expected Result**	**Actual Result**	**Pass?**

**Enter valid inputs**	The user enters valid information	Click on Register button	A new account will be created and the user will be redirected to the main activity.	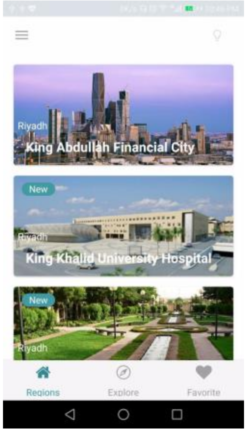	Yes
**Test case#2**	**Description**	**Action**	**Expected Result**	**Actual Result**	**Pass?**
**Leave empty fields**	The user leaves some or all fields empty	Click on Register button	An error message will be displayed prompting the user to complete all fields.	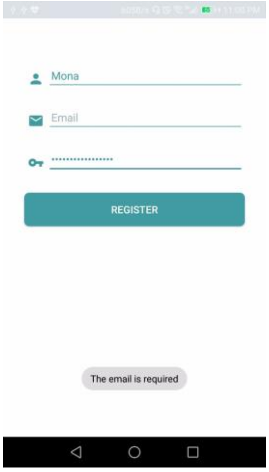	Yes
**Test case#3**	**Description**	**Action**	**Expected Result**	**Actual Result**	**Pass?**
**Enter invalid email**	The user enters an email in an incorrect format.	Click on Register button	An error message will be displayed prompting the user to enter a valid email.	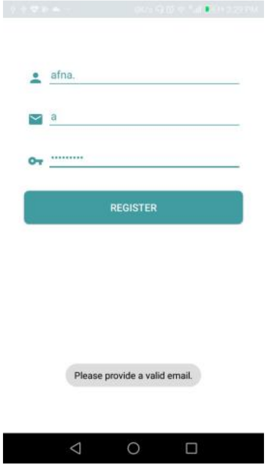	Yes
**Test case#4**	**Description**	**Action**	**Expected Result**	**Actual Result**	**Pass?**

**Enter email that is already registered**	The user enters a registered email	Click on Register button	An error message will notify the user that the email is already registered.	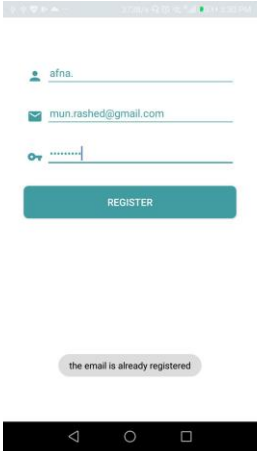	Yes

Table 32: Application register test case

2. Log in Test Cases

Log in Test Cases					
**Test case#1**	**Description**	**Action**	**Expected Result**	**Actual Result**	**Pass?**
**Leave empty fields**	The user leaves some or all fields empty	Click on Log in button	An error message will be displayed prompting the user to complete all fields	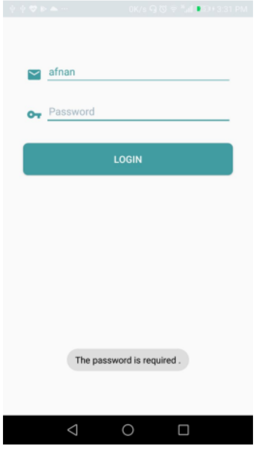	Yes
**Test case#2**	**Description**	**Action**	**Expected Result**	**Actual Result**	**Pass?**

**Enter invalid email or password**	The user enters invalid inputs	Click on Log in button	An error message will notify the user that the email or password is incorrect	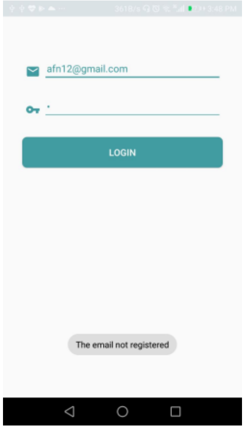<br>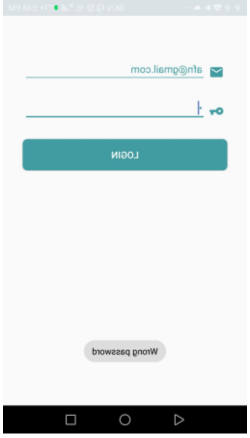	Yes

Table 33: Application log in test case

3. Edit Profile Test Cases

Edit profile Test Cases					
**Test case#1**	**Description**	**Action**	**Expected Result**	**Actual Result**	**Pass?**
**Leave empty fields**	The user leaves some or all fields empty	Click on save changes button	An error message will be displayed prompting the user to complete all fields	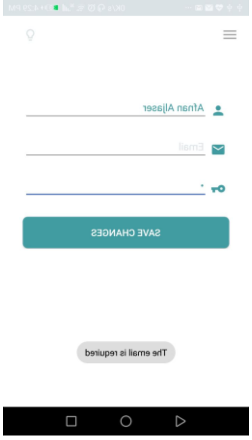	Yes
**Test case#2**	**Description**	**Action**	**Expected Result**	**Actual Result**	**Pass?**

**Enter invalid email**	The user enters invalid email	Click on Update Profile button	An error message will be displayed prompt the user to enter a valid email	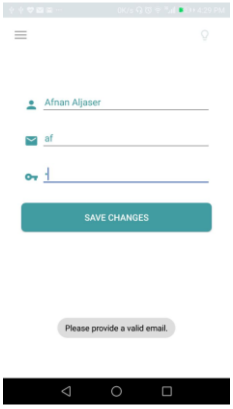	Yes

Table 34: Application edit profile test case

4. Explore Test Cases

Explore Test Cases					
**Test case#1**	**Description**	**Action**	**Expected Result**	**Actual Result**	**Pass?**
**Click on LED Feed (in range of a LED)**	The user clicks on LED Feed when he is in a range of a LED	Click on LED Feed	The linked admin content will be displayed.<br>If no content linked with the LED, a message will be displayed indicating that no content has been linked with the LED.	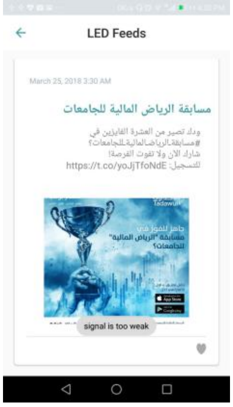	Yes
**Test case#2**	**Description**	**Action**	**Expected Result**	**Actual Result**	**Pass?**
**Click on LED Feed (not in range of a LED)**	The user clicks on LED Feed when he isn't in a range of a LED	Click on LED Feed	An error message will be displayed indicating that there is no Li-Fi signals found.	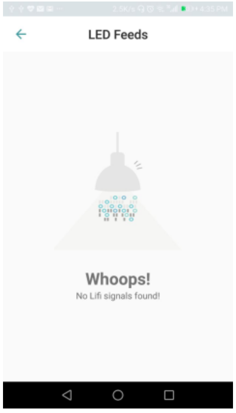	Yes
**Test case#3**	**Description**	**Action**	**Expected Result**	**Actual Result**	**Pass?**

**Add content to favorite list**	The user add a content to his favorite list	Click on heart icon	The selected content will be saved in the user's favorite list	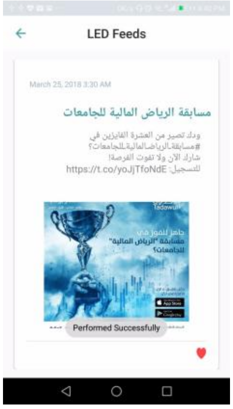	Yes
**Test case#4**	**Description**	**Action**	**Expected Result**	**Actual Result**	**Pass?**
**Click on Timeline (in range of a LED)**	The user clicks on Timeline when he is in a range of a LED	Click on Timeline	The linked user content will be displayed.<br>If no content linked with the LED, a message will be displayed indicating that no content has been linked with the LED.	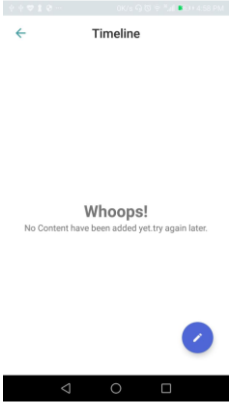	Yes
**Test case#5**	**Description**	**Action**	**Expected Result**	**Actual Result**	**Pass?**
**Click on upload content button in Timeline**	The user clicks on upload content button	Click on upload content button	The user will be redirected to upload content activity.	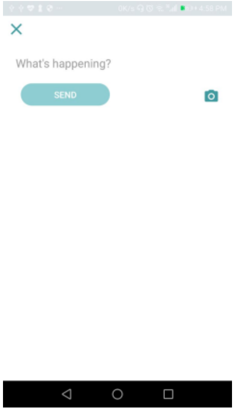	Yes
**Test case#6**	**Description**	**Action**	**Expected Result**	**Actual Result**	**Pass?**

**Click on Timeline (not in range of a LED)**	The user clicks on Timeline when he isn't in a range of a LED	Click on Timeline	An error message will be displayed indicating that there is no Li-Fi signals found.	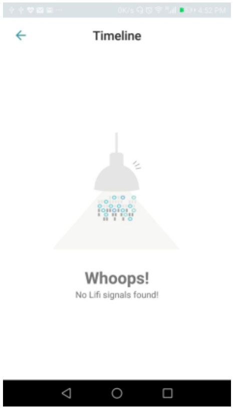	Yes

Table 35: Application main activity test cases 1

5. Favorite List Test Cases

**Favorite list Test Cases**					
**Test case#1**	**Description**	**Action**	**Expected Result**	**Actual Result**	**Pass?**
**View favorite list (logged in)**	The user clicks on Favorite when he is logged in.	Click on Favorite	The user's favorite list will be displayed.	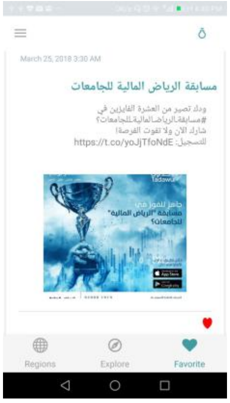	
**Test case#2**	**Description**	**Action**	**Expected Result**	**Actual Result**	**Pass?**
**View favorite list (not logged in)**	The user clicks on Favorite when he isn't logged in.	Click on Favorite	An error message will be displayed prompting the user to log in to view his favorites list.	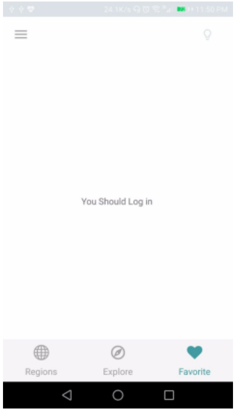	Yes
**Test case#3**	**Description**	**Action**	**Expected Result**	**Actual Result**	**Pass?**

**Remove content from favorite list**	The user delete a content from his favorite list	Click on heart icon of a content	The content will be removed from the list.	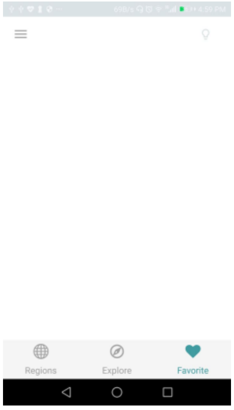	Yes

Table 36: Application main activity test case 2

6. Upload Content Test Cases

Upload content Test Cases					
**Test case#1**	**Description**	**Action**	**Expected Result**	**Actual Result**	**Pass?**
**Enter valid inputs**	The user enters valid inputs	Click on Share button	The content will be linked with the LED, and the user will redirected to the Timeline	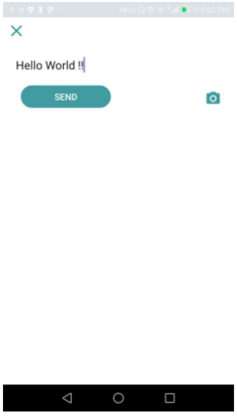	Yes

Table 37: Application upload content test cases

7.4. PERFORMANCE AND STRESS TESTING

7.4.1. PERFORMANCE TESTING

Performance testing is a type of testing intended to determine the responsiveness, throughput and reliability of an application under a given workload. It’s typically done to help establish a baseline to estimate the hardware configuration required to support the application when it goes live to production operation [49].

a. Test Environment

Android Profiler is a tool in android studio to provide real-time data for the application CPU, memory, and network activity. It is used to perform sample-based method tracing to time the application code execution, capture heap dumps, view memory allocations, and inspect the details of network-transmitted files [50].

Hardware used:

1. Huawei P9 Plus CPU: Hisilicon Kirin 995, RAM 4.0 GB, Android version 7.0
2. Geo-LiFi LED
3. Dongle

b. Performance acceptance criteria

1. Ensure the app under test is running in parallel with other apps; there should be no interference.
2. Ensure realistic user experience under several network conditions.
3. Ensure the application does not get crashed.
4. Ensure the RAM is required for utilizing this app does not exceed 10% of the mobile RAM.

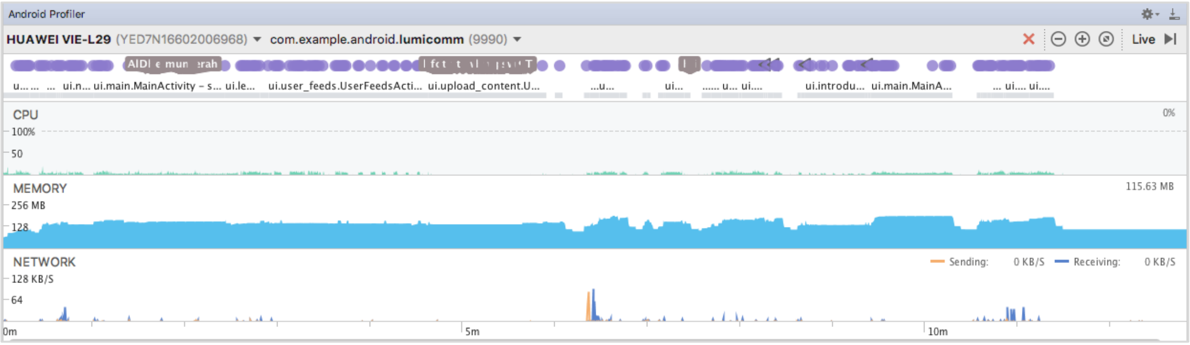

a. Test results

During the run time of 15 min, we found that highest memory usage was 194.8MB, the lowest is 72MB and the average was 150MB. From these results we can see that the RAM does not exceed 10% of the mobile app so, we can safely say that the application is light on the devices memory. Also We tried to run the application in parallel with others apps and this does not affect in the app performance. In addition,

Figure 1:application performance

we tried to perform all application functionalities and the application never crashed. Finally, the app gave a realistic user experience with a slow Internet connection, the

app receives contents but, the only multimedia contents take more time to appear on the screen. From the previous result we found that our application passed the performance testing.

7.4.2. STRESS TESTING

Stress testing determines the upper limits and the sizing of infrastructure by causing the application or its supporting infrastructure to fail. It is hard to perform stress testing manually, since it is difficult to simulate multiple user inputs and interrupts fast enough to strain the system. However, with the use of tools it can simulate these test scenarios easily. To perform stress testing, Neoload tool is used. Neoload is an automated test tool for measuring applications and web and API performance. Using Neoload tool, two tests have been applied to measure our application's performance.

a. First result

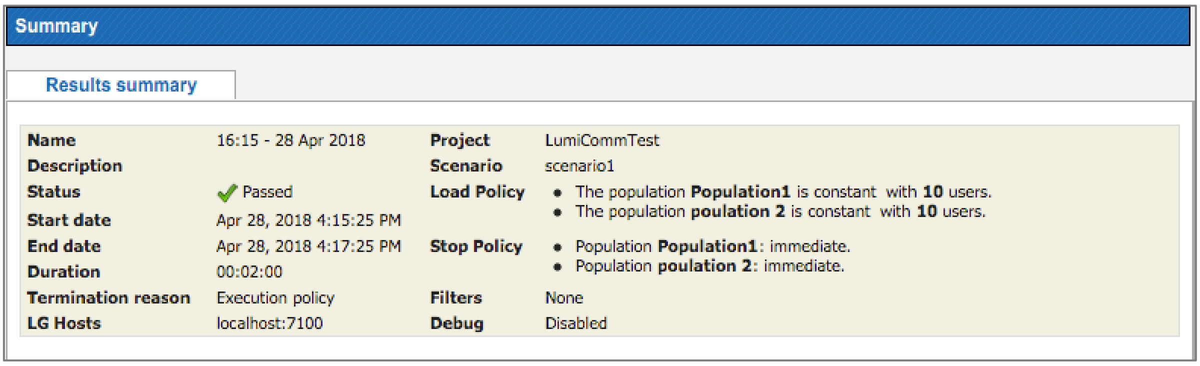

Summary

Results summary

**Name**	16:15 - 28 Apr 2018	**Project**	LumiCommTest
**Description**		**Scenario**	scenario1
**Status**	Passed	**Load Policy**	• The population **Population1** is constant with **10** users.<br>• The population **poulation 2** is constant with **10** users.
**Start date**	Apr 28, 2018 4:15:25 PM		
**End date**	Apr 28, 2018 4:17:25 PM	**Stop Policy**	• Population **Population1**: immediate.<br>• Population **poulation 2**: immediate.
**Duration**	00:02:00		
**Termination reason**	Execution policy	**Filters**	None
**LG Hosts**	localhost:7100	**Debug**	Disabled

Figure 2 : first stress test result

It can be seen in Figure 107, that our application has passed the first test, under the user of 20 users within two minutes. 20% of the users used Motoral X android level 6.0, 30 % used Samsung Galaxy S7 android level 6.0 and the last 50% used Samsung Galaxy S6 Android level 5.0.

b. Second result

Results summary

**Name**	17:30 - 28 Apr 2018	**Project**	LumiCommTest
**Description**		**Scenario**	scenario1
**Status**	✔ Passed	**Load Policy**	• The population **Population1** is constant with **20** users.<br>• The population **poulation 2** is constant with **20** users.
**Start date**	Apr 28, 2018 5:31:02 PM		
**End date**	Apr 28, 2018 5:46:03 PM	**Stop Policy**	• Population **Population1**: immediate.<br>• Population **poulation 2**: immediate.
**Duration**	00:15:00		
**Termination reason**	Execution policy	**Filters**	None
**LG Hosts**	localhost:7100	**Debug**	Disabled

Figure 3: second stress test result

As we can see in Figure 108, the application handled a heavy load. The number of users have been increased from 20 to 40 active users, and the duration time has been increased to 15 min. The application showed no signs of bugs, or memory leaks and did not crash from the load.

7.5. USER ACCEPTANCE TESTING

The objective of the user acceptance testing is to confirm that the system under test meets its requirements and to provide confidence that the system works correctly and measure its usability before it is delivered to the end users [54].

The usability of the system was measured in three criteria:

- **Effectiveness**: by measuring the number of errors detected when the user performs a specific function.
- **Efficiency**: by measuring the time that the user takes to perform a specific function.
- **Satisfaction**: by using a survey to discover the users' feedback about the system.

The total number of users who participated in the acceptance testing are ten users: five of them tested the website and the other five tested the application.

To test the efficiency and effectiveness of our system, the average time the users need to complete a specific task was computed and the average numbers of errors they have made was registered. The results are shown in Table.106 for the website and in Table.109 for the application.

To measure the user satisfaction of the system, a survey was made that measures different aspects of the system such as *ease of use and learnability*, *feedback and errors*, *consistency and screen displays*, *efficiency* and *subjective satisfaction*. The questions were constructed as seven-point rating scales. Users were asked to rate agreement with the statements, raging from strongly disagree to strongly agree. The questions were chosen based on several HCI rules measuring usefulness, reliability, usability, consistency, learnability, robustness and satisfaction. The questions included in the survey are as appears in Table 104.

**Survey Questions**	
**Ease of Use and Learnability**	
**1**	It is easy to use
**2**	I easily remember how to use it
**3**	It require the fewest steps possible to accomplish what I want to do with it
**Feedback and Errors**	
**4**	I can recover from mistakes quickly and easily
**5**	System messages are meaningful and jargon free
**6**	It makes it difficult to make mistakes
**Consistency and Screen Displays**	
**7**	I can easily identify where I am
**8**	Suitable choice of screen and font colors
**9**	Widgets locations and colors are consistent across displays
**10**	Wording is consistent across displays

**11**	Icons and symbols reflects intended task
**12**	I don't notice any inconsistencies as I use it
**Efficiency**	
**13**	It does everything as I expect it to do
**14**	Shifting among windows is easy
**15**	Guidance information always available
**16**	I successfully accomplished tasks every time
**Subjective Satisfaction**	
**17**	I am overall satisfied with it
**18**	It works the way I want it to work
**19**	It is designed for all levels of users

Table 38: Survey Questions

7.5.1.WEBSITE TESTING

The table below shows the testing users ages and genders:

Participant No.	Age	Gender
1	28	Male
2	45	Male
3	31	Male
4	24	Female
5	26	Female

Table 39: Users' ages and gender1

Function	Average number of errors	Average time in seconds
Register	0.2	40.04
Log in	0	17.8
Update profile info	0	12
Display LED(s) on map	0	3
Display LEDs' Statistics	0	1

Add region	0	53.48
Add LED	0.2	128.68
Add content	0	88
Update region details	0	23
Update LED details	0.2	33.48
Update content details	0	66.76
Delete region	0	23
Delete LED	0	24.4
Delete content	0	52.4
Link content with LED	0	48.8
Log out	0	2

Table 40: Measuring system effectiveness(website)

The table above shows the average time users need to complete each function in the website and the average numbers of errors they have made in each function. It can be seen from the table that the average number of error occurred in *register* and *add LED* functions is 0.2, while it's 0 in the other functions. The *add region* function took 53.48 second on average, and the related functions such as: *update region details* and *delete region* took between 23 to 33.48 second to be completed. The *add LED* function took 128.68 second on average, and the related functions such as: *update LED details* and *delete LED* took between 24.4 to 74.64 second to be completed. Finally, the *add content* function took 88 second on average, and the related functions such as: *update content details, delete content* and *link content* took between 48.8 to 66.76 second to be completed. Notice that adding LEDs to the system took the highest amount of time since it requires more steps to accomplish. This could lead to the consideration of enhancing the interfaces to make them easier and more accessible. In addition, since the interfaces of the main functions (add\delete\update) for the regions, LEDs and content are consistent among all windows, there's a noticeable drop in the number of seconds taken to accomplish the tasks. For instance, the "update form" is identical to the "add form", due that, the number of seconds taken to add a region (53 seconds) is relatively higher than updating it (23 seconds). While performing the test, we noticed that the number of seconds taken by all the test users to accomplish the same task is almost the same. From this we conclude that the easiness of use of the system is in the same level for different types of user, regardless of their skills and background.

Managing LEDs time taken dropped from approximately 128 seconds in “Add” to 33 seconds in “Update”. Which means once the user got familiar with the interfaces the easiness of use increase.

It can also be seen that the number of errors encountered while completing any if the tasks is almost zero. This indicates the level of ease and learnability of the system.

As mentioned earlier, the user must fill a survey after testing any of the subsystems. Some of the significant survey results regarding the admin web-panel appear in the tables below.

<table>
<tr><th colspan="2">Significant Survey Results</th></tr>
<tr><th colspan="2">Ease of Use and Learnability</th></tr>
<tr><td>1</td><td>
1-2 Strongly disagree
3-5 Neutral
6-7 Strongly agree

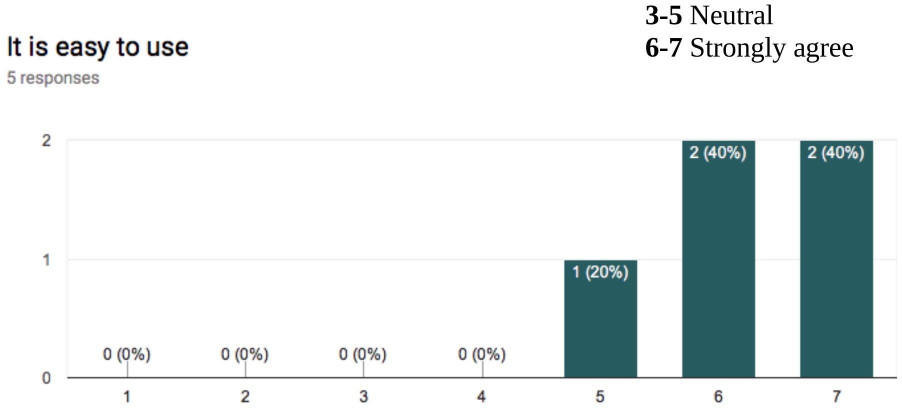

</td></tr>
<tr><th colspan="2">Feedback and Errors</th></tr>
<tr><td>2</td><td>
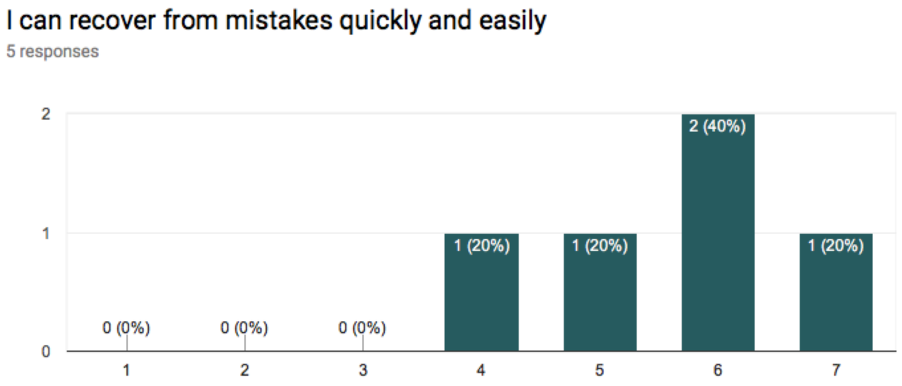

</td></tr>
<tr><th colspan="2">Consistency and Screen Displays</th></tr>
</table>

**3**	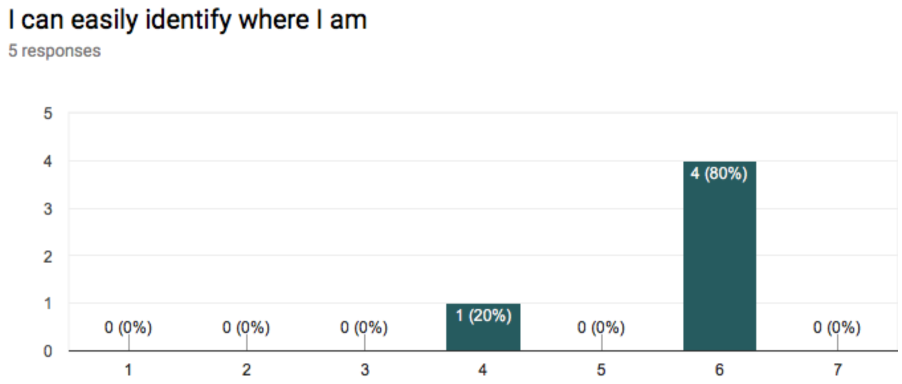
**Efficiency**	
**4**	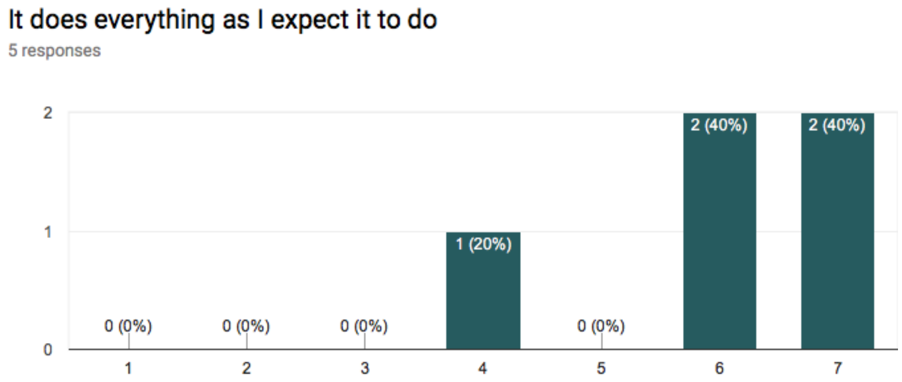
**Subjective Satisfaction**	
**5**	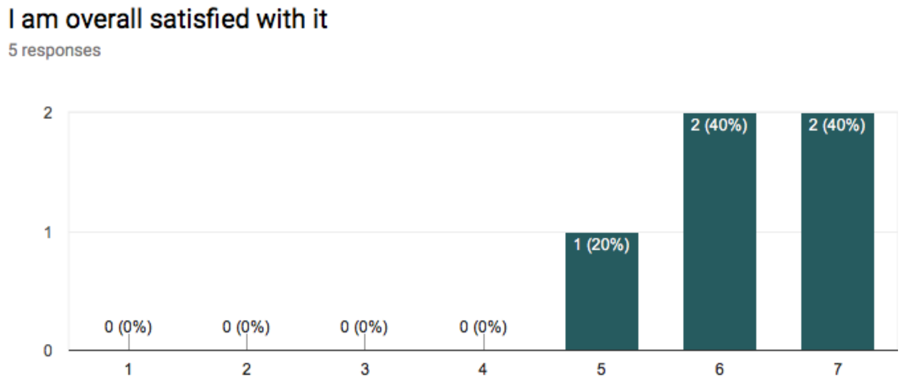

Table 41: Survey Results(website)

Regarding the results of the survey, the majority of users "strongly agree" that the website is easy to use, requires the fewest steps possible to accomplish the tasks and they can easily remember how to use it. This relates to the results obtained from measuring time taken to accomplish the tasks. Consistency plays an important rule to gain these results, as it entails the ease of use, learnability and familiarity. It can be seen that 3 out of 5 users "strongly agree" that they can recover from mistakes quickly and easily. This sentence concerns how the system notifies the user about incorrect

input, missing fields and unintended actions. Add to that, 4 out of 5 users "strongly agree" that the messages provided to them in case of mistakes are meaningful and jargon free. When considering the screen displays style and consistency among windows, all of the user "agree" that the choice of screen and font colors is suitable, widgets locations and colors are consistent across displays, wording is consistent across displays, icons and symbols reflects the intended tasks. That verifies the result concluded from analyzing the obtained measurement in Table 106, which is the acceptable level of consistency achieved throughout the pages.

However, most users reported that guidance information is not always available in all pages which may highlight the need to provide more guidance on how to use the system. Finally, 80% of users overall satisfied with it.

7.5.2. APPLICATION TESTING

The table below shows the users ages and genders:

Participant No.	Age	Gender
**1**	28	Male
**2**	24	Male
**3**	23	Female
**4**	22	Female
**5**	22	Female

Table 42: users ages and genders2

Function	Average number of errors	Average time in second
**Register**	0	40.2
**Log in**	0	19.5
**Edit profile**	0	28.1
**Upload content**	0.2	29.4
**View users content**	0	4
**View admin content**	0	5.2
**Save content to favorite list**	0	3.3
**View favorite list**	0	3.31
**View all regions**	0	2.2

**View region**	0	2

Table 43: Measuring system effectiveness(application)

The table above shows the average time that users need to complete each function in the application and the average numbers of errors they have made in each function. It can be seen from the table that the average number of error occurred in *upload content* is 0.2, while it's 0 in all the other functions. That could refer to the fact that uploading content requires more steps than other tasks. The *upload content* function took 29.4 second on average. In addition, *view users and admin content* took between 4 to 5.2 second on average, and the related functions such as: *save content to favorite list and view favorite list* took between 3.3 to 3.31 second. Finally, view all regions and view specific region took between 2 to 2.2 second on average. Registering in the system required the highest number of seconds among the functions. When the user first downloads the application, the first function to interact with is registration. According to that, it requires some time until the user gets familiar with how the application works; justifying the gained result. The other functions took noticeable low amount time to accomplish.

Regarding the results of the survey, most of users "strongly agree" that the application is easy to use. However, all of them "strongly agree" that it requires the fewest steps possible to accomplish the tasks, and they can easily remember how to use it; confirming learnability and ease of use of the application. Concerning robustness of the application, it can be seen that most of users "agree" that they can recover from any mistakes they make quickly and easily and the the provided messages are meaningful and jargon free. 90% of users "strongly agree" that the choice of screen and font colors is suitable, widgets locations and colors are consistent across displays, wording is consistent across displays and icons and symbols reflects the intended task. Furthermore, most users "strongly agree" that the application does everything they expect it to do, and shifting among windows is easy. However, 4 out of 5 users reported that guidance information is not always available. Moreover, most of users "strongly agree" that it works as the way they want it to work, and that it is designed for all levels of users. Finally, 80% of users overall satisfied with the application.
