# Supplementary material for: Towards smart sustainable cities using Li-Fi technology: geo-location infrastructure utilizing LED street lights
Source: PeerJ Comput Sci. 2022 Jul 21;8:e1009. doi: 10.7717/peerj-cs.1009 (PMC9454865; doi:10.7717/peerj-cs.1009)
Supplement: Supplemental Information 4 [file peerj-cs-08-1009-s004.pdf]

7.1. EXPERIMENT

7.1.1. LIFI REGION

Lumicomm is a system designed for smart cities, aiming to transform basic lights into a source of data. The scenarios where Lumicomm system could be deployed are various. In this imaginary experiment, King Abdullah Financial District (KAFD) is chosen as a region with Li-Fi LEDs installed. KAFD is a new development under construction near King Fahad Road in the Al-Aqeeq area of Riyadh, consisting of 59 towers in an area of 1.6 million square meters. It will provide more than 3 million square meters of space for various uses, 62,000 parking spaces and accommodation for 12,000 residents [51]. KAFD is designed to be divided into multiple areas as shown in Figure 114.

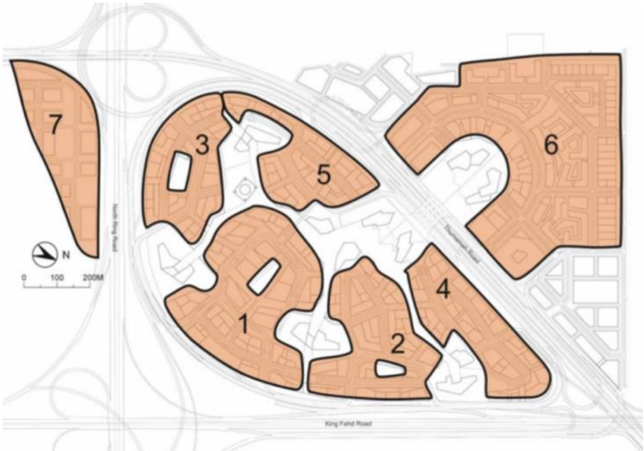

Figure 1: KAFD areas [52]

To accomplish the goal of transforming KAFD into a Li-Fi-enabled region, the process goes through four phases:

1. **Planning Phase:** includes the actual distribution and positioning of the LEDs, in addition to specifying the settings for each LED.
2. **Installation and deployment Phase**: this phase concerns the hardware installation in the areas specified in KAFD. In other words, this phase can be considered as the actual installation and creation of the regions.
3. **Execution Phase:** the logical installation and creation of the regions. In this phase, the actual locations of LEDs are mapped to the logical location in the system and grouped under the same regions for administrative tasks.
4. **Testing Phase:** the region is tested in this case from the second subsystem, which is the mobile application. The testing has been conducted according to different scenarios.

1. PLANNING PHASE

In the imaginary experiment, each of these areas will be equipped with multiple Li-Fi-enabled lighting solutions. Five area are chosen; the areas are as follow:

1. **The Financial Area**: This area is the center of KAFD where different international banks headquarters are located in addition to KAFD financial academy.
2. **The Residence Area:** this area consists of the residential buildings of KAFD and various global hotels.
3. **The Attraction Area**: the area groups the main attraction sites of KAFD, including: the science museum and geo-climate center, conference center, ACM Movie theater, the aquarium and festival market place and children's interactive museum.
4. **Monorail Area:** This area includes the main station of the monorail system of KAFD.
5. **Al-Wadi District Walk:** is an outdoor walking area of KAFD. The are consists of many restaurants, shops and cafes for the visitor of KAFD and it is designed to connect the different parts of KAFD in a walking area.

In each of these area, multiple Li-Fi LEDs have been installed. Those LEDs have been labeled with the area identifier and positioned in optimal locations to cover up most of KAFD subareas. The reason behind this distribution is to provide maximum flexibility in controlling LEDs' content, as each area is most likely to contain content different than the other when considering the nature of the majority of its users. For instance, Al-Wadi area may include many advertisements and proximal marketing content, in addition to guidance content for KAFD (maps, notification, announcements...etc). The distribution of LEDs among the areas appears in the table below. In total, KAFD has been equipped with **20** LEDs in the experiment.

Area#	Area Name	Main Sites LEDs are located at	# of LEDs equipped	LED unique IDs	Allow users to upload content?
01	Financial Area	SAMBA HQ<br>Financial Academy<br>KAFD World Trade Center	3	0x82f4	✕
				0x6390	✕
				0xfe22	✕
02	Residence Area	Residence Area<br>KAFD Grand Mosque<br>The Park	5	0x6e90	✓
				0x6a22	✓
				0x6a01	✓
				0xc9f0	✓

				0x72e1	✕
03	Attraction Area	The science museum and geo-climate center<br>Conference center<br>ACM Movie theater	3	0xf404	✓
				0xd206	✕
				0xd0e2	✓
04	Monorail Area	Station 1,2, and 4	3	0xc563	✓
				0x909c	✓
				0xc500	✓
05	Al-Wadi Area	Northern Al-Wadi<br>Western Al-Wadi<br>Southern Al-Wadi<br>Eastern Al-Wadi<br>Central Al-Wadi	6	0x6333	✓
				0x6e72	✓
				0x6f42	✓
				0x6e71	✓
				0xd5d5	✓
				0xe212	✓

Table 1: LED distribution among KAFD areas

As it can be seen in the table, the areas with high probability of having large number of visitors are most likely to allow user's to contribute in uploading their own content to the LEDs, such as Al-Wadi and the attraction areas. The other LEDs' content is managed by the administrator only.

The figure below illustrates how the LEDs have been distributed in area number 5. The purple dark lines represent the access areas of Al-Wadi.

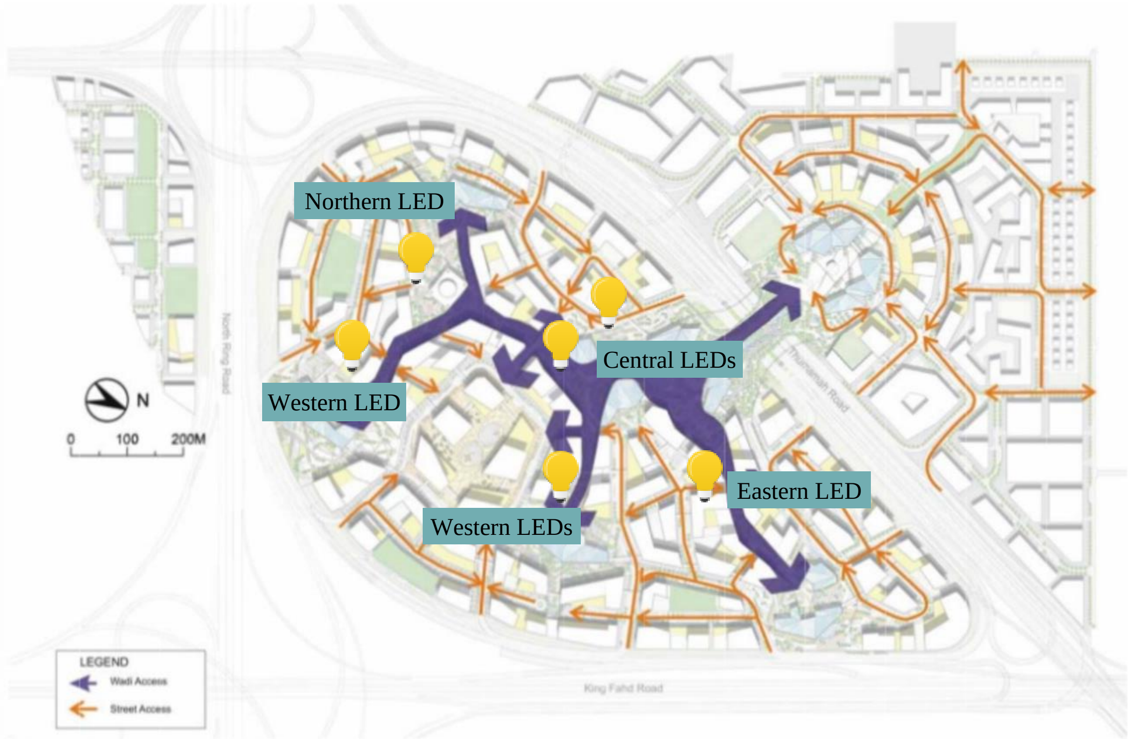

Figure 2: Al-Wadi access area in KAFD [52]

The other map appearing in Figure 112 shows where the LEDs have been situated in the monorail area (area#04). Station 1, 2 and 4 are where the LEDs have been installed.

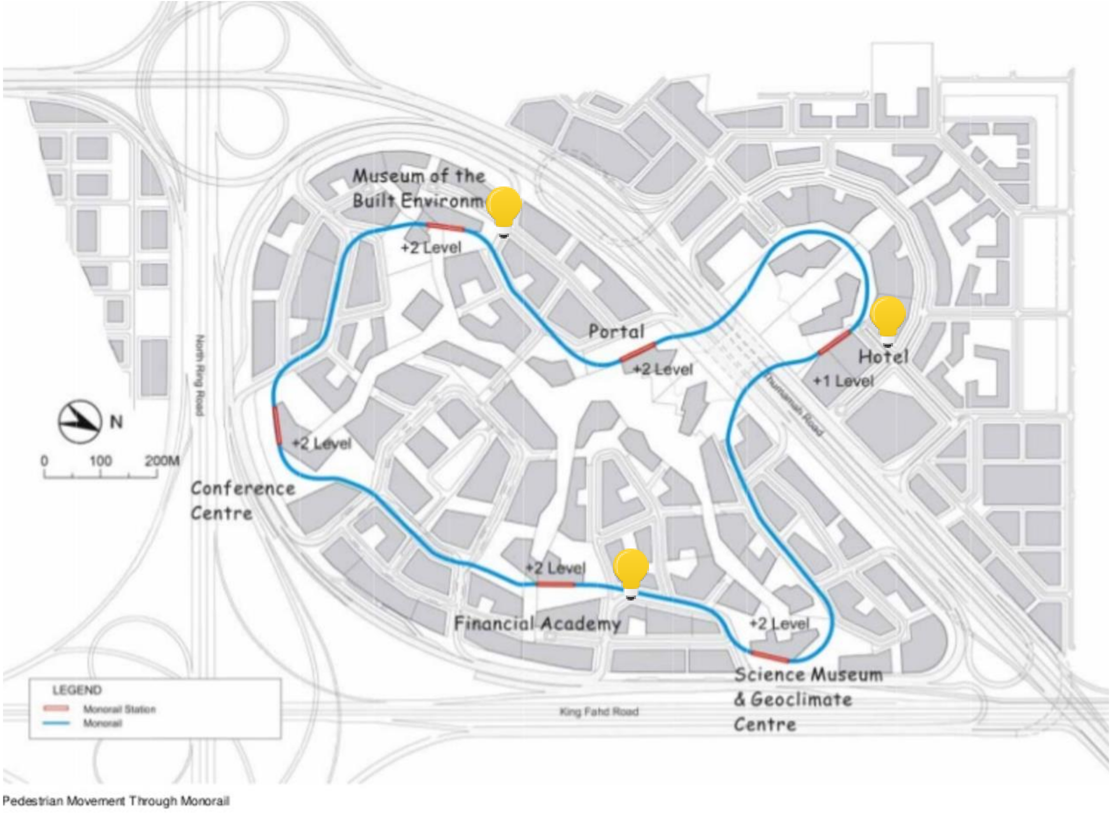

Figure 4: KAFD monorail area and stations [52]

As for the attraction area (i.e. area #03), LEDs have been installed in ACM Movie Theater (located near the conference center), Conference Center and The Science Museum and Geo-Climate Center. The LEDs locations appear in the map in Figure 116.

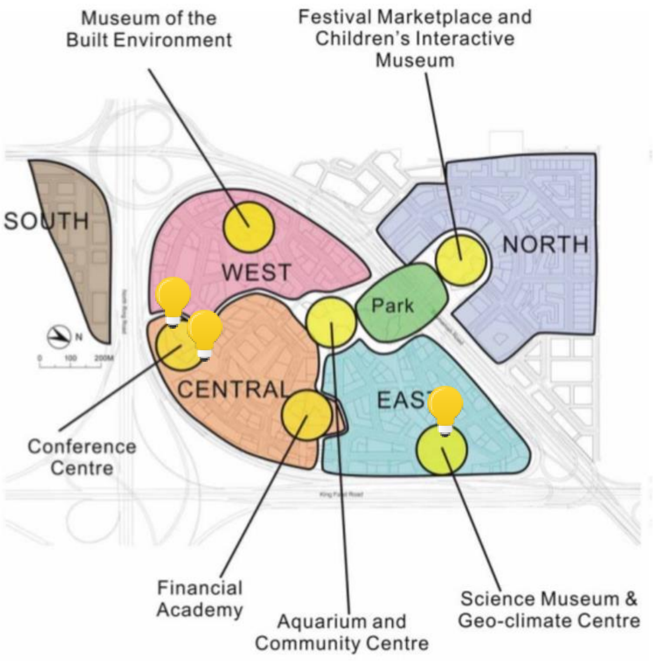

Figure 3: KAFD attraction areas [52]

2. INSTALLATION AND DEPLOYMENT PHASE

In this experiment, the Li-Fi enabled LED chosen for deployment in KAFD are manufactured by Technilum company and empowered by Oledcomm GeoLiFi transceiver chip. These lighting solutions belong to the GeoLiFi product range and they are compatible with both modulations: Li-Fi dongle and camera modulation. The streetlight comes in two heights 8m and 4m. The specifications of the streetlight are as follow:

- 1 Mbps downlink.
- 1 Mbps uplink.
- Headphone Jack Li-Fi dongle or camera modulation as signal receptor. It mainly depends on the equipped transceiver chip.
- Up to 4 m high LiFi detection.
- One user connected at a time.

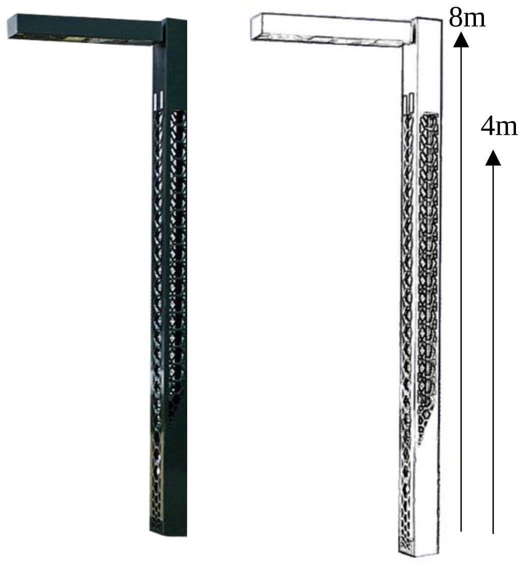

Figure 5: GeoLiFi streetlight

It is assumed that the coverage range of the light is 10m^2 [53].

3. EXECUTION PHASE

In this phase, the administrator maps LEDs actual locations to system’s logical locations. To accomplish this task, the admin has to do the following:

1. Register and login to Lumicomm Admin web-panel.
2. Create KAFD region with all the required details.
3. Insert LEDs and their information in KAFD region and map actual location in the map. The admin must also ensure uniqueness of LED identifiers (listed in Table 110).
4. Create the content and associate it with specific LEDs or spread it in KAFD region as whole.

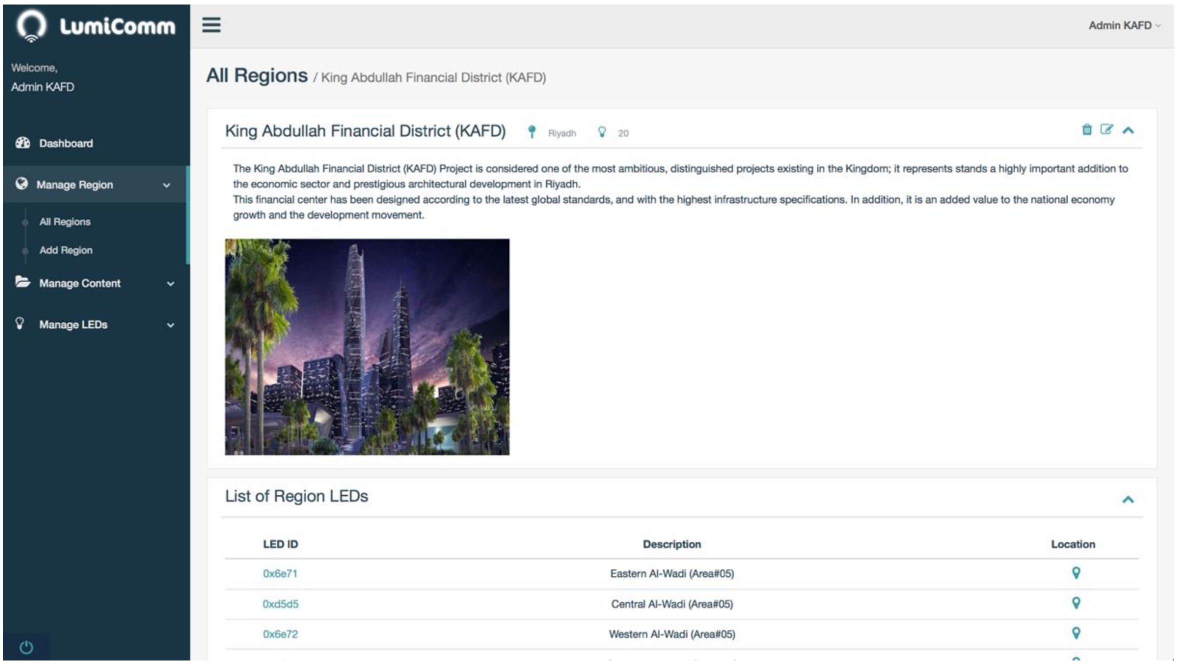

Figure 6: Screenshot of KAFD region in the admin web-panel

After the region have been created as it appears in Figure 114, the final distribution of LEDs' locations in KAFD can bee seen the map below captured from the admin web-panel.

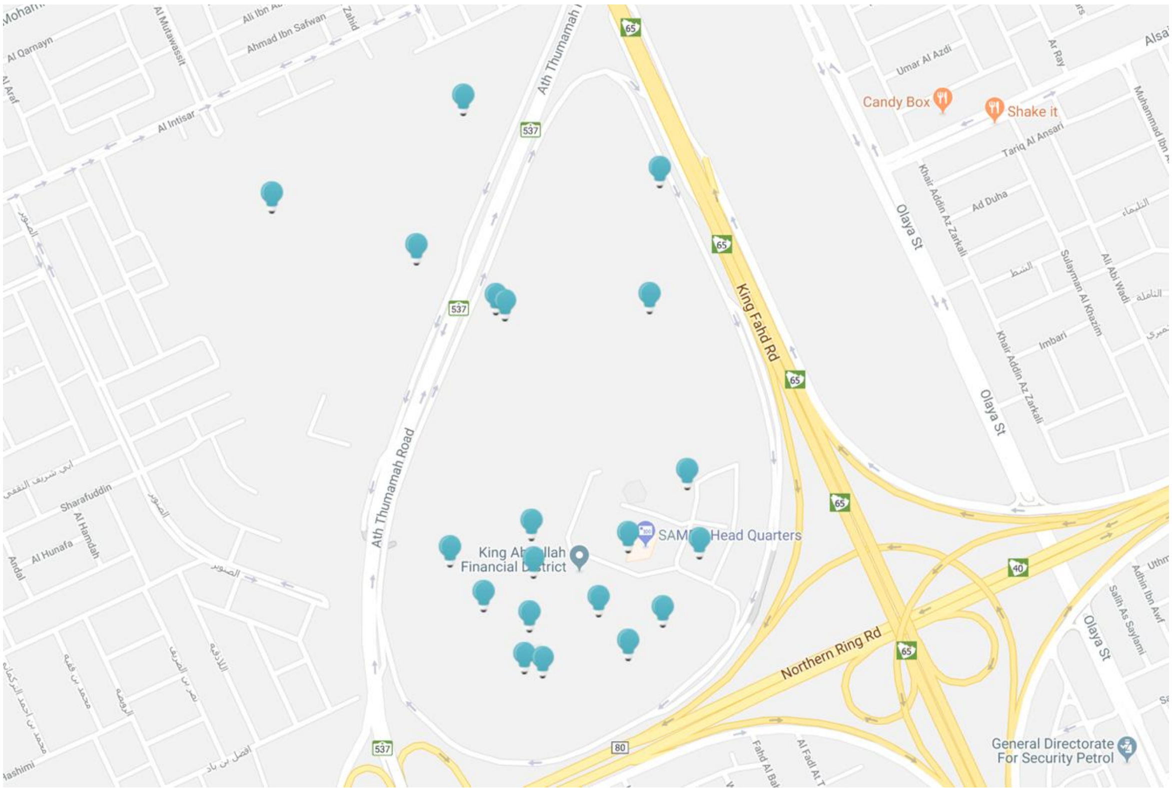

Figure 7: LEDs distribution (map view)

The content created by the admin appears in the screenshot below:

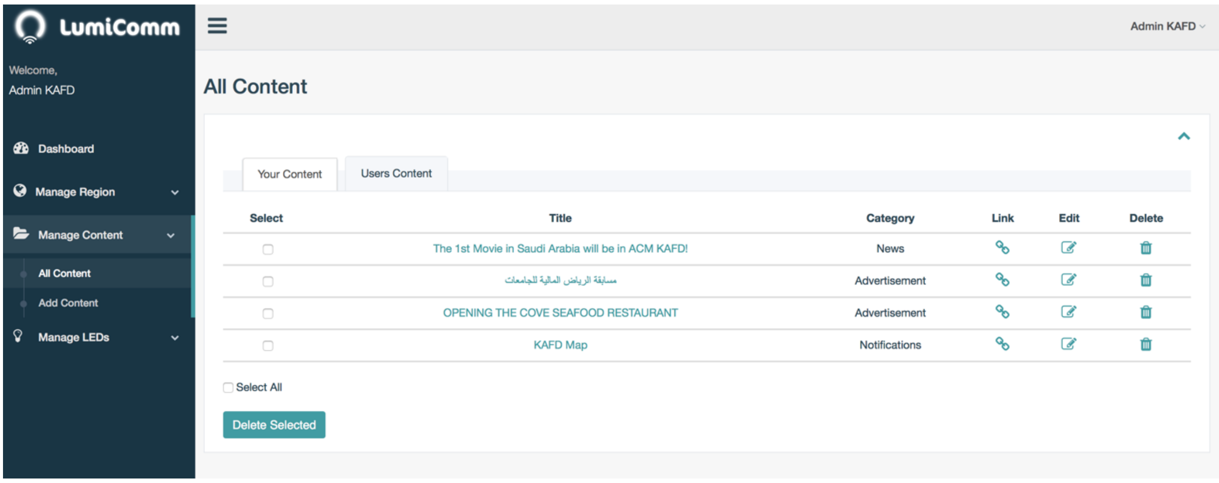

Figure 8: List of content created for KAFD

Viewing "مسابقة الرياض المالية للجامعات", notice that the content has been linked with 13 LEDs. Most importantly, it is linked with all the LEDs in the financial area as it appears in "Linked with" table.

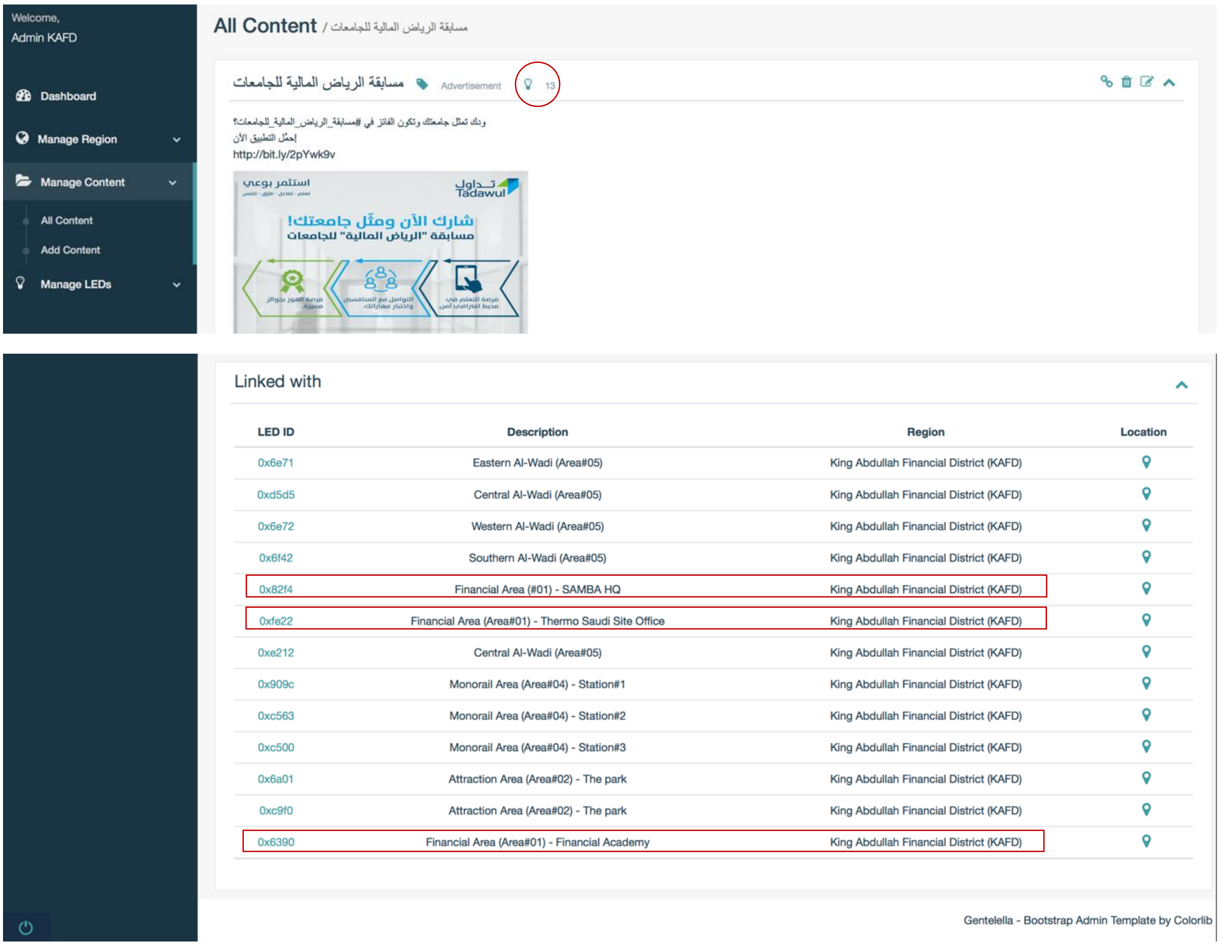

Figure 9: Content view

Now the region is created and the content has been added and linked with LEDs.

4. TESTING PHASE

In the testing phase, users are required to download Lumicomm Mobile Application to receive the content from the LEDs. Two LEDs placed in KAFD have been tested according to different scenarios: ACM Movie Theater LED and Al-Wadi Central LED.

4.1 LED#0xd5d5 - Central Al-Wadi

As assumed earlier, the coverage range of the GeoLiFi streetlight is approximately 10 m^2. therefore, when the user is in the coverage range the mobile phone will sense the Li-Fi signal and push notifications accordingly, as it appears in Figure 119 part (a). When the user opens the application, she/he can view the current region's information as appears in part (c). Part (b) shows the options available when the user visits the explore page, which are: LED feed, Timeline and Explore. It can be seen that the content linked with this LED is the movie news and "مسابقة الرياض المالية للجامعات" both created and linked by the admin in the previous step. As for the user's uploaded content, it appears in part (e).

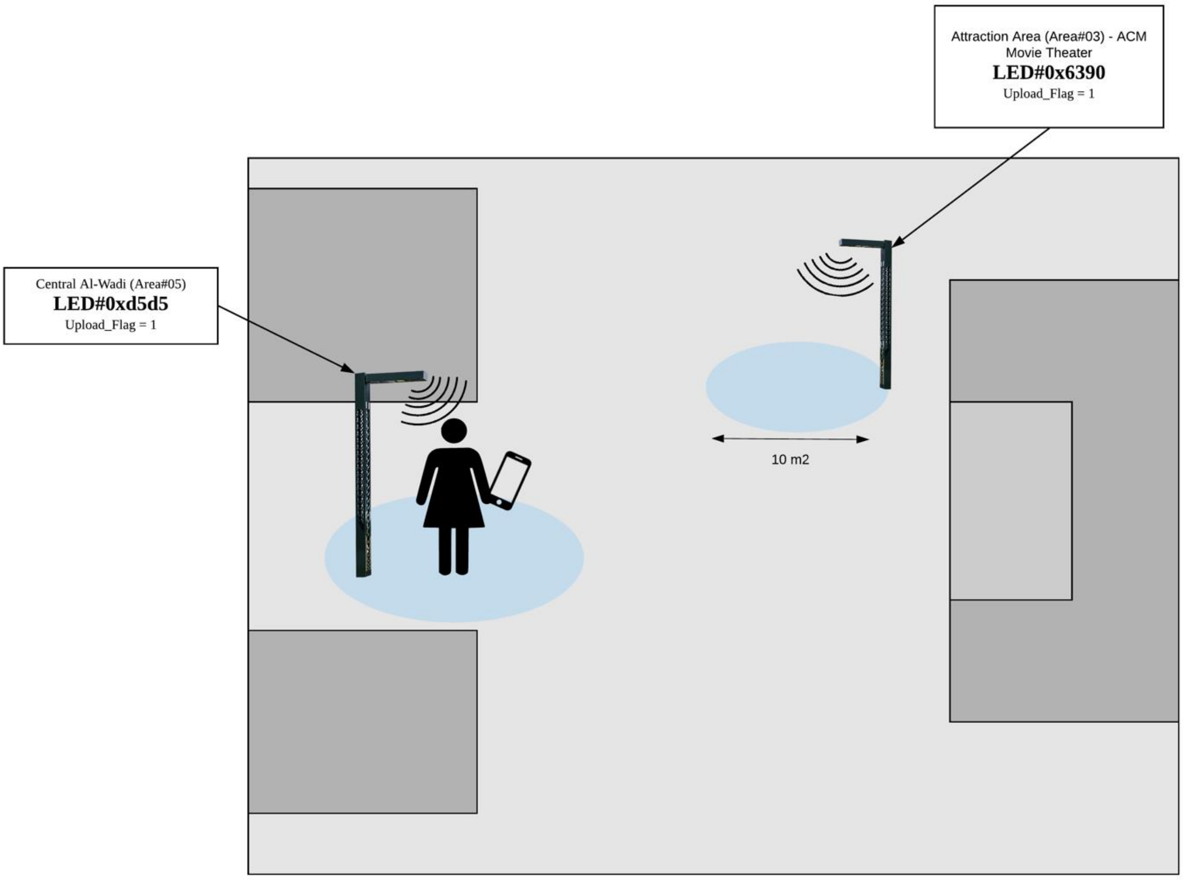

Figure 10: user inside the range of Al-Wadi LED

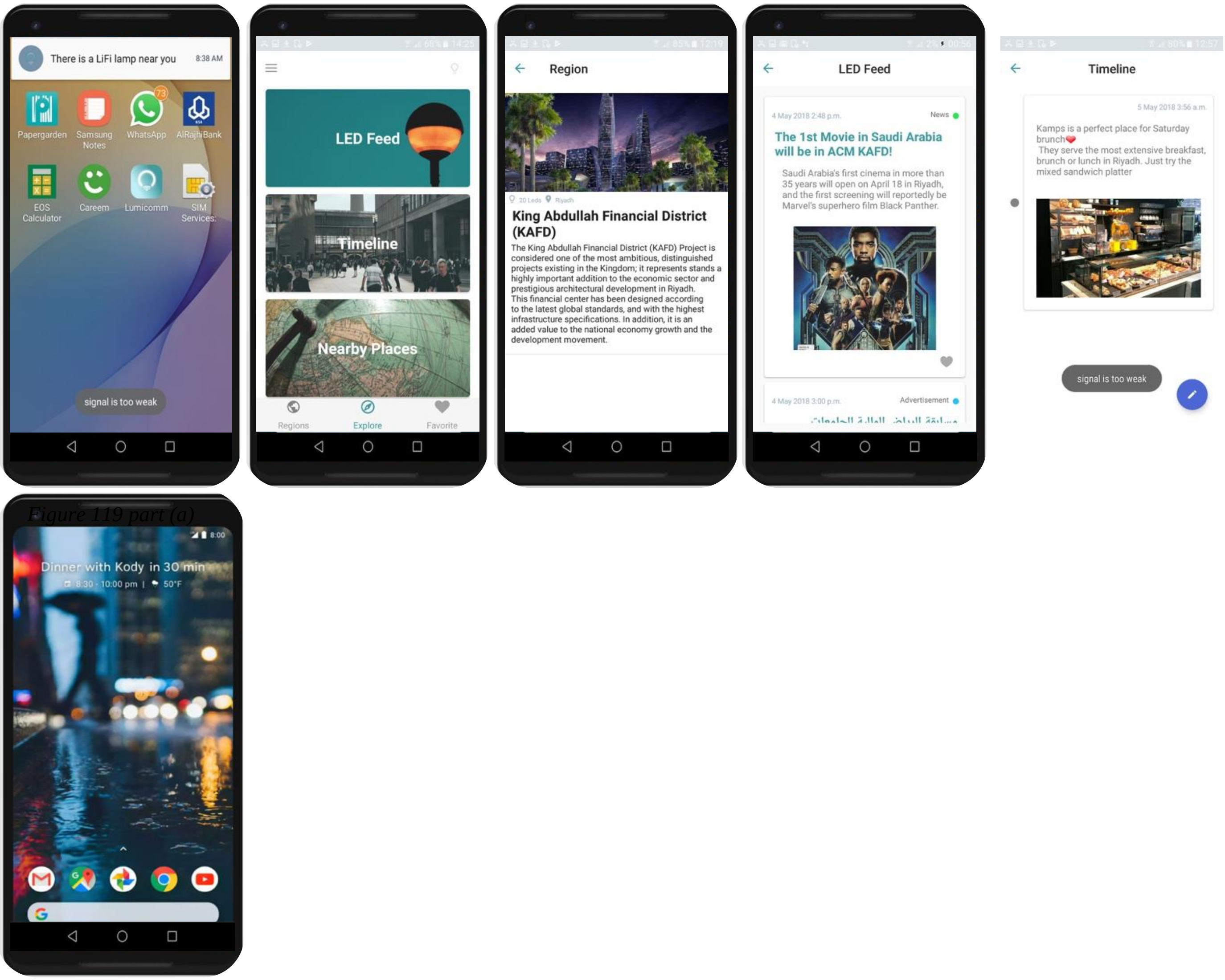

Figure 119 part (a)

Figure 119 part (b) *Figure 119 part (c)* *Figure 119 part (d)* *Figure 119 part (e)*

Figure 11: Interfaces of the mobile application inside the Li-Fi region

4.2 LED#0x6930 - ACM Movie Theater

When the user walks out of the LED range the screen will change to notify the user about being out of the Li-Fi detection perimeter.

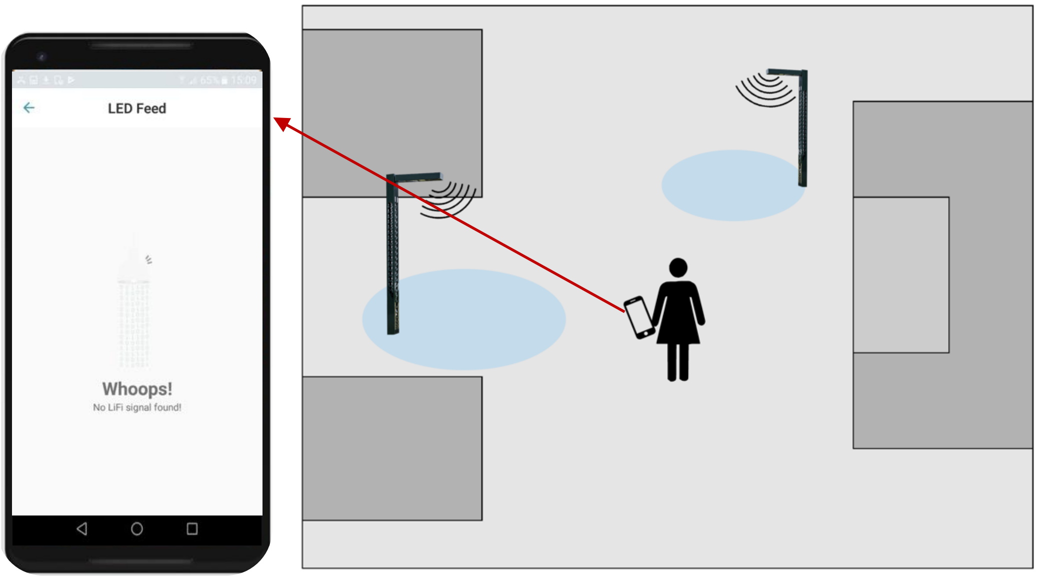

Figure 12: effects of the user walking outside the range of the Li-Fi LED

The user walks to the other LED near the ACM Movie Theater, the content will instantly change to the one's associated with LED#0x6390 shown in Figure 122. User can also contribute in uploading content to this LED since the upload flag is switched on by the admin. People have been posting about ACM Movie Theater in this LED. The explore tab provides an insight of the recommended nearby places around KAFD.

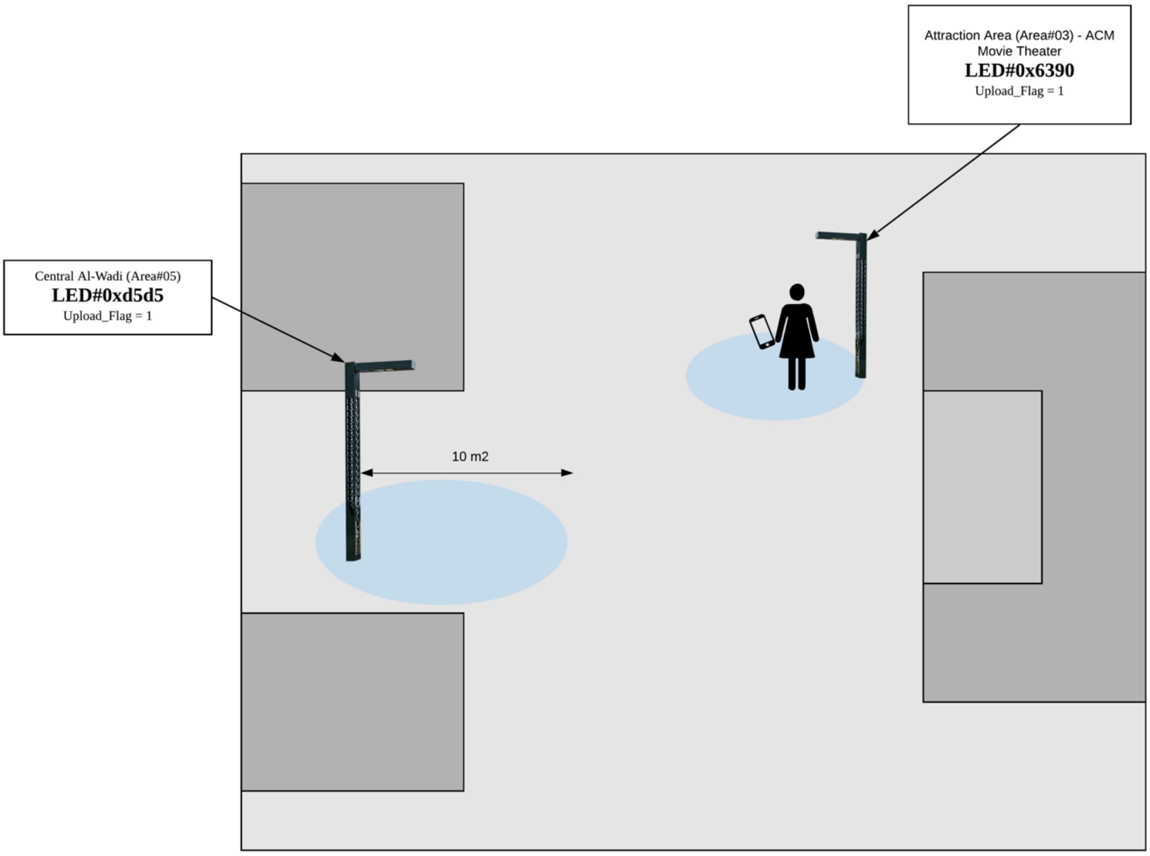

Figure 13: user in the range of the ACM Movie Theater LED

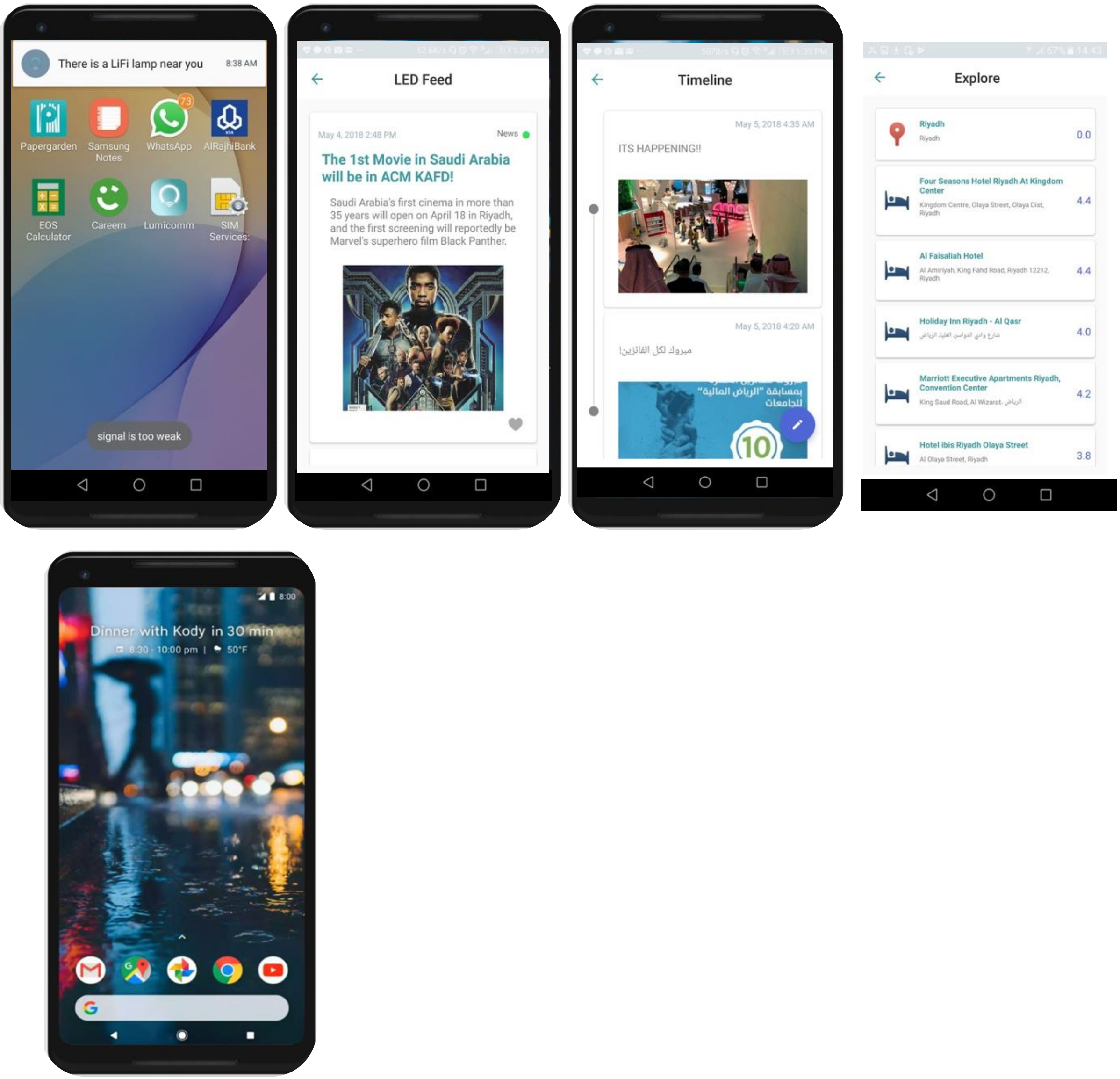

Figure 14: interfaces of the mobile application when the user enters the range of the LED

Since the admin linked the movie news with all the LEDs in KAFD, it appeared in both LEDs in the experiment.

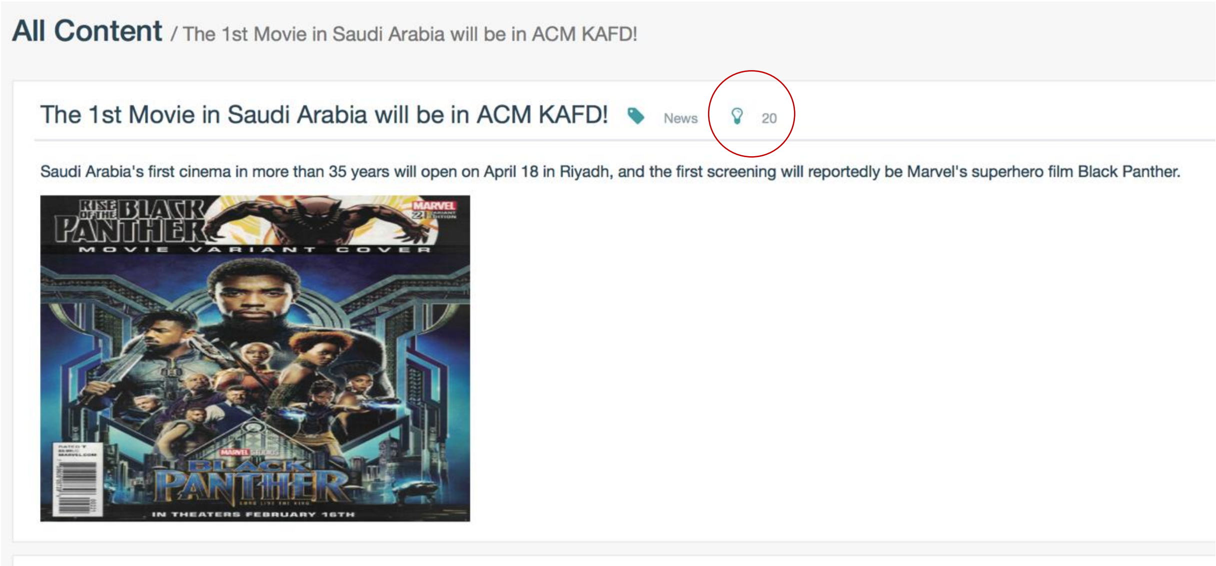

Figure 15: content that is linked with all LEDs in KAFD

The screenshot in Figure 124 provides insights of user interactions with the LEDs captured from the admin dashboard after running the system for a while in KAFD. There is 112 user content uploaded with LED 0x6e71 being the most visited LED in KAFD.

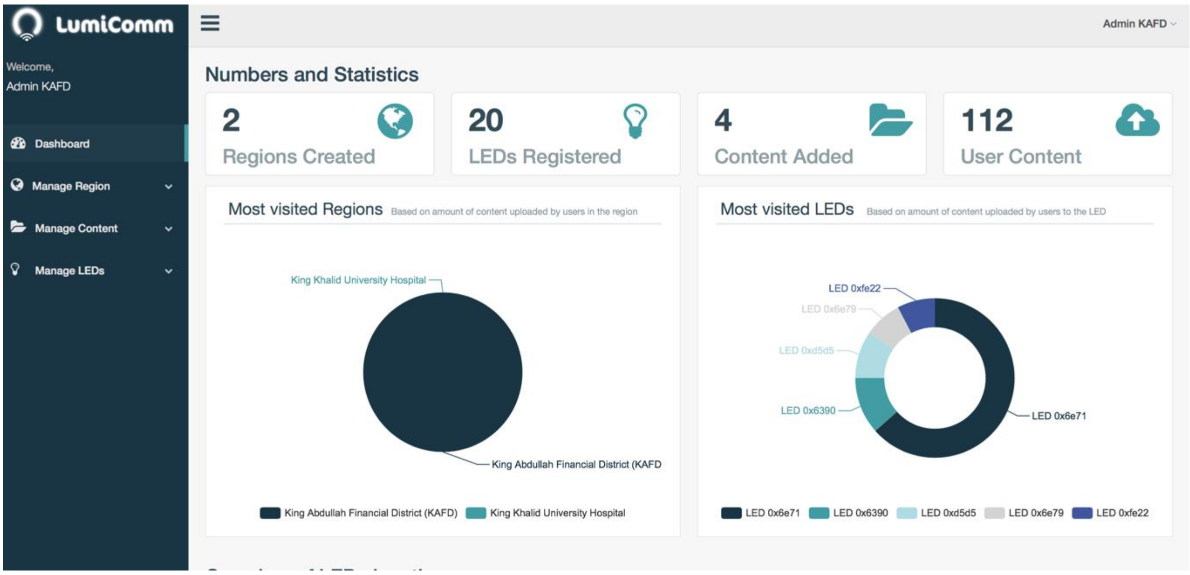

Figure 16: insights of user interaction with system's LEDs
