# Supplementary material for: Towards smart sustainable cities using Li-Fi technology: geo-location infrastructure utilizing LED street lights
Source: PeerJ Comput Sci. 2022 Jul 21;8:e1009. doi: 10.7717/peerj-cs.1009 (PMC9454865; doi:10.7717/peerj-cs.1009)
Supplement: Supplemental Information 5 [file peerj-cs-08-1009-s005.pdf]

Survey Questions	
**Ease of Use and Learnability**	
**1**	It is easy to use
**2**	I easily remember how to use it
**3**	It require the fewest steps possible to accomplish what I want to do with it
**Feedback and Errors**	
**4**	I can recover from mistakes quickly and easily
**5**	System messages are meaningful and jargon free
**6**	It makes it difficult to make mistakes
**Consistency and Screen Displays**	
**7**	I can easily identify where I am
**8**	Suitable choice of screen and font colors
**9**	Widgets locations and colors are consistent across displays
**10**	Wording is consistent across displays
**11**	Icons and symbols reflects intended task
**12**	I don't notice any inconsistencies as I use it
**Efficiency**	
**13**	It does everything as I expect it to do
**14**	Shifting among windows is easy
**15**	Guidance information always available
**16**	I successfully accomplished tasks every time
**Subjective Satisfaction**	
**17**	I am overall satisfied with it
**18**	It works the way I want it to work
**19**	It is designed for all levels of users
