# Supplementary material for: Towards smart sustainable cities using Li-Fi technology: geo-location infrastructure utilizing LED street lights
Source: PeerJ Comput Sci. 2022 Jul 21;8:e1009. doi: 10.7717/peerj-cs.1009 (PMC9454865; doi:10.7717/peerj-cs.1009)
Supplement: Supplemental Information 6 [file peerj-cs-08-1009-s006.pdf]

أسئلة الاستبيان	
سهولة الاستخدام والتعلم	
1	هل التطبيق سهل الاستخدام
2	أنا بسهولة أتذكر كيفية استخدام التطبيق
3	يتطلب التطبيق خطوات قليلة لتحقيق الخطوة المرجوة
الردود والأخطاء	
4	استطيع التراجع عن أي خطأ بسهولة وسرعة
5	رسائل التطبيق واضحة المعنى
6	من الصعب عمل أي خطأ عند استخدام التطبيق
الملائمة و عروض الشاشات	
7	أستطيع بسهولة تحديد مكاني في التطبيق
8	شاشات التطبيق مناسبة وكذلك ألوان الخطوط المستخدمة
9	جميع مكونات التطبيق من حيث اماكنها والوانها واضحة وبنفس التنسيق
10	الكلمات المستخدمة في التطبيق بنفس التنسيق في جميع شاشات العرض
11	الأزرار والرموز تعكس مهام التطبيق بوضوح
12	لم ألاحظ أي مشكلة في الملائمة والتنسيق في كل شاشات التطبيق
كفاءة التطبيق	
13	تعمل مهام التطبيق بالطريقة المتوقعة منها
14	التنقل بين شاشات التطبيق سهلة
15	معلومات ارشاد الاستخدام دائما متاحة
16	قمت بإتمام مهام التطبيق بنجاح في كل مرة
الرضا عن التطبيق	
17	أنا راضي عن التطبيق بشكل عام
18	يعمل التطبيق بالطريقة المتوقعة منه
19	تم تصميم التطبيق لمستويات مختلفة من المستخدمين
